# Dynamic Expression of Genes Involved in Proteoglycan/Glycosaminoglycan Metabolism during Skin Development

**DOI:** 10.1155/2018/9873471

**Published:** 2018-08-29

**Authors:** P. J. E. Uijtdewilligen, E. M. Versteeg, E. M. A. van de Westerlo, J. van der Vlag, W. F. Daamen, T. H. van Kuppevelt

**Affiliations:** ^1^Department of Biochemistry, Radboud University Medical Center, Radboud Institute for Molecular Life Sciences, Nijmegen, Netherlands; ^2^Department of Nephrology, Radboud University Medical Center, Radboud Institute for Molecular Life Sciences, Nijmegen, Netherlands

## Abstract

Glycosaminoglycans are important for cell signaling and therefore for proper embryonic development and adult homeostasis. Expressions of genes involved in proteoglycan/glycosaminoglycan (GAG) metabolism and of genes coding for growth factors known to bind GAGs were analyzed during skin development by microarray analysis and real time quantitative PCR. GAG related genes were organized in six categories based on their role in GAG homeostasis,* viz.* (1) production of precursor molecules, (2) production of core proteins, (3) synthesis of the linkage region, (4) polymerization, (5) modification, and (6) degradation of the GAG chain. In all categories highly dynamic up- and downregulations were observed during skin development, including differential expression of GAG modifying isoenzymes, core proteins, and growth factors. In two mice models, one overexpressing heparanase and one lacking C5 epimerase, differential expression of only few genes was observed. Data show that during skin development a highly dynamic and complex expression of GAG-associated genes occurs. This likely reflects quantitative and qualitative changes in GAGs/proteoglycans, including structural fine tuning, which may be correlated with growth factor handling.

## 1. Introduction

During various cell signaling processes, glycosaminoglycans (GAGs), such as heparan sulfate (HS), chondroitin sulfate (CS), and dermatan sulfate (DS), play a role in binding, guiding, and modulating signaling molecules,* e.g*., growth factors and morphogens [1–3]. In skin this role can be illustrated by the importance of GAGs in adult wound healing [2, 4] and in the extracellular matrix architecture formed during dermal development [5, 6]. A further example to illustrate the importance of GAGs comes from mice overexpressing heparanase, an enzyme involved in the degradation of HS, showing accelerated hair growth [7], indicating its involvement in hair follicle morphogenesis and homeostasis. Other observations show that HS is involved in hair follicle cycling, sebaceous gland morphogenesis, and homeostasis [8]. Finally, HS and heparanase influence wound healing in adult mice by enhancing keratinocyte migration and stimulating blood vessel maturation [9]. Taken together, GAGs play an important role in skin healing and development and this prompted us to evaluate the expression of GAG related genes during (embryonic) development in skin.

Inhibition of the expression of genes coding for enzymes involved in GAG modification reactions clearly indicates the importance of GAGs during organogenesis [10], especially with respect to growth factor handling. For example, mice deficient in Ndst1 (N-deacetylase sulfotransferase isoenzyme 1) die neonatally due to several defects in which defective sonic hedgehog (Shh) signaling is implicated [11, 12]; mice deficient in Hs2st (heparan sulfate 2-O sulfotransferase) or Glce (glucuronic acid epimerase) display renal agenesis [13, 14], whereas mice deficient in Hs6st1 (heparan sulfate 6-O sulfotransferase isozyme 1) show aberrant signaling of VEGF (vascular endothelial growth factor) and impaired lung development [15]. A skin phenotype of the above mouse models, however, has not been reported.

In general, it is thought that specific modifications of the GAG chain are involved in the binding and modulation of signaling molecules resulting in cell-type and/or tissue specific reactions [2, 3]. GAG mimetics like the RGTAs (regenerating agents) have been used to treat skin disorders and improve skin healing [16, 17]. To obtain insight in GAG metabolism during skin development we studied the expression of GAG related genes covering six functional classes ranging from the synthesis of precursor molecules to the synthesis and degradation of GAGs. In addition, we probed the expression of a number of (GAG binding) signaling molecules.

## 2. Materials and Methods

**Figure 1:**
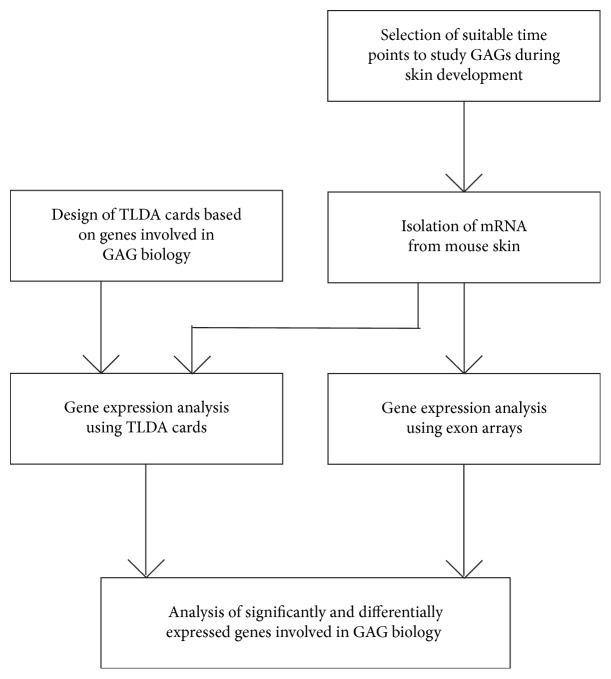
Experimental setup used for the analysis of gene expression involved in GAG biology during skin development in mice. Based on literature data, specific time points in skin development were selected. RNA was isolated, verified, and subsequently analyzed with GeneChip exon arrays and TLDA gene expression cards.

An overview of the experimental setup on the gene expression during murine skin development is given in Figure 1.

### 2.1. Animals for the Study on Skin Development

NIH guidelines for the care and use of laboratory animals (NIH publication 85–23 Rev. 1985) were followed. The study was approved by the Ethics Committee of the Radboud university medical center (DEC 2005-111, project: 81027). C57BL6/j mice were obtained from Elevage Janvier (Le Genest Saint Isle, France). Mice aged 90 days (90 days post birth [P90]) were used for timed mating and dorsal skin was collected at 14 days (E14) and 16 days after conception (E16). At E14 hair follicle development is initiated, and at E16 this process is almost completed in combination with a stratified epidermis and organized dermis [18, 19]. For the RNA samples of E14, dorsal skin of seven embryos from one female was pooled and used for RNA isolation. Skin was isolated at E14 by snap freezing the whole embryo in liquid nitrogen followed by scraping the skin layer in a cryomicrotome with a scalpel to minimize contamination with other embryonic tissues (skin is very thin at this time point). Samples were stored at -80°C. RNA samples for E16 were taken from two females, collecting dorsal skin form 7 embryos each. In addition, skin from 1-day old pups (P1) and adult mice (P90) was collected. At P1 skin is more organized and has been exposed to air [18, 19]. For the two dorsal skin samples for P1, three pups from two females were taken per sample. Two adult three-month old mice were used for the two dorsal skin samples at P90. Samples for RNA isolation for E16, P1, and P90 were collected by removing dorsal skin and snap freezing it in liquid nitrogen and storage at -80°C.

### 2.2. Tissue of Genetically Modified Mice

Skin samples of glucuronic acid epimerase (Glce) knockout mice (E18.5 for expression analysis; E17.5 and E18.5 for histological analysis) and of heparanase overexpression (Hpse) mice (P70) were provided by Prof. Dr. Jin-Ping Li (Department of Medical Biochemistry and Microbiology, University of Uppsala, Sweden) and Prof. Dr. Israel Vlodavsky (Vascular and Cancer Biology Research Center Rappaport Faculty of Medicine and Research Institute Technion-Israel Institute of Technology, Israel), respectively [7, 20]. For RNA isolation two wildtype and two mutant mice were used of both mouse models.

### 2.3. RNA Isolation, Real Time Quantitative PCR, and Microarray Analysis

Frozen samples were grinded in a micro-dismembrator (Sartorius, Bunnik, The Netherlands) and RNA was isolated using the TRIZOL-method (Invitrogen, Paisley, UK) in combination with RNeasy Mini kit with DNAse step (Qiagen, Hilden, Germany). RNA quality was assessed using the Bioanalyzer system (Agilent Technologies, Amstelveen, The Netherlands). The RNA integrity numbers (RIN, 27) were 8.8±0.25 (technical replicate N=2), 8.0±0.35, 8.5±0.55, and 7.3±0.2 for E14, E16, P1, and P90 (biological replicates N=2), respectively. The same procedure was used for the RNA isolation for the Glce knockout mouse and Hpse overexpression mouse. The RIN was 6.5±0.51 for* Glce*-/- samples and 8.0±0.48 for* Glce*+/+ and 6.3±0.3 and 7.7±0.6 for HPA-TG and HPA-WT, respectively (all biological replicate N=2).

Gene Chip Mouse exon 1.0 ST Arrays (Affymetrix, High Mycombe, UK) were used to analyze gene expression for E14, E16, P1, and P90 using 1 *μ*g of RNA per chip. Expression data were preprocessed to check sensitivity and specificity of the results based on Kadota et al. (2009) as shown in Uijtdewilligen et al. (2016) [18, 21]. Gene level expression data were calculated for the CORE transcripts (probe sets supported by RefSeq mRNAs) using Affymetrix Expression Console software with quantile normalization (all arrays are considered to have an equal intensity distribution), GC-content background correction (probes with high GC-content hybridize better, corrected for with built-in probes with different known GC-contents) and summarization with the RMA algorithm [22]. Data were imported into GeneSpring GX 7.3 (Agilent Technologies), duplicates were averaged, and the expression of each transcript was normalized to the median per array.

Real Time-Quantitative PCR (qPCR) was performed using custom designed Taqman Low Density arrays (TLDA) (Applied Biosystems, Nieuwerkerk aan de IJssel, the Netherlands) containing probes against genes involved in GAG metabolism and GAG binding proteins (Supplementary data Table 1). Glce and Hpse samples were analyzed using qPCR using custom designed TLDA with an adapted design containing additional GAG related genes (Supplementary data Table 2).

For the TLDA cards, 100 ng cDNA in Taqman Universal PCR Master Mix (Applied Biosystems) was loaded on the TLDA card per slot and run on a 7900HT Fast Real Time PCR System (Applied Biosystems). Expression was analyzed based on the threshold cycle (Ct) which was obtained using the SDS 2.3 software and RQ Manager 1.2 of Applied Biosystems using the combined expression data of the tested TLDA cards. In Microsoft Excel the reference genes for ΔCt calculation were checked for stability of expression by analyzing the results of the reference genes across all used TLDA cards and selecting the reference genes with the smallest deviation across the cards tested. Subsequently ΔCt values were calculated using a reference gene with the smallest difference between the average Ct found for the gene of interest and for the reference gene. The obtained ΔCt values were further processed using the 2^−ΔΔCt^ method using P90 as a calibration point in case of the developmental study and the wild type background (C57BL6 mice) data in case of the two mouse models [23].

### 2.4. Statistical Methods

Statistical significance of the exon array data was analyzed using ANOVA and Benjamini-Hoghberg multiple testing correction [24]. Statistical significance of the TLDA card data was tested with an unpaired T-test (2-tailed) using Microsoft Excel. Data with a statistical threshold of p<0.10 and a fold threshold of >2.0 were considered statistically significant.

## 3. Results

Genes involved in glycosaminoglycan (GAG) synthesis, modification, and degradation were studied during skin development at 14 and** 16 days **after** conception** (E14 and E16, respectively) and at one day after birth (P1) and compared to mature skin of a 3 month old mouse (P90). In addition, two mouse models, a Glce knockout mouse (E18.5) and an Hpse overexpression mouse model (P70), were analyzed. Taqman Low Density Array (TLDA) cards were designed to contain genes involved in GAG metabolism (Supplementary data Tables 1 and 2). The expression data obtained using TLDA cards and exon arrays were screened for genes with 2-fold differential expression at a statistical threshold of p<0.10 (Tables 1, 2, 3, and 4, Supplementary data Tables 4, 5, and 6).

In Tables 1–3 and Supplementary data Table 3, an overview is given of the differentially expressed genes applying TLDA cards and exon arrays. In all categories of genes involved in GAG metabolism, i.e., production of precursor molecules, core proteins, synthesis of linkage region, polymerization, modification and degradation of the GAG chain, and differences in expression were found (Tables 1–3). This indicates a highly dynamic expression pattern during skin development. Some isoenzymes were upregulated, whereas other isoenzymes were downregulated, further stressing metabolic complexity. This is, for instance, the case with GFPT1 and 2, both rates limiting enzymes involved in the production of hexosamines, and the isoenzymes HS 3-O sulfotransferase 6 and 3b1.

With respect to the core proteins, differential expression was found for both HS and CS/DS proteoglycans. Differential expression was found for two of the four syndecans,* viz. Sdc1* and* Sdc4*, three of the six glypicans,* viz. Gpc2, Gpc3*, and* Gpc6*, and* Hspg2* (Tables 2 and 3). The syndecans were downregulated, while the glypicans were upregulated, indicating an embryonic role for glypicans as described in literature [25, 26].* Hspg2*, a secreted HS presenting proteoglycan coding for perlecan [2], was found to be upregulated (Tables 2 and 3). Based on the exon array the CS/DS core protein of versican (Vcan) was upregulated at all time points (Supplementary data Table 5).

The upregulated expression of genes involved in the synthesis of the linkage region may signal increased GAG synthesis during development since after the formation of the linkage region the GAG chain is formed. For HS polymerization differential expression was found for,* e.g.*,* Extl1* and* Extl2*.* Extl1* showed downregulation at E14 while* Extl2 *was upregulated, and both enzymes are involved in the initiation and elongation of the HS chain [2].

During and after synthesis of the glycosaminoglycan chain, disaccharide units within the chain are specifically modified. These modifications determine which effector molecules can bind to the chain and thus play a role in cell signaling [1, 3]. Upregulated expression was found for three of the four N-deacetylase/N-sulfotransferases (*Ndst*; TLDA cards, Table 2), especially isoenzyme* Ndst3*. Upregulation was also found for two out of seven genes coding for 3-O-sulfotransferases (*Hs3st1 *and* Hs3st3b1*), involved in 3-O sulfation of GlcNS and GlnNAc residues, whereas one was downregulated (*Hs3st6)*.

The GlcNS and GlcNAc residues can also be 6-O sulfated by 6-O-sulfotransferases (Hs6st) [2] and selectively desulfated extracellularly by two sulfatases (*Sulf1* and* Sulf2*) aided by two cofactors (*Sumf1* and* Sumf2*) [2, 27].* Hs6st2* was upregulated at all time points (Table 3).* Sulf1* was upregulated during embryonic development, whereas* Sumf2* was upregulated at E14 (Tables 2 and 3). These results indicate that specific expression of GAG modifying enzymes may play a role in specific cellular signaling during skin development.

Within the class of genes encoding for GAG chain degradation enzymes, two genes were differentially expressed. Heparanase expression was downregulated at E14 and P1 (Tables 2 and 3), whereas N-sulfoglucosamine sulfohydrolase (*Sgsh*) was downregulated during embryonic development at E16 (Table 2) and at E14 (Table 3).

In addition to genes involved in GAG metabolism, the TLDA card contained 37 genes encoding growth factors, which were also present in the microarray (Tables 2 and 3). Differential expression was found by both TLDA card and microarray analysis for 10, 9 and 4 growth factors at E14, E16 and P1 respectively. Examples are insulin-like growth factor 2* (Igf2*), wingless-related integration site 6 (*Wnt6*), and* Wnt7b*.* Igf2* was dramatically upregulated at all time points, as expected based on previous research [18].* Wnt6* was also upregulated at all time points, while* Wnt7b* was upregulated only during embryonic development.

Next to their expression during development, gene expression of GAG-associated genes was studied in a* Glce* (glucuronyl epimerase) knockout mouse model and a heparanase overexpression mouse model using TLDA cards. In the* Glce* knockout mice six genes were differentially expressed (Table 4). Three of them are involved in CS and DS proteoglycans and were downregulated,* i.e.*, aggrecan (*Acan*), asporin (*Aspn*), and chondroitin sulfate N-acetylgalactosaminyltransferase 2 (*Csgalnact2*). Up/downregulation was not found for HS related genes, except for* Glce*, which was downregulated as expected. For the heparanase overexpression mouse model, in which a human heparanase was overexpressed [7], the results showed only one gene to be differentially expressed, i.e., aggrecan* (Acan)* which was 2.5-fold upregulated. The complete results of both the* Glce *knockout mouse and the Hpse overexpression mouse are given in Supplementary data Table 6.

## 4. Discussion

GAGs play a regulating role during embryonic development of various organs [1–3]. Therefore, we examined the expression of genes involved in GAG metabolism during skin development using custom designed Taqman Low Density Arrays (TLDA card) and exon arrays. To structure the data we studied gene expression in six functional classes,* viz.* the production of precursor molecules, the synthesis of core proteins and the linkage region, and the synthesis, modification, and degradation of the GAG chain proper. In addition we studied a number of growth factors, since GAGs are involved in their regulation including growth factor diffusion and signaling [3, 28].

With respect to core proteins, the heparan sulfate proteoglycans syndecan and glypican showed notable differential expression (Tables 2 and 3). Glypicans play an important role in development and cell signaling [12, 26, 29], and we found upregulation of 3 out of 6 glypican core proteins.* Gpc3* was upregulated during embryonic development and one day postbirth, suggesting that this glypican has a role during skin development. A possible function of* Gpc3* in skin has been suggested for the* Gpc3*-null mouse, which showed pigmentation defects [30]. Humans deficient in* Gpc3* suffer from the Simpson-Golabi-Behmel syndrome (SBGS). Based on the symptoms of SGBS and the phenotype found for the* Gpc3*-null mice, it has been suggested that Gpc3 is involved in the regulation of hedgehog signaling [31], a signaling pathway involved in hair follicle development [32]. Surprisingly, the* Gpc3*-null mice did not show a defect in appendage formation [30], indicating a functional but not essential role. Further research is needed to elucidate the role of* Gpc3* and the two other differentially expressed glypicans,* i.e., Gpc2* and* Gpc6*.

Syndecans are described to take part in adult wound healing [33]. We found downregulation of the core proteins of two syndecans during embryonic development, which could indicate that these proteoglycans do not play a major general role during skin development. Specific roles, such as the involvement of* Sdc1* in hair follicle development, as described on basis of immunohistochemical data [34], can, however, not be excluded.

In the class of GAG chain polymerization, we found differential expression of genes encoding for the initiation of HS or CS/DS synthesis. HS chain polymerization is initiated by the addition of GlcNAc by* Extl2* [35] or* Extl3* [36], while CS/DS chain polymerization is initiated by the addition of GalNAc by* Csgalnact1* [37].* Extl2 *was upregulated during early skin development (Table 2), while* Csgalnact1 *was downregulated (Supplementary data Table 5), which suggests that during early skin development HS production is stimulated in comparison to CS/DS production.

Enzyme mediated chemical modifications of the GAG chains result in the creation of specific binding sites for effector molecules [38]. Enzymes forming the class of N-deacetylase/sulfotransferases (Ndst's) are initiating elements in this respect. Especially* Ndst3* was upregulated, being one of four enzymes responsible for the removal of the acetyl group from the N-acetylated glycosamine and for the addition of a sulfate group. The additional expression of* Ndst3 *in combination with* Ndst1* and* Ndst2 *points to the fine tuning of HS chains for specific recognition of ligands.* Ndst3* has a higher deacetylation activity in comparison to the N-sulfotransferase activity, while* Ndst1* and* Ndst2* have a slightly higher N-sulfotransferase activity [39]. In addition, the data on the expression of heparan sulfate 3-O sulfotransferases (Hs3sts) [40] and heparan sulfate 6-O-sulfotransferases (Hs6sts) [41] suggest dynamic and specific modification of HS chains.

Three genes encoding for enzymes involved in HS and CS/DS degradation were differentially expressed, one of them being* Hpse* (heparanase).* Hpse* is downregulated at E14 and at P1, but not at E16 at which time point hair follicle development is taking place.* Hpse* has been reported to be involved in this process [42, 43].

Glycosaminoglycans are involved in growth factor regulation during developmental processes [1, 2]. We therefore studied 37 growth factors implied in skin development. A number of genes encoding growth factors were differentially expressed during development and the data are in line with earlier results for, e.g.,* Igf2* [18],* Wnt6*, and* Wnt7b* [44]. Although speculative, the dynamics in GAG structure may be correlated with the dynamics of growth factors.

Next to skin development we also studied gene expression in skin of a* Glce* (glucuronyl epimerase) knockout mouse and an Hpse (heparanase) overexpression mouse [7, 13]. In the* Glce* knockout mice relatively few genes were differentially expressed, suggesting that skin is relatively unaffected by the lack of* Glce* in line with the observation that skin in these mice is phenotypically normal [20]. The skin phenotype of the Hpse overexpression mouse shows accelerated hair growth [7]. Gene expression analysis of this model showed only one differentially expressed gene (aggrecan). These results may touch upon the regulation of translation of mRNAs coding for GAG related enzymes. Enzymes involved in the synthesis and modification of GAGs as well genes coding for (some) growth factors share a common alternative translation mechanism via IRES sites [45, 46]. In general mRNAs are translated by the ribosomal scanning mechanism which scans for short leader sequences of 50 to 70 nucleotides [46, 47]. The leader sequences of the HS modifying enzymes and growth factors are characterized by long but structured sequences, which are not recognized by the ribosomal scanning mechanism [46, 47]. Within these sequences internal ribosomal entry sites (IRES) allow alternative translation, e.g., under stress conditions [47]. This indicates that in addition to mRNA levels an additional control mechanism on the translational level may be present. In addition, other types of regulatory levels are known including the interaction of biosynthetic enzymes with each other and the (possible) presence of large biosynthetic complexes (GAGosomes) [48]. This makes the regulation of GAG biosynthesis very complex, gene expression being only a part of it.

Taken together, it is concluded that a highly dynamic expression of genes involved in GAG metabolism and in GAG binding growth factors is associated with skin development. This indicates the importance of fine tuning of GAG structures during developmental processes. Further studies should focus on the biochemical analysis of the GAGs chains themselves.

## Figures and Tables

**Table 1:** Comparison of the number of differentially expressed genes during skin development in mice (p<0.10, fold>2.0) based on real-time qPCR and on exon array analysis.

**Total genes**	**System**		**E14 vs. P90**			**E16 vs. P90**			**P1 vs. P90**	
			*Down*	*Up*		*Down*	*Up*		*Down*	*Up*
***Production of precursors***										
43	TLDA		1	4		3	2		1	2
43	Exon		2	6		2	0		1	0
	Overlap		0	2		2	0		1	0
***Core proteins***										
14	TLDA		2	3		1	1		2	2
14	Exon		3	2		1	1		0	2
	Overlap		2	2		0	1		0	2
***Preparation of linkage region***										
8	TLDA		0	1		0	1		0	1
8	Exon		0	2		0	0		0	0
	Overlap		0	0		0	0		0	0
***Glycosaminoglycan chain polymerisation***										
13	TLDA		1	4		1	2		0	2
13	Exon		1	2		0	0		0	0
	Overlap		0	0		0	0		0	0
***Glycosaminoglycan chain modification***										
32	TLDA		1	9		0	5		0	8
32	Exon		1	3		0	3		0	1
	Overlap		1	2		0	1		0	0
***Glycosaminoglycan chain degradation***										
19	TLDA		2	2		3	1		0	1
19	Exon		3	1		1	1		2	0
	Overlap		2	1		1	1		0	0
***Growth factors***										
37	TLDA		0	13		3	8		1	11
37	Exon		2	14		3	10		1	4
	Overlap		0	10		2	8		0	4

*∗* P values for the exon array measurements were calculated using Benjamini–Hochberg multitesting correction. P values for the TLDA assay were calculated using an unpaired T-test.

Overlap refers to genes differentially expressed in both TLDA card and exon array.

**Table 2:** Differentially expressed GAG related genes during skin development in mice in comparison to mature skin (p<0.10) based on real-time qPCR.

			**E14 vs. P90**			**E16 vs. P90**			**P1 vs. P90**	
**Gene symbol**	**Full gene name and probe set**		**P-value**	**Relative change**		**P-value**	**Relative change**		**P-value**	**Relative change**
***Production of precursors***										
*Galk1 ‡*	Galactokinase 1		***0.029***	***5.897***		***0.057***	***3.867***		***0.074***	***3.043***
	Mm00444182_m1									
*Galt ‡*	Gal-1-P-Uridylyltransferase		*0.042*	*0.573*		***0.027***	***0.497***		0.832	0.964
	Mm00489459_g1									
*Gfpt1 ‡*	Glu-Fru-6-P-Transaminase 1		***0.002***	***2.378***		0.160	1.273		0.282	1.481
	Mm00600127_m1									
*Gfpt2 ‡*	Glu-Fru-6-P-Transaminase 2		0.269	0.772		***0.079***	***0.494***		0.356	0.780
	Mm00496565_m1									
*Hk2 ‡*	Hexokinase 2		*0.027*	*0.652*		0.253	1.390		*0.586*	*0.904*
	Mm00443385_m1									
*Pgm3 ‡*	Phosphoglucomutase 2		*0.058*	*1.876*		0.296	1.249		*0.098*	*1.470*
	Mm00459270_m1									
*Pgm5 ‡*	Phosphoglucomutase 5		***0.002***	***4.053***		***0.033***	***2.792***		***0.019***	***2.515***
	Mm00723432_m1									
*Slc13a5 ‡*	Solute Carrier Family 13 Member A5		Not detected			Not detected			Not detected	
	Mm00523288_m1									
*Slc26a9 ‡ *	Solute Carrier Family 26 Member A9		Not detected			0.036	0.264		0.023	0.318
	Mm00628490_m1									
*Slc35a3 ‡*	Solute Carrier Family 35 Member a3		***0.008***	***0.444***		*0.060*	*0.567*		0.455	0.811
	Mm00523288_m1									

***Core proteins***										
*Cd44*	CD44 Molecule		*0.018*	*0.536*		0.171	0.637		0.223	1.385
	Mm01277164_m1									
*Gpc2 ‡*	Glypican 2		***0.005***	***11.686***		0.638	1.294		0.369	1.249
	Mm00549650_m1									
*Gpc3 †*	Glypican 3		***<0.001***	***6.765***		***0.019***	***3.734***		***0.013***	***8.044***
	Mm00516722_m1									
*Gpc6 ‡*	Glypican 6		***0.027***	***2.880***		0.275	1.376		0.130	1.926
	Mm00516235_m1									
*Hspg2 ‡*	Perlecan		*0.030*	*1.504*		*0.064*	*1.959*		***0.007***	***3.325***
	Mm01181179_g1									
*Sdc1 ‡*	Syndecan 1		***0.012***	***0.293***		0.107	0.313		***0.069***	***0.403***
	Mm00448918_m1									
*Sdc4 †*	Syndecan 4		***0.002***	***0.201***		***0.039***	***0.240***		***0.065***	***0.471***
	Mm00488527_m1									

***Preparation of linkage region***										
*B3gat1*	*β*-1,3-Glucuronyltransferase 1		Not detected			Not detected			Not detected	
	Mm00661499_m1									
*B3gat2*	*β*-1,3-Glucuronyltransferase 2		Not detected			Not detected			Not detected	
	Mm00549042_m1									
*B4galt2 ‡*	*β*-1,4-Galactosyltransferase 2		***0.023***	***4.562***		***0.059***	***2.679***		***0.038***	***3.384***
	Mm00479556_m1									

***Glycosaminoglycan chain polymerisation***										
*Chpf ‡*	Chondroitin Polymerizing Factor		***<0.001***	***2.991***		*0.059*	*1.865*		***0.050***	***2.401***
	Mm01262239_g1									
*Chsy1 ‡*	CS Synthase 1		***0.013***	***3.900***		***0.024***	***2.282***		***0.072***	***2.229***
	Mm01319178_m1									
*Chsy3 ‡*	CS Synthase 3		***0.026***	***4.006***		***0.075***	***2.184***		0.101	2.013
	Mm01545329_m1									
*Csgalnact1 ‡*	CS-GalNAc-transferase 1		***0.099***	***0.496***		***0.098***	***0.475***		0.634	1.128
	Mm00555164_m1									
*Extl1 ‡*	Exostoses (multiple)-like 1		Not detected			Not detected			Not detected	
	Mm00621977_s1									
*Extl2 ‡*	Exostoses (multiple)-like 2		***0.007***	***2.043***		0.660	1.220		0.106	1.765
	Mm00469621_m1									
*Has2 ‡*	Hyaluronan Synthase 2		0.188	1.933		0.235	1.705		0.410	1.577
	Mm00515089_m1									

***Glycosaminoglycan chain modification***										
*Chst11 ‡*	Chondroitin 4-O-Sulfotransferase 1		***0.002***	***3.000***		*0.087*	*1.195*		*0.087*	*1.957*
	Mm00517563_m1									
*Chst14 ‡*	Dermatan 4 Sulfotransferase 1		***0.026***	***2.459***		0.156	1.513		0.203	1.707
	Mm00511291_s1									
*Chst2 ‡*	Carbohydrate Sulfotransferase 2		***0.014***	***3.664***		***0.010***	***3.145***		***0.006***	***2.773***
	Mm00490018_g1									
*Chst3 ‡*	Chondroitin 6-O-Sulfotransferase 1		***0.041***	***3.241***		0.152	1.941		***0.028***	***3.550***
	Mm00489736_m1									
*Chst8 ‡*	GalNAc-4-O-Sulfotransferase 1		***0.089***	***2.587***		0.221	0.591		0.139	2.280
	Mm00558321_m1									
*Hs3st1 ‡*	HS 3-O-sulfotransferase		*0.051*	*1.796*		*0.039*	*1.937*		***0.027***	***2.809***
	Mm01964038_m1									
*Hs3st3b1‡*	HS 3-O-sulfotransferase 3b1		***0.004***	***3.204***		***0.028***	***2.511***		***0.002***	***2.629***
	Mm00479621_m1									
*Hs3st6 ‡*	HS 3-O-sulfotransferase 6		***0.006***	***0.208***		*0.089*	*0.664*		*0.041*	*1.765*
	Mm01299930_m1									
*Hs6st2*	HS 6-O-sulfotransferase 2		Not detected			Not detected			Not detected	
	Mm00479296_m1									
*Ndst1 ‡*	N-deacet./N-sulfotrans. 1		0.118	1.487		0.140	1.449		***0.054***	***2.202***
	Mm00447005_m1									
*Ndst2 ‡*	N-deacet./N-sulfotrans. 2		*0.008*	*1.347*		***0.001***	***2.017***		***0.002***	***2.021***
	Mm00447818_m1									
*Ndst3 ‡*	N-deacet./N-sulfotrans. 3		***0.004***	***4.708***		***0.041***	***7.910***		***0.004***	***12.034***
	Mm00453178_m1									
*Sulf1 ‡*	Sulfatase 1		***0.004***	***4.644***		***0.079***	***2.674***		***0.089***	***2.077***
	Mm00552283_m1									
*Sumf2 ‡*	Sulfatase modifying factor 2		***0.008***	***2.657***		0.104	2.023		*0.038*	*1.857*
	Mm01197721_m1									

***Glycosaminoglycan chain degradation***										
*ArsJ ‡*	Arylsulfatase J		***0.013***	***7.805***		***0.010***	***12.075***		***0.014***	***7.146***
	Mm00557970_m1									
*ArsK ‡*	Arylsulfatase K		0.306	0.801		***0.059***	***0.466***		0.143	0.678
	Mm00513099_m1									
*Galns ‡*	Galactosamine (N-Acetyl)-6-Sulfatase		***<0.001***	***2.648***		*0.066*	*1.674*		*0.091*	*1.584*
	Mm00489575_m1									
*Hpse ‡*	Heparanase		***0.044***	***0.304***		0.342	1.450		0.169	0.578
	Mm00461768_m1									
*Hyal1 ‡*	Hyaluronoglucosamini-dase 1		***0.001***	***0.198***		***0.008***	***0.288***		*0.006*	*0.607*
	Mm00476206_m1									
*Sgsh ‡*	N-Sulfoglucosamine Sulfohydrolase		*0.055*	*0.647*		***0.002***	***0.435***		0.644	0.897
	Mm00450747_m1									

***Growth factors***										
*Areg*	Amphiregulin		Not detected			Not detected			0.656	0.834
	Mm00437583_m1									
*Bmp3 ‡*	Bone morphogenetic growth factor 3		***0.007***	***4.270***		***0.004***	***7.240***		***0.002***	***11.628***
	Mm00557790_m1									
*Bmp5 ‡*	Bone morphogenetic growth factor 5		***0.022***	***12.051***		0.303	1.788		0.587	1.300
	Mm00432091_m1									
*Ctgf ‡*	Connective tissue growth factor		0.668	1.079		***0.011***	***0.232***		*0.076*	*0.580*
	Mm01192931_g1									
*Fgf10 ‡*	Fibroblast growth factor 10		*0.063*	*1.649*		*0.050*	*1.801*		***0.093***	***2.262***
	Mm00433275_m1									
*Fgf13 ‡*	Fibroblast growth factor 13		***0.002***	***3.205***		*0.059*	*1.750*		0.181	1.544
	Mm00438910_m1									
*Fgf2 ‡*	Fibroblast growth factor 2		0.277	0.651		0.166	0.539		0.382	1.446
	Mm01285715_m1									
*Fgf20*	Fibroblast growth factor 20		Not detected			Not detected			Not detected	
	Mm00748347_m1									
*Fgf22 ‡*	Fibroblast growth factor 22		Not detected			0.386	0.632		*0.060*	*0.614*
	Mm00445749_m1									
*Fgf7 ‡*	Fibroblast growth factor 7		*0.045*	*0.606*		***0.002***	***0.394***		*0.087*	*0.630*
	Mm00433291_m1									
*Fgf8*	Fibroblast growth factor 8		Not detected			Not detected			Not detected	
	Mm00438921_m1									
*Figf ‡*	C-fos induced growth factor		*0.081*	*1.397*		*0.030*	*1.787*		0.545	0.834
	Mm01131929_m1									
*Gdf10 ‡*	Growth differentaition factor 10		***0.015***	***3.181***		0.454	1.143		0.166	1.519
	Mm03024279_s1									
*Hbegf ‡*	Heparin-binding epidermal growth factor		Not detected			***0.015***	***0.347***		***0.016***	***0.423***
	Mm00439305_g1									
*Hdgf †*	Hepatoma-derived growth factor		0.257	1.221		0.737	0.911		0.975	1.008
	Mm00725733_s1									
*Igf1 ‡*	Insulin-like growth factor 1		0.320	1.207		0.217	0.705		0.364	0.790
	Mm00439560_m1									
*Igf2 †*	Insulin-like growth factor 2		***<0.001***	***592.335***		***0.002***	***338.094***		***0.001***	***416.096***
	Mm00439565_g1									
*Nog ‡*	Noggin		***0.054***	***2.945***		***0.019***	***2.935***		***0.021***	***3.067***
	Mm01297833_s1									
*Pdgfa ‡*	Platelet-derived growth factor a		***0.021***	***3.005***		Not detected			***0.016***	***3.669***
	Mm01205760_m1									
*Pdgfb ‡*	Platelet-derived growth factor b		0.321	1.098		*0.033*	*1.468*		***0.010***	***2.096***
	Mm01298578_m1									
*Pdgfc ‡*	Platelet-derived growth factor c		Not detected			***0.016***	***2.362***		Not detected	
	Mm00480205_m1									
*Pdgfd ‡*	Platelet-derived growth factor d		Not detected			0.288	0.709		0.139	1.644
	Mm00546829_m1									
*Shh*	Sonic hedgehog		Not detected			Not detected			Not detected	
	Mm00436527_m1									
*Tgfb1 ‡*	Transforming growth factor beta 1		*0.027*	*0.540*		0.488	0.817		0.157	1.372
	Mm01178820_m1									
*Tgfb2 ‡*	Transforming growth factor beta 2		***0.039***	***2.697***		0.757	1.081		0.127	1.809
	Mm01321739_m1									
*Tgfb3 ‡*	Transforming growth factor beta 3		***0.033***	***2.420***		*0.094*	*1.854*		***0.034***	***2.517***
	Mm01307950_m1									
*Vegfa ‡*	Vascular endothelial growth factor a		0.394	1.108		0.112	1.613		0.461	1.358
	Mm01281447_m1									
*Vegfb *	Vascular endothelial growth factor b		Not detected			Not detected			Not detected	
	Mm00442102_m1									
*Vegfc ‡*	Vascular endothelial growth factor c		*0.024*	*1.839*		0.303	1.250		*0.015*	*1.996*
	Mm00437313_m1									
*Wnt10b ‡*	Wingless-related integration site 10b		0.180	5.829		0.122	9.688		0.105	11.748
	Mm00442104_m1									
*Wnt16 ‡*	Wingless-related integration site 16		***0.066***	***2.094***		***0.016***	***4.809***		***0.014***	***4.362***
	Mm00446420_m1									
*Wnt2 ‡*	Wingless-related integration site 2		***0.054***	***3.144***		*0.090*	*3.555*		*0.074*	*3.760*
	Mm00470018_m1									
*Wnt2b*	Wingless-related integration site 2b		Not detected			Not detected			Not detected	
	Mm00437330_m1									
*Wnt3a ‡*	Wingless-related integration site 3a		0.394	1.610		0.441	1.520		0.840	1.106
	Mm00437337_m1									
*Wnt6 ‡*	Wingless-related integration site 6		***0.015***	***11.709***		***0.018***	***10.400***		***0.016***	***10.758***
	Mm00437353_m1									
*Wnt7a *	Wingless-related integration site 7a		Not detected			Not detected			Not detected	
	Mm00437355_m1									
*Wnt7b ‡*	Wingless-related integration site 7b		***0.003***	***4.180***		***0.056***	***6.076***		***0.003***	***4.181***
	Mm00437357_m1									

Numbers in italic are significant (p<0.10); numbers in bold are >2-fold differentially expressed. Gene symbols indicated with a †-symbol are normalized using GAPDH as a reference gene. Gene symbols indicated with a ‡-symbol are normalized using TBP as a reference gene. Genes, for which a signal was not or only partly detected at a given time point or multiple time points and therefore a fold change and/or p value could not be calculated based on the available data, are given as “not detected.” Gene symbols for which all time points were classified as “not detected” do not show a symbol for the used reference gene due to lack of data for a calculation.

**Table 3:** Differentially expressed GAG related genes during skin development in mice in comparison to mature skin (p<0.10) based on gene Chip Mouse Exon 1.0 ST Arrays.

			**E14 vs. P90**			**E16 vs. P90**			**P1 vs. P90**	
**Gene symbol**	**Full gene name and probe set**		**Stepup P-value**	**Fold change**		**Stepup P-value**	**Fold change**		**Stepup P-value**	**Fold change**
***Production of precursors***										
*Galk1 *	Galactokinase 1		***0.030***	***4.242***		0.126	2.483		0.231	2.194
	6792485									
*Galt *	Gal-1-P-Uridylyltransferase		0.664	0.814		0.231	1.864		0.665	1.340
	6912944									
*Gfpt1 *	Glu-Fru-6-P-Transaminase 1		***0.020***	***2.110***		0.077	1.682		0.801	1.081
	6947679									
*Gfpt2 *	Glu-Fru-6-P-Transaminase 2		0.176	0.610		***0.087***	***0.426***		0.229	0.547
	6780767									
*Hk2*	Hexokinase 2		***0.003***	***0.451***		*0.040*	*1.375*		*0.039*	*0.655*
	6954982									
*Pgm3 *	Phosphoglucomutase 2		***0.083***	***2.193***		0.676	1.178		*0.654*	*1.286*
	6997513									
*Pgm5 *	Phosphoglucomutase 5		***0.058***	***2.338***		***0.104***	***2.209***		***0.291***	***1.696***
	6872290									
*Slc13a5*	Solute Carrier Family 13 Member A5		***0.003***	***2.588***		*0.059*	*1.322*		0.576	1.075
	6789531									
*Slc26a9 *	Solute Carrier Family 26 Member A9		**0.012**	**0.227**		***0.090***	***0.471***		***0.091***	***0.392***
	6753079									
*Slc35a3*	Solute Carrier Family 35 Member A3		0.105	0.767		*0.596*	*0.925*		0.572	0.890
	6908510									

***Core proteins***										
*Cd44*	CD44 Molecule		***0.009***	***0.370***		0.144	0.728		0.873	0.955
	6889258									
*Gpc2 *	Glypican 2		Not measured			Not measured			Not measured	
*Gpc3 *	Glypican 3		***0.003***	***4.339***		***0.013***	***3.305***		***0.015***	***4.721***
	7016826									
*Gpc6 *	Glypican 6		***0.019***	***2.767***		0.109	1.747		0.193	1.640
	6821985									
*Hspg2*	Perlecan		0.309	1.183		*0.075*	*1.554*		***0.042***	***2.230***
	6917933									
*Sdc1 *	Syndecan 1		***0.031***	***0.380***		***0.067***	***0.413***		0.103	0.424
	6793226									
*Sdc4 *	Syndecan 4		***0.017***	***0.341***		*0.094*	*0.536*		0.215	0.621
	6892905									

***Preparation of linkage region***										
*B3gat1*	*β*-1,3-Glucuronyltransferase 1		***0.008***	***3.467***		0.256	1.302		0.828	0.929
	6987632									
*B3gat2*	*β*-1,3-Glucuronyltransferase 2		***0.009***	***3.241***		0.286	1.269		0.690	1.129
	6748174									
*B4galt2*	*β*-1,4-Galactosyltransferase 2		*0.039*	*1.726*		*0.053*	*1.856*		0.176	1.477
	6924869									

***Glycosaminoglycan chain polymerisation***										
*Chpf *	Chondroitin Polymerizing Factor		*0.071*	*1.679*		0.182	1.459		0.309	1.403
	6759816									
*Chsy1 *	CS Synthase 1		Not measured			Not measured			Not measured	
*Chsy3 *	CS Synthase 3		0.429	1.153		*0.056*	*1.769*		0.849	1.058
	6861281									
*Cs-galnact1 *	CS-GalNAc-transferase 1		0.137	0.512		0.202	0.541		0.974	0.975
	6983073									
*Extl1 *	Exostoses (multiple)-like 1		***0.025***	***0.407***		0.149	0.625		0.144	0.505
	6926017									
*Extl2 *	Exostoses (multiple)-like 2		*0.085*	*1.702*		0.819	1.066		0.518	1.272
	6900659									
*Has2 *	Hyaluronan Synthase 2		***0.095***	***2.107***		0.157	1.969		0.537	1.403
	6854042									

***Glycosaminoglycan chain modification***										
*Chst11*	Chondroitin 4-O-Sulfotransferase 1		*0.049*	*1.922*		0.465	1.208		*0.239*	*1.531*
	6769366									
*Chst14*	Dermatan 4 Sulfotransferase 1		0.151	1.491		0.506	1.191		0.773	1.122
	6880476									
*Chst2 *	Carbohydrate Sulfotransferase 2		***0.035***	***2.302***		***0.072***	***2.160***		0.207	1.682
	6997990									
*Chst3 *	Chondroitin 6-O-Sulfotransferase 1		0.885	1.034		0.108	1.545		0.162	1.530
	6774295									
*Chst8 *	GalNAc-4-O-Sulfotransferase 1		0.945	1.007		0.569	1.054		0.420	1.108
	6966453									
*Hs3st1 *	HS 3-O-sulfotransferase 1		*0.074*	*1.658*		0.105	1.688		0.119	1.824
	6937654									
*Hs3st3b1*	HS 3-O-sulfotransferase 3b1		0.683	1.178		0.253	1.634		0.511	1.432
	6788991									
*Hs3st6*	HS 3-O-sulfotransferase 6		***0.019***	***0.406***		0.291	0.771		0.721	1.130
	6849317									
*Hs6st2*	HS 6O-sulfotransferase 2		***0.004***	***6.417***		***0.012***	***5.609***		***0.041***	***3.080***
	7016808									
*Ndst1 *	N-Deacetylase and N-Sulfotransferase 1		*0.090*	*1.363*		*0.056*	*1.649*		*0.067*	*1.763*
	6865926									
*Ndst2 *	N-Deacetylase and N-Sulfotransferase 2		0.822	1.044		*0.059*	*1.697*		*0.083*	*1.733*
	6823122									
*Ndst3 *	N-Deacetylase and N-Sulfotransferase 3		0.173	2.472		0.111	3.856		0.122	4.860
	6908958									
*Sulf1*	Sulfatase 1		***0.010***	***4.522***		***0.051***	***2.546***		0.232	1.627
	6747641									
*Sumf2*	Sulfatase modifying factor 2		Not measured			Not measured			Not measured	

***Glycosaminoglycan chain degradation***										
*ArsJ*	Arylsulfatase J		***0.003***	***2.498***		***0.006***	***3.164***		*0.031*	*1.702*
	6901136									
*ArsK *	Arylsulfatase K		0.368	0.784		0.102	0.543		0.514	0.779
	6814451									
*Galns *	Galactosamine (N-Acetyl)-6-Sulfatase		*0.087*	*1.671*		0.322	1.311		0.786	1.115
	6985943									
*Hpse*	Heparanase		***0.021***	***0.267***		0.540	1.244		***0.095***	***0.360***
	6940363									
*Hyal1*	Hyaluronoglucosamini-dase 1		***0.003***	***0.154***		***0.011***	***0.222***		***0.041***	***0.402***
	6992224									
*Sgsh *	N-Sulfoglucosamine Sulfohydrolase		***0.017***	***0.458***		*0.052*	*0.537*		0.201	0.699
	6792702									

***Growth factors***										
*Areg*	Amphiregulin		***0.016***	***0.157***		***0.043***	***0.200***		0.333	0.550
	6932394									
*Bmp3 *	Bone morphogenetic growth factor 3		0.280	1.392		***0.067***	***2.381***		0.100	2.365
	6932718									
*Bmp5 *	Bone Morphogenetic growth factor 5		***0.001***	***7.247***		0.141	1.191		0.688	1.057
	6990569									
*Ctgf *	Connective tissue growth factor		0.148	0.729		***0.025***	***0.366***		***0.068***	***0.485***
	6766623									
*Fgf10 *	Fibroblast growth factor 10		0.138	1.521		***0.057***	***2.250***		***0.094***	***2.142***
	6810592									
*Fgf13 *	Fibroblast growth factor 13		***0.012***	***2.998***		*0.075*	*1.824*		0.296	1.391
	7017134									
*Fgf2 *	Fibroblast growth factor 2		0.201	0.664		0.279	0.696		0.952	0.967
	6896850									
*Fgf20*	Fibroblast growth factor 20		0.659	1.228		0.125	2.483		0.777	1.220
	6981854									
*Fgf22 *	Fibroblast growth factor 22		***0.005***	***0.329***		0.972	0.994		*0.097*	*0.646*
	6769141									
*Fgf7 *	Fibroblast growth factor 7		0.531	0.740		***0.078***	***0.286***		0.212	0.418
	6880900									
*Fgf8*	Fibroblast growth factor 8		0.492	0.883		0.655	1.085		0.682	0.895
	6873363									
*Figf *	C-fos induced growth factor		*0.051*	*1.734*		*0.062*	*1.909*		0.548	0.832
	7015007									
*Gdf10 *	Growth differentiation factor 10		***0.028***	***2.334***		0.757	1.086		0.894	1.060
	6818153									
*Hbegf *	Heparin-binding epidermal growth factor		*0.037*	*0.551*		*0.063*	*0.547*		*0.087*	*0.530*
	6864680									
*Hdgf *	Hepatoma-derived growth factor		0.104	1.246		0.575	1.070		0.409	1.147
	6899028									
*Igf1 *	Insulin-like growth factor 1		0.235	0.631		0.173	0.537		0.398	0.641
	6769597									
*Igf2 *	Insulin-like growth factor 2		***0.001***	***59.615***		***0.002***	***55.864***		***0.002***	***52.364***
	6972317									
*Nog *	Noggin		0.275	2.605		0.517	1.773		0.677	1.712
	6790670									
*Pdgfa *	Platelet-derived growth factor a		***0.028***	***2.013***		***0.035***	***2.347***		***0.057***	***2.338***
	6942654									
*Pdgfb *	Platelet-derived growth factor b		*0.037*	*0.704*		0.199	1.197		0.157	1.298
	6837144									
*Pdgfc *	Platelet-derived growth factor c		***0.019***	***2.152***		***0.035***	***2.207***		0.902	1.042
	6898686									
*Pdgfd *	Platelet-derived growth factor d		0.925	0.952		0.191	0.504		0.790	1.205
	6986677									
*Shh*	Sonic hedgehog		0.448	0.595		0.308	2.117		0.152	4.571
	6936889									
*Tgfb1 *	Transforming growht factor beta 1		0.106	0.552		0.241	0.653		0.975	0.981
	6959236									
*Tgfb2 *	Transforming growht factor beta 2		***0.057***	***2.441***		0.448	1.335		0.264	1.802
	6764953									
*Tgfb3 *	Transforming growth factor beta 3		***0.026***	***2.386***		0.238	1.408		0.100	2.077
	6802449									
*Vegfa *	Vascular endothelial growth factor a		0.779	0.875		0.721	1.174		0.940	1.061
	6855659									
*Vegfb*	Vascular endothelial growth factor b		*0.008*	*1.461*		*0.031*	*1.318*		*0.060*	*1.277*
	6871273									
*Vegfc *	Vascular endothelial growth factor c		***0.088***	***2.120***		0.477	1.316		0.316	1.700
	6976200									
*Wnt10b *	Wingless-related integration site 10b		0.629	1.280		0.120	2.812		0.240	2.325
	6838399									
*Wnt16 *	Wingless-related integration site 16		0.358	1.180		***0.026***	***2.402***		*0.093*	*1.710*
	6944581									
*Wnt2 *	Wingless-related integration site 2		***0.057***	***3.047***		***0.082***	***3.260***		0.122	3.219
	6951974									
*Wnt2b*	Wingless-related integration site 2b		***0.009***	***2.189***		***0.024***	***2.030***		*0.077*	*1.590*
	6907887									
*Wnt3a *	Wingless-related integration site 3a		0.457	1.199		0.103	1.728		0.684	1.159
	6788662									
*Wnt6 *	Wingless-related integration site 6		***0.011***	***3.166***		***0.032***	***2.668***		***0.064***	***2.352***
	6750567									
*Wnt7a*	Wingless-related integration site 7a		***0.060***	***2.117***		0.131	1.865		0.395	1.456
	6955539									
*Wnt7b *	Wingless-related integration site 7b		***0.072***	***2.297***		***0.050***	***3.623***		0.237	1.912
	6837582									

Numbers in Italic are significant (p<0.10); numbers in bold are >2-fold differentially expressed. Genes indicated as “not measured” represent genes for which probes were not available on the used exon array version.

**Table 4:** Differentially expressed genes in C5 epimerase (Glce) knockout mouse (p<0.10) based on real-time qPCR.

**Gene symbol**	**Full gene name and probe set**		**P-value**	**Relative change**
***Production of precursors***				
*Gnpnat1*	Glucosamine-Phosphate N-Acetyltransferase 1		***0.033***	***0.468***
	Mm00834602_mH			
*Slc2a4*	Solute Carrier Family 2 Member 4		***0.086***	***2.526***
	Mm01245507_g1			
***Core proteins***				
*Acan*	Aggrecan		***0.005***	***0.242***
	Mm00545807_m1			
*Aspn*	Asporin		***0.010***	***0.382***
	Mm00445945_m1			
***Glycosaminoglycan chain polymerisation***				
*Csgalnact*	CS N-Acetylgalactosaminyltransferase 2		***0.049***	***0.431***
*2*	Mm00513340_m1			
***Glycosaminoglycan chain modification***				
*Glce*	Glucuronic Acid Epimerase		***0.013***	***0.079***
	Mm00473667_m1			

Numbers in Italic are significant (p<0.10); numbers in bold are >2 fold differentially expressed.

All genes were normalized using 18S RNA as a reference gene.

## Data Availability

The EXON array data used to support the findings of this study are included within the article and are provided via [18]. The Taqman low density array data used to support the findings of this study are included within the article. The data used to support the findings of this study are available from the corresponding author upon request.

## Supplementary Materials

Supplementary Materials The supplementary data contains 6 tables: Supplemental data Table 1: Design TLDA cart version 1: An overview of the used genes/assays on the Taqman Low Density Array, version 1. This table supports the materials and method section and the results section. Supplemental data Table 2: Design TLDA cart version 2: An overview of the used genes/assays on the Taqman Low Density Array, version 2. This table supports the materials and method section and the results section. Supplemental data Table 3: Deferentially expressed genes as % per category. This table provides a comparison between the EXON array expression data and the TLDA Data Supplemental data Table 4: Deferentially expressed genes during skin development in mice in comparison to mature mouse skin using TLDA cards. Supplemental data Table 5: Differentially expressed genes during skin development in mice in comparison to mature mouse skin using EXON array Supplementary data Table 6: Differentially expressed genes found using Taqman Low Density Arrays for the Glce knockout and HSPEtg mouse compared to wild type.
