# Supplementary material for: Dynamic Expression of Genes Involved in Proteoglycan/Glycosaminoglycan Metabolism during Skin Development
Source: Biomed Res Int. 2018 Aug 29;2018:9873471. doi: 10.1155/2018/9873471 (PMC6136507; doi:10.1155/2018/9873471)
Supplement: Supplementary Materials — The supplementary data contains 6 tables: Supplemental data Table 1: Design TLDA cart version 1: An overview of the used genes/assays on the Taqman Low Density Array, version 1. This table supports the materials and method section and the results section. Supplemental data Table 2: Design TLDA cart version 2: An overview of the used genes/assays on the Taqman Low Density Array, version 2. This table supports the materials and method section and the results section. Supplemental data Table 3: Deferentially expressed genes as % per category. This table provides a comparison between the EXON array expression data and the TLDA Data Supplemental data Table 4: Deferentially expressed genes during skin development in mice in comparison to mature mouse skin using TLDA cards. Supplemental data Table 5: Differentially expressed genes during skin development in mice in comparison to mature mouse skin using EXON array Supplementary data Table 6: Differentially expressed genes found using Taqman Low Density Arrays for the Glce knockout and HSPEtg mouse compared to wild type. [file 9873471.f1.pdf]

Supplementary data table 1: Design TLDA cart version 1

This design was used for the developmental study

Production of precursors

Taqman Assay ID	Gene symbol	Function
Mm00511302_m1	*Adpgk*	Phosphorylation of glucose into glucose-6-P
Mm00617772_g1	*Gale*	Conversion of UDP-GlcNAc to UDP-GalNAc
Mm00444182_m1	*Galk1*	Phosphorylation of Galactose into Galactose-1-P
Mm00613268_m1	*Galk2*	Phosphorylation of N-Acetylgalactosamine (GalNAc) into GalNAc-1P)
Mm00489459_g1	*Galt*	UDP-activation of Glucose-1-P and Galactose-1-P
Mm00439129_m1	*Gck*	Phosphorylation of glucose into glucose-6-P
Mm00600127_m1	*Gfpt1*	Conversion of fructose-6-P into glucosamine-6-P (Glc-6-P)
Mm00496565_m1	*Gfpt2*	Conversion of fructose-6-P into Glc-6-P
Mm00834602_mH	*Gnpnat1*	Conversion of Glc-6-P into N-Acetylglucosamine-6-P (GlcNAc-6-P)
Mm02026122_g1	*Gpi1*	Isomerisation of glucose-6-P into fructose-6-P
Mm00439344_m1	*Hk1*	Phosphorylation of glucose into glucose-6-P
Mm00443385_m1	*Hk2*	Phosphorylation of glucose into glucose-6-P
Mm00480214_m1	*Miox*	Conversion of Myo-inositol into UDP-GlcA
Mm00479878_m1	*Nagk*	Phosphorylation of GlcNAc to GlcNAC-6-P
Mm00442283_m1	*Papss1*	Synthesis of PAPS complex
Mm00442295_m1	*Papss2*	Synthesis of PAPS complex
Mm00804141_m1	*Pgm1*	Conversion of glucose-6-P into glucose-1-P
Mm00728285_s1	*Pgm2*	Conversion of glucose-6-P into glucose-1-P
Mm00459270_m1	*Pgm3*	Conversion of GlcNAc-6-P into GlcNAc-1-P
Mm00723432_m1	*Pgm5*	Conversion of glucose-6-P into glucose-1-P
Mm00490339_m1	*Slc13a1*	Transport of sulphate from the extracellular space to cytoplasm
Mm00556495_m1	*Slc13a4*	Transport of sulphate from the extracellular space to cytoplasm
Mm01334459_m1	*Slc13a5*	Transport of sulphate from the extracellular space to cytoplasm
Mm00626632_m1	*Slc26a11*	Transport of sulphate from the extracellular space to cytoplasm
Mm00432896_m1	*Slc26a2*	Transport of sulphate from the extracellular space to cytoplasm
Mm00506742_m1	*Slc26a6*	Transport of sulphate from the extracellular space to cytoplasm
Mm00524162_m1	*Slc26a7*	Transport of sulphate from the extracellular space to cytoplasm
Mm00524836_m1	*Slc26a8*	Transport of sulphate from the extracellular space to cytoplasm
Mm00628490_m1	*Slc26a9*	Transport of sulphate from the extracellular space to cytoplasm
Mm00446504_m1	*Slc35a2*	Transport from cytoplasm to Golgi system of UDP-GalNAc and UDP-Galactose
Mm00523288_m1	*Slc35a3*	Transport from cytoplasm to Golgi system of UDP-GlcNAc
Mm00782997_s1	*Slc35a4*	Transport from cytoplasm to Golgi system of UDP-GalNAc and UDP-Galactose
Mm00450258_m1	*Slc35b1*	Transport from cytoplasm to Golgi system of UDP-GalNAc and UDP-Galactose
Mm01269910_m1	*Slc35b2*	Transport of PAPS comples from cytoplasm to Golgi system
Mm00506275_m1	*Slc35b3*	Transport of PAPS comples from cytoplasm to Golgi system
Mm00480588_m1	*Slc35b4*	Transport from cytoplasm to Golgi system of UDP-GlcNAc and UDP-Xylose
Mm00624959_m1	*Slc35d1*	Transport from cytoplasm to Golgi system of UDP-GalNAc and UDP-GlcA
Mm01304830_m1	*Slc35d2*	Transport from cytoplasm to Golgi system of UDP-GlcNAc
Mm01717003_m1	*Slc35d3*	Paralog of Slc35d1
Mm00505476_m1	*Uap1*	UDP-activation of GlcNAc-1-P and GalNAc-1-P
Mm00447643_m1	*Ugdh*	Conversion of UDP-glucose into UDP-glucuronic acid (UDP-GlcA)
Mm00454826_m1	*Ugp2*	UDP-activation of Glucose-1-P
Mm00550735_m1	*Uxs1*	Conversion of UDP-GlcA into UDP-xylose

Core proteins

Taqman Assay ID	Gene symbol	Function
Mm01545840_m1	*Agrn*	Proteoglycan carring HS present in ECM
Mm01277164_m1	*Cd44*	Proteoglycan carring HS and hyaluronic acid on cell surface
Mm00487131_m1	*Col18a1*	Proteoglycan carring HS present in basement membrane
Mm00497305_m1	*Gpc1*	Proteoglycan carring HS present on cell surface via GPI-anchor
Mm00549650_m1	*Gpc2*	Proteoglycan carring HS present on cell surface via GPI-anchor
Mm00516722_m1	*Gpc3*	Proteoglycan carring HS present on cell surface via GPI-anchor
Mm00515035_m1	*Gpc4*	Proteoglycan carring HS present on cell surface via GPI-anchor
Mm00615599_m1	*Gpc5*	Proteoglycan carring HS present on cell surface via GPI-anchor
Mm00516235_m1	*Gpc6*	Proteoglycan carring HS present on cell surface via GPI-anchor
Mm01181179_g1	*Hspg2*	Proteoglycan carring HS present in ECM
Mm00448918_m1	*Sdc1*	Proteoglycan carring HS present on cell surface
Mm00484718_m1	*Sdc2*	Proteoglycan carring HS present on cell surface
Mm01179831_m1	*Sdc3*	Proteoglycan carring HS present on cell surface
Mm00488527_m1	*Sdc4*	Proteoglycan carring HS present on cell surface

Preparation of linkage region

Taqman Assay ID	Gene symbol	Function
Mm00504458_s1	*B3galt6*	Transferring UDP-galactose to galactose-xylose on proteoglycan
Mm00661499_m1	*B3gat1*	Transferring UDP-GlcA to galactose-galactose-xylose on proteoglycan
Mm00549042_m1	*B3gat2*	Transferring UDP-GlcA to galactose-galactose-xylose on proteoglycan
Mm00470389_m1	*B3gat3*	Transferring UDP-GlcA to galactose-galactose-xylose on proteoglycan
Mm00479556_m1	*B4galt2*	Transfering UDP-galactose to GlcNAc, Glc, and Xyl.
Mm00461357_m1	*B4galt7*	Transferring UDP-galactose to xylose on proteoglycan
Mm00558690_m1	*Xylt1*	Transferring UDP-xylose to proteoglycan
Mm00461181_m1	*Xylt2*	Transferring UDP-xylose to proteoglycan

Glycosaminoglycan chain polymerisation

Taqman Assay ID	Gene symbol	Function
Mm01262239_g1	*Chpf*	Transferring UDP-GlcA to GalNAc at end of sugar chain (CS)
Mm01319178_m1	*Chsy1*	Transferring UDP-GlcA to GalNAc at end of sugar chain (CS)
Mm01545329_m1	*Chsy3*	Transferring UDP-GlcA to GalNAc at end of sugar chain (CS)
Mm00555164_m1	*Csgalnact1*	Transferring UDP-GalNAc to GlcA at end of sugar chain (CS)
Mm00513340_m1	*Csgalnact2*	Transferring UDP-GalNAc to GlcA at end of sugar chain (CS)
Mm00468769_m1	*Ext1*	Transferring UDP-GlcNAc to GlcA at end of sugar chain (HS)
Mm00468775_m1	*Ext2*	Transferring UDP-GlcNAc to GlcA at end of sugar chain (HS)
Mm00621977_s1	*Extl1*	Transferring UDP-GlcA to GlcNAc at end of sugar chain (HS)
Mm00469621_m1	*Extl2*	Transferring UDP-GlcA to GlcNAc at end of sugar chain (HS)
Mm00516994_m1	*Extl3*	Transferring UDP-GlcA to GlcNAc at end of sugar chain (HS)
Mm00468496_m1	*Has1*	Polymerisation of hyaluronic acid
Mm00515089_m1	*Has2*	Polymerisation of hyaluronic acid
Mm00515091_m1	*Has3*	Polymerisation of hyaluronic acid

Glycosaminoglycan chain modification

Taqman Assay ID	Gene symbol	Function
Mm00517563_m1	*Chst11*	Sulfation at the 4-O postion of GalNAc untis (CS)
Mm00546416_s1	*Chst12*	Sulfation at the 4-O postion of GalNAc untis (CS)
Mm01186255_s1	*Chst13*	Sulfation at the 4-O postion of GalNAc untis (CS)
Mm00511291_s1	*Chst14*	Sulfation at the 4-O postion of GalNAc untis (CS)
Mm00513227_m1	*Chst15*	Sulfation at the 6-O postion of GalNAc untis (CS)
Mm00490018_g1	*Chst2*	Sulfation at the 6-O postion of GalNAc untis (CS)
Mm00489736_m1	*Chst3*	Sulfation at the 6-O postion of GalNAc untis (CS)
Mm00488783_s1	*Chst4*	Sulfation at the 6-O postion of GalNAc untis (CS)
Mm00517342_m1	*Chst5*	Sulfation at the 6-O postion of GalNAc untis (CS)
Mm00491466_m1	*Chst7*	Sulfation at the 6-O postion of GalNAc untis (CS)
Mm00558321_m1	*Chst8*	Sulfation at the 4-O postion of GalNAc untis (CS)
Mm01722279_m1	*Chst9*	Sulfation at the 4-O postion of GalNAc untis (CS)
Mm00552923_m1	*Dse*	Epimerisation of GlcA unit into iduronic acid unit (IdoA) (CS)
Mm00473667_m1	*Glce*	Epimerisation of GlcA unit into iduronic acid unit (IdoA) (HS)
Mm00478684_m1	*Hs2st1*	Sulfation at the 2-O position of GlcA and IdoA units (HS)
Mm01964038_s1	*Hs3st1*	Sulfation at the 3-O position of GlcNAc and GlcNS units sulfated at the 6-O position (HS)
Mm00616933_m1	*Hs3st2*	Sulfation at the 3-O position of GlcNAc and GlcNS units sulfated at the 6-O position (HS)
Mm00780907_s1	*Hs3st3a1*	Sulfation at the 3-O position of GlcNAc and GlcNS units sulfated at the 6-O position (HS)
Mm00479621_m1	*Hs3st3b1*	Sulfation at the 3-O position of GlcNAc and GlcNS units sulfated at the 6-O position (HS)
Mm01299930_m1	*Hs3st6*	Sulfation at the 3-O position of GlcNAc and GlcNS units sulfated at the 6-O position (HS)
Mm01229698_s1	*Hs6st1*	Sulfation at the 6-O position of GlcNAc and N-sulfated-glucuronic acid units (GlcNS) (HS)
Mm00479296_m1	*Hs6st2*	Sulfation at the 6-O position of GlcNAc and GlcNS units (HS)
Mm00479297_m1	*Hs6st3*	Sulfation at the 6-O position of GlcNAc and GlcNS units (HS)
Mm00447005_m1	*Ndst1*	N-de-acetylation and N-sulfation of GlcNAc unit (HS)
Mm00447818_m1	*Ndst2*	N-de-acetylation and N-sulfation of GlcNAc unit (HS)
Mm00453178_m1	*Ndst3*	N-de-acetylation and N-sulfation of GlcNAc unit (HS)
Mm00480767_m1	*Ndst4*	N-de-acetylation and N-sulfation of GlcNAc unit (HS)
Mm00552283_m1	*Sulf1*	Desulfation of the 6-O positon of 6-O sulfated GlcNAc and GlcNS units (HS)
Mm00511193_m1	*Sulf2*	Desulfation of the 6-O positon of 6-O sulfated GlcNAc and GlcNS units (HS)
Mm00461213_m1	*Sumf1*	Cofactor for Sulf1 and Sulf 2 (HS)
Mm01197721_m1	*Sumf2*	Cofactor for Sulf1 and Sulf 2 (HS)
Mm00616790_m1	*Ust*	Sulfation at the 2-O position of GlcA and IdoA units (CS)

Glycosaminoglycan chain degradation

Taqman Assay ID	Gene symbol	Function
Mm00802167_m1	*Arsb*	Desulfation of the 4-O position of 4-O sulfated GalNAc units (CS)
Mm00557970_m1	*Arsj*	Desulfation of the 4-O position of 4-O sulfated GalNAc units (CS)
Mm00513099_m1	*Arsk*	Desulfation of proteoglycans, paralog of IDS
Mm00489575_m1	*Galns*	Desulfation of the 6-O positon of 6-O sulfated GalNAc units (CS)
Mm00659592_m1	*Gns*	Desulfation of the 6-O positon of 6-O sulfated GlcNAc and GlcNS units (HS)
Mm00446954_g1	*Gusb*	Hydrolysation of GlcA from sugar chain (HS and Hyaluronic acid and CS)
Mm00599877_m1	*Hexa*	Hydrolysation of GalNAc units (CS) and GlcNAc untis (hyaluronic acid) from sugar chain
Mm00599880_m1	*Hexb*	Hydrolysation of GalNAc units (CS) and GlcNAc untis (hyaluronic acid) from sugar chain
Mm00519485_m1	*Hgsnat*	Acetylation of desulfated GlcNS units (HS)
Mm00461768_m1	*Hpse*	Degradation of HS chains extracellularly
Mm00476206_m1	*Hyal1*	Degradation of hyaluronic acid chain
Mm00477731_m1	*Hyal2*	Degradation of hyaluronic acid chain
Mm00662097_m1	*Hyal3*	Degradation of hyaluronic acid chain
Mm00494868_m1	*Ids*	Desulfation of 2-O sulfated IdoA and GlcA units (HS and CS)
Mm00515143_m1	*Idua*	Hydrolysation of IdoA unit from sugar chain (HS and CS)
Mm00452409_m1	*Mgea5*	Degradation of hyaluronic acid chain
Mm00476274_m1	*Naga*	Hydrolysis N-GalNAc moieties from glycoconjugates.
Mm00479175_m1	*Naglu*	Hydrolysation of GlcNAc from sugar chain (HS)
Mm00450747_m1	*Sgsh*	Desulfation of GlcNS units (HS)

Growth factors

Taqman Assay ID	Gene symbol	Function
Mm00437583_m1	*Areg*	Amphiregulin **
Mm00557790_m1	*Bmp3*	Bone morphogenetic growth factor 3 **
Mm00432091_m1	*Bmp5*	Bone morphogenetic growth factor 5 **
Mm01192931_g1	*Ctgf*	Connective tissue growth factor, embryonic growth factor in skin development **
Mm00433275_m1	*Fgf10*	Fibroblast growth factor 10 **
Mm00438910_m1	*Fgf13*	Fibroblast growth factor 13 **
Mm01285715_m1	*Fgf2*	Fibroblast growth factor 2 **
Mm00748347_m1	*Fgf20*	Fibroblast growth factor 20 **
Mm00445749_m1	*Fgf22*	Fibroblast growth factor 22 **
Mm00433291_m1	*Fgf7*	Fibroblast growth factor 7 **
Mm00438921_m1	*Fgf8*	Fibroblast growth factor 8 **
Mm01131929_m1	*Figf*	C-fos induced growth factor **
Mm03024279_s1	*Gdf10*	Growth differentiation factor 10 **
Mm00439305_g1	*Hbegf*	Heparin-binding epidermal growth factor **
Mm00725733_s1	*Hdgf*	Hepatoma-derived growth factor **
Mm00439560_m1	*Igf1*	Insulin-like growth factor 1 **
Mm00439565_g1	*Igf2*	Insulin-like growth factor 2 **
Mm01297833_s1	*Nog*	Noggin **
Mm01205760_m1	*Pdgfa*	Platelet-derived growth factor a **
Mm01298578_m1	*Pdgfb*	Platelet-derived growth factor b **
Mm00480205_m1	*Pdgfc*	Platelet-derived growth factor c **
Mm00546829_m1	*Pdgfd*	Platelet-derived growth factor d **
Mm00436527_m1	*Shh*	Sonic hedgehog **
Mm01178820_m1	*Tgfb1*	Transforming growth factor beta 1 **
Mm01321739_m1	*Tgfb2*	Transforming growth factor beta 2 **
Mm01307950_m1	*Tgfb3*	Transforming growth factor beta 3 **
Mm01281447_m1	*Vegfa*	Vascular endothelial growth factor a **
Mm00442102_m1	*Vegfb*	Vascular endothelial growth factor b **
Mm00437313_m1	*Vegfc*	Vascular endothelial growth factor c **
Mm00442104_m1	*Wnt10b*	Wingless-related integration site 10b **
Mm00446420_m1	*Wnt16*	Wingless-related integration site 16 **
Mm00470018_m1	*Wnt2*	Wingless-related integration site 2 **
Mm00437330_m1	*Wnt2b*	Wingless-related integration site 2b **
Mm00437337_m1	*Wnt3a*	Wingless-related integration site 3a **
Mm00437353_m1	*Wnt6*	Wingless-related integration site 6 **
Mm00437355_m1	*Wnt7a*	Wingless-related integration site 7a **
Mm00437357_m1	*Wnt7b*	Wingless-related integration site 7b **

Putatively involved

Taqman Assay ID	Gene symbol	Function
Hs99999901_s1	*18S*	Reference gene
Mm00802173_g1	*Arsa*	Desulfation of cerebroside
Mm00546931_m1	*Arsg*	Paralog of Arsa
Mm00480272_s1	*B3galnt1*	Transferring UDP-GalNAc or UDP-GlcNAc to N-GlcNAc, Galactose or N-GalNAc
Mm01189804_m1	*B4galnt3*	Transferring UDP-GalNAc or UDP-GlcNAc to N-GlcNAc, Galactose or N-GalNAc
Mm00625968_g1	*B4galnt4*	Transferring UDP-GalNAc or UDP-GlcNAc to N-GlcNAc, Galactose or N-GalNAc
Mm00473986_m1	*C1galt1*	Transferring UDP-Gal to N-GalNAc.
Mm00480676_m1	*C1galt1c1*	C1GALT1-specific chaperone 1
Mm00517855_m1	*Chst1*	Involved in sulfation of keratin sulfate
Mm03302249_g1	*Gapdh*	Reference gene
Mm00516323_m1	*Gla*	Hydrolysis of galactose units
Mm00515342_m1	*Glb1*	Hydrolysis of galactose units
Mm00464245_m1	*Glb1l2*	Hydrolysis of galactose units
Mm01254913_g1	*Gnpda1*	Conversion of GlcNAc-6-P into fructose-6-P
Mm00503573_m1	*Gnpda2*	Conversion of GlcNAc-6-P into fructose-6-P
Mm00434647_m1	*Khk*	Phosphorylating fructose to fructose-1-P
Mm00502002_m1	*Kl*	Putative glucuronidase
Mm00473122_m1	*Klb*	Putative glucuronidase
Mm00491720_m1	*Renbp*	Concersion of N-GlcNAc to N-acetylmannosamine
Mm00555344_m1	*Slc17a5*	Transport of GlcA from lysosoom to cytoplasm
Mm00445313_m1	*Slc26a3*	Transferring chloride ions across the cell membrane in exchange for bicarbonate ions
Mm00442308_m1	*Slc26a4*	Transferring chloride ions across the cell membrane
Mm00446145_m1	*Slc26a5*	Protein functions as a molecular motor in motile outer hair cells of the cochlea
Mm00442341_m1	*Slc35a1*	Putative CMP-Sialic Acid Transporter
Mm01277045_m1	*Tbp*	Reference gene
Mm00495930_m1	*Ugt8a*	Transfere galactose to ceramide

* Glc, glucosamine; GlcNAc, N-acetylated glucosamine; Gal, galactosamine; GalNAc, N-acetylated galactosamine; GlcA, glucuronic acid; IdoA, iduronic acid.

**Uijtdewilligen *et al* , 2016.

Supplementary data table 2: Design TLDA cart version 2

This design was used for analysis of skin from Glce knock out and HSPEtg mice

Production of precursors

Taqman Assay ID	Gene symbol	Function
Mm00511302_m1	*Adpgk*	Phosphorylation of glucose into glucose-6-P
Mm00617772_g1	*Gale*	Conversion of UDP-GlcNAc to UDP-GalNAc
Mm00444182_m1	*Galk1*	Phosphorylation of Galactose into Galactose-1-P
Mm00613268_m1	*Galk2*	Phosphorylation of N-Acetylgalactosamine (GalNAc) into GalNAc-1P)
Mm00489459_g1	*Galt*	UDP-activation of Glucose-1-P and Galactose-1-P
Mm00439129_m1	*Gck*	Phosphorylation of glucose into glucose-6-P
Mm00600127_m1	*Gfpt1*	Conversion of fructose-6-P into glucosamine-6-P (Glc-6-P)
Mm00496565_m1	*Gfpt2*	Conversion of fructose-6-P into Glc-6-P
Mm00834602_mH	*Gnpnat1*	Conversion of Glc-6-P into N-Acetylglucosamine-6-P (GlcNAc-6-P)
Mm02026122_g1	*Gpi1*	Isomerisation of glucose-6-P into fructose-6-P
Mm00439344_m1	*Hk1*	Phosphorylation of glucose into glucose-6-P
Mm00443385_m1	*Hk2*	Phosphorylation of glucose into glucose-6-P
Mm00480214_m1	*Miox*	Conversion of Myo-inositol into UDP-GlcA
Mm00479878_m1	*Nagk*	Phosphorylation of GlcNAc to GlcNAC-6-P
Mm00442283_m1	*Papss1*	Synthesis of PAPS complex
Mm00442295_m1	*Papss2*	Synthesis of PAPS complex
Mm00804141_m1	*Pgm1*	Conversion of glucose-6-P into glucose-1-P
Mm00728285_s1	*Pgm2*	Conversion of glucose-6-P into glucose-1-P
Mm00459270_m1	*Pgm3*	Conversion of GlcNAc-6-P into GlcNAc-1-P
Mm00723432_m1	*Pgm5*	Conversion of glucose-6-P into glucose-1-P
Mm00490339_m1	*Slc13a1*	Transport of sulphate from the extracellular space to cytoplasm
Mm00556495_m1	*Slc13a4*	Transport of sulphate from the extracellular space to cytoplasm
Mm01334459_m1	*Slc13a5*	Transport of sulphate from the extracellular space to cytoplasm
Mm00626632_m1	*Slc26a11*	Transport of sulphate from the extracellular space to cytoplasm
Mm00432896_m1	*Slc26a2*	Transport of sulphate from the extracellular space to cytoplasm
Mm00506742_m1	*Slc26a6*	Transport of sulphate from the extracellular space to cytoplasm
Mm00524162_m1	*Slc26a7*	Transport of sulphate from the extracellular space to cytoplasm
Mm00524836_m1	*Slc26a8*	Transport of sulphate from the extracellular space to cytoplasm
Mm00628490_m1	*Slc26a9*	Transport of sulphate from the extracellular space to cytoplasm
Mm01192270_m1	*Slc2a1 (new)*	Sugar transport across plasma membrane
Mm00453716_m1	*Slc2a10 (new)*	Sugar transport across plasma membrane
Mm00619244_m1	*Slc2a12 (new)*	Sugar transport across plasma membrane
Mm00446230_g1	*Slc2a2 (new)*	Sugar transport across plasma membrane
Mm00441483_m1	*Slc2a3 (new)*	Sugar transport across plasma membrane
Mm01245507_g1	*Slc2a4 (new)*	Sugar transport across plasma membrane
Mm00600311_m1	*Slc2a5 (new)*	Sugar transport across plasma membrane
Mm00554217_m1	*Slc2a6 (new)*	Sugar transport across plasma membrane
Mm00444634_m1	*Slc2a8 (new)*	Sugar transport across plasma membrane
Mm01211147_m1	*Slc2a9 (new)*	Sugar transport across plasma membrane
Mm00446504_m1	*Slc35a2*	Transport from cytoplasm to Golgi system of UDP-GalNAc and UDP-Galactose
Mm00523288_m1	*Slc35a3*	Transport from cytoplasm to Golgi system of UDP-GlcNAc
Mm00782997_s1	*Slc35a4*	Transport from cytoplasm to Golgi system of UDP-GalNAc and UDP-Galactose
Mm00450258_m1	*Slc35b1*	Transport from cytoplasm to Golgi system of UDP-GalNAc and UDP-Galactose
Mm01269910_m1	*Slc35b2*	Transport of PAPS comples from cytoplasm to Golgi system
Mm00506268_m1	*Slc35b3*	Transport of PAPS comples from cytoplasm to Golgi system
Mm00480588_m1	*Slc35b4*	Transport from cytoplasm to Golgi system of UDP-GlcNAc and UDP-Xylose
Mm00624959_m1	*Slc35d1*	Transport from cytoplasm to Golgi system of UDP-GalNAc and UDP-GlcA
Mm01304830_m1	*Slc35d2*	Transport from cytoplasm to Golgi system of UDP-GlcNAc
Mm01717003_m1	*Slc35d3*	Paralog of Slc35d1
Mm00769477_m1	*Uap1*	UDP-activation of GlcNAc-1-P and GalNAc-1-P
Mm00447643_m1	*Ugdh*	Conversion of UDP-glucose into UDP-glucuronic acid (UDP-GlcA)
Mm00454826_m1	*Ugp2*	UDP-activation of Glucose-1-P
Mm00550735_m1	*Uxs1*	Conversion of UDP-GlcA into UDP-xylose

Core proteins

Taqman Assay ID	Gene symbol	Function
Mm00545807_m1	*Acan (new)*	Proteoglycan carring CS or DS
Mm01264855_m1	*Agrn*	Proteoglycan carring HS present in ECM
Mm00445945_m1	*Aspn (new)*	Proteoglycan carring CS or DS
Mm00476090_m1	*Bcan (new)*	Proteoglycan carring CS or DS
Mm00455918_m1	*Bgn (new)*	Proteoglycan carring CS or DS
Mm01277164_m1	*Cd44*	Proteoglycan carring HS and hyaluronic acid on cell surface
Mm00487131_m1	*Col18a1*	Proteoglycan carring HS present in basement membrane
Mm00483872_m1	*Col9a2 (new)*	Proteoglycan carring CS or DS
Mm00507256_m1	*Cspg4 (new)*	Proteoglycan carring CS or DS
Mm00516549_m1	*Cspg5 (new)*	Proteoglycan carring CS or DS
Mm03003496_s1	*Dcn (new)*	Proteoglycan carring CS or DS
Mm00514611_m1	*Epyc (new)*	Proteoglycan carring CS or DS
Mm00497305_m1	*Gpc1*	Proteoglycan carring HS present on cell surface via GPI-anchor
Mm00549650_m1	*Gpc2*	Proteoglycan carring HS present on cell surface via GPI-anchor
Mm00516722_m1	*Gpc3*	Proteoglycan carring HS present on cell surface via GPI-anchor
Mm00515035_m1	*Gpc4*	Proteoglycan carring HS present on cell surface via GPI-anchor
Mm00615599_m1	*Gpc5*	Proteoglycan carring HS present on cell surface via GPI-anchor
Mm00516235_m1	*Gpc6*	Proteoglycan carring HS present on cell surface via GPI-anchor
Mm00469183_m1	*Hmmr (new)*	Hyaluronan-Mediated Motility Receptor
Mm01181165_m1	*Hspg2*	Proteoglycan carring HS present in ECM
Mm00484007_m1	*Ncan (new)*	Proteoglycan carring CS or DS
Mm00448918_m1	*Sdc1*	Proteoglycan carring HS present on cell surface
Mm00484718_m1	*Sdc2*	Proteoglycan carring HS present on cell surface
Mm01179831_m1	*Sdc3*	Proteoglycan carring HS present on cell surface
Mm00488527_m1	*Sdc4*	Proteoglycan carring HS present on cell surface
Mm00484015_m1	*Smc3 (new)*	Proteoglycan carring CS or DS
Mm01169070_m1	*Srgn (new)*	Proteoglycan carring CS or DS
Mm01283063_m1	*Vcan (new)*	Proteoglycan carring CS or DS

Preparation of linkage region

Taqman Assay ID	Gene symbol	Function
Mm00504458_s1	*B3galt6*	Transferring UDP-galactose to galactose-xylose on proteoglycan
Mm00661499_m1	*B3gat1*	Transferring UDP-GlcA to galactose-galactose-xylose on proteoglycan
Mm00549042_m1	*B3gat2*	Transferring UDP-GlcA to galactose-galactose-xylose on proteoglycan
Mm00470389_m1	*B3gat3*	Transferring UDP-GlcA to galactose-galactose-xylose on proteoglycan
Mm00479556_m1	*B4galt2*	Transfering UDP-galactose to GlcNAc, Glc, and Xyl.
Mm00461357_m1	*B4galt7*	Transferring UDP-galactose to xylose on proteoglycan
Mm00558690_m1	*Xylt1*	Transferring UDP-xylose to proteoglycan
Mm00461181_m1	*Xylt2*	Transferring UDP-xylose to proteoglycan

Glycosaminoglycan chain polymerisation

Taqman Assay ID	Gene symbol	Function
Mm01262239_g1	*Chpf*	Transferring UDP-GlcA to GalNAc at end of sugar chain (CS)
Mm01319178_m1	*Chsy1*	Transferring UDP-GlcA to GalNAc at end of sugar chain (CS)
Mm01545329_m1	*Chsy3*	Transferring UDP-GlcA to GalNAc at end of sugar chain (CS)
Mm00555164_m1	*Csgalnact1*	Transferring UDP-GalNAc to GlcA at end of sugar chain (CS)
Mm00513340_m1	*Csgalnact2*	Transferring UDP-GalNAc to GlcA at end of sugar chain (CS)
Mm00468769_m1	*Ext1*	Transferring UDP-GlcNAc to GlcA at end of sugar chain (HS)
Mm00468775_m1	*Ext2*	Transferring UDP-GlcNAc to GlcA at end of sugar chain (HS)
Mm00621977_s1	*Extl1*	Transferring UDP-GlcA to GlcNAc at end of sugar chain (HS)
Mm00469621_m1	*Extl2*	Transferring UDP-GlcA to GlcNAc at end of sugar chain (HS)
Mm00516994_m1	*Extl3*	Transferring UDP-GlcA to GlcNAc at end of sugar chain (HS)
Mm00468496_m1	*Has1*	Polymerisation of hyaluronic acid
Mm00515089_m1	*Has2*	Polymerisation of hyaluronic acid
Mm00515091_m1	*Has3*	Polymerisation of hyaluronic acid

Glycosaminoglycan chain modification

Taqman Assay ID	Gene symbol	Function
Mm00517563_m1	*Chst11*	Sulfation at the 4-O postion of GalNAc untis (CS)
Mm00546416_s1	*Chst12*	Sulfation at the 4-O postion of GalNAc untis (CS)
Mm01186255_s1	*Chst13*	Sulfation at the 4-O postion of GalNAc untis (CS)
Mm00511291_s1	*Chst14*	Sulfation at the 4-O postion of GalNAc untis (CS)
Mm00513227_m1	*Chst15*	Sulfation at the 6-O postion of GalNAc untis (CS)
Mm00490018_g1	*Chst2*	Sulfation at the 6-O postion of GalNAc untis (CS)
Mm00489736_m1	*Chst3*	Sulfation at the 6-O postion of GalNAc untis (CS)
Mm00517342_m1	*Chst5*	Sulfation at the 6-O postion of GalNAc untis (CS)
Mm00491466_m1	*Chst7*	Sulfation at the 6-O postion of GalNAc untis (CS)
Mm00558321_m1	*Chst8*	Sulfation at the 4-O postion of GalNAc untis (CS)
Mm01722279_m1	*Chst9*	Sulfation at the 4-O postion of GalNAc untis (CS)
Mm00552923_m1	*Dse*	Epimerisation of GlcA unit into iduronic acid unit (IdoA) (CS)
Mm00473667_m1	*Glce*	Epimerisation of GlcA unit into iduronic acid unit (IdoA) (HS)
Mm00478684_m1	*Hs2st1*	Sulfation at the 2-O position of GlcA and IdoA units (HS)
Mm01964038_s1	*Hs3st1*	Sulfation at the 3-O position of GlcNAc and GlcNS units sulfated at the 6-O position (HS)
Mm00616933_m1	*Hs3st2*	Sulfation at the 3-O position of GlcNAc and GlcNS units sulfated at the 6-O position (HS)
Mm00780907_s1	*Hs3st3a1*	Sulfation at the 3-O position of GlcNAc and GlcNS units sulfated at the 6-O position (HS)
Mm00479621_m1	*Hs3st3b1*	Sulfation at the 3-O position of GlcNAc and GlcNS units sulfated at the 6-O position (HS)
Mm01192940_m1	*Hs3st5 (new)*	Sulfation at the 3-O position of GlcNAc and GlcNS units sulfated at the 6-O position (HS)
Mm01299930_m1	*Hs3st6*	Sulfation at the 3-O position of GlcNAc and GlcNS units sulfated at the 6-O position (HS)
Mm01229698_s1	*Hs6st1*	Sulfation at the 6-O position of GlcNAc and N-sulfated-glucuronic acid units (GlcNS) (HS)
Mm00479296_m1	*Hs6st2*	Sulfation at the 6-O position of GlcNAc and GlcNS units (HS)
Mm00479297_m1	*Hs6st3*	Sulfation at the 6-O position of GlcNAc and GlcNS units (HS)
Mm00447005_m1	*Ndst1*	N-de-acetylation and N-sulfation of GlcNAc unit (HS)
Mm00447818_m1	*Ndst2*	N-de-acetylation and N-sulfation of GlcNAc unit (HS)
Mm00453178_m1	*Ndst3*	N-de-acetylation and N-sulfation of GlcNAc unit (HS)
Mm00480767_m1	*Ndst4*	N-de-acetylation and N-sulfation of GlcNAc unit (HS)
Mm00552283_m1	*Sulf1*	Desulfation of the 6-O positon of 6-O sulfated GlcNAc and GlcNS units (HS)
Mm01248026_m1	*Sulf2*	Desulfation of the 6-O positon of 6-O sulfated GlcNAc and GlcNS units (HS)
Mm00461213_m1	*Sumf1*	Cofactor for Sulf1 and Sulf 2 (HS)
Mm01197721_m1	*Sumf2*	Cofactor for Sulf1 and Sulf 2 (HS)
Mm00616790_m1	*Ust*	Sulfation at the 2-O position of GlcA and IdoA units (CS)

Glycosaminoglycan chain degradation

Taqman Assay ID	Gene symbol	Function
Mm00503655_s1	*Arsb*	Desulfation of the 4-O position of 4-O sulfated GalNAc units (CS)
Mm00557970_m1	*Arsj*	Desulfation of the 4-O position of 4-O sulfated GalNAc units (CS)
Mm00513099_m1	*Arsk*	Desulfation of proteoglycans, paralog of IDS
Mm00489576_m1	*Galns*	Desulfation of the 6-O positon of 6-O sulfated GalNAc units (CS)
Mm00659592_m1	*Gns*	Desulfation of the 6-O positon of 6-O sulfated GlcNAc and GlcNS units (HS)
Mm01197698_m1	*Gusb*	Hydrolysation of GlcA from sugar chain (HS and Hyaluronic acid and CS)
Mm00599877_m1	*Hexa*	Hydrolysation of GalNAc units (CS) and GlcNAc untis (hyaluronic acid) from sugar chain
Mm00599880_m1	*Hexb*	Hydrolysation of GalNAc units (CS) and GlcNAc untis (hyaluronic acid) from sugar chain
Mm00519485_m1	*Hgsnat*	Acetylation of desulfated GlcNS units (HS)
Mm00461768_m1	*Hpse*	Degradation of HS chains extracellularly
Mm00477731_m1	*Hyal2*	Degradation of hyaluronic acid chain
Mm00662097_m1	*Hyal3*	Degradation of hyaluronic acid chain
Mm00494868_m1	*Ids*	Desulfation of 2-O sulfated IdoA and GlcA units (HS and CS)
Mm00515143_m1	*Idua*	Hydrolysation of IdoA unit from sugar chain (HS and CS)
Mm00452409_m1	*Mgea5*	Degradation of hyaluronic acid chain
Mm00476274_m1	*Naga*	Hydrolysis N-GalNAc moieties from glycoconjugates.
Mm00479175_m1	*Naglu*	Hydrolysation of GlcNAc from sugar chain (HS)
Mm00480053_m1	*Nat6;Hyal1*	Degradation of hyaluronic acid chain
Mm00450747_m1	*Sgsh*	Desulfation of GlcNS units (HS)

Growth factors

Taqman Assay ID	Gene symbol	Function
Mm01192933_g1	*Ctgf*	Connective tissue growth factor, embryonic growth factor in skin development **
Mm00433275_m1	*Fgf10*	Fibroblast growth factor 10 **
Mm01285715_m1	*Fgf2*	Fibroblast growth factor 2 **
Mm00438917_m1	*Fgf4 (new)*	Growth factor (Uijtdewilligen et al, 2013)**
Mm00433291_m1	*Fgf7*	Fibroblast growth factor 7 **
Mm00438921_m1	*Fgf8*	Fibroblast growth factor 8 **
Mm01131929_m1	*Figf*	C-fos induced growth factor **
Mm00439305_g1	*Hbegf*	Heparin-binding epidermal growth factor **
Mm01205760_m1	*Pdgfa*	Platelet-derived growth factor a **
Mm01298578_m1	*Pdgfb*	Platelet-derived growth factor b **
Mm03053266_s1	*Pdgfc*	Platelet-derived growth factor c **
Mm00546829_m1	*Pdgfd*	Platelet-derived growth factor d **
Mm00436527_m1	*Shh*	Sonic hedgehog **
Mm01178819_m1	*Tgfb1*	Transforming growth factor beta 1 **
Mm01321739_m1	*Tgfb2*	Transforming growth factor beta 2 **
Mm01307950_m1	*Tgfb3*	Transforming growth factor beta 3 **
Mm01281449_m1	*Vegfa*	Vascular endothelial growth factor a **
Mm00442102_m1	*Vegfb*	Vascular endothelial growth factor b **
Mm01202432_m1	*Vegfc*	Vascular endothelial growth factor c **

Putatively involved

Taqman Assay ID	Gene symbol	Function
Hs99999901_s1	*18S*	Reference gene
Mm00607939_s1	*Actb (new)*	Reference gene
Mm00802173_g1	*Arsa*	Desulfation of cerebroside
Mm00546931_m1	*Arsg*	Paralog of Arsa
Mm00473986_m1	*C1galt1*	Transferring UDP-Gal to N-GalNAc.
Mm00480676_m1	*C1galt1c1*	C1GALT1-specific chaperone 1
Mm00517855_m1	*Chst1*	Involved in sulfation of keratin sulfate
Mm03302249_g1	*Gapdh*	Reference gene
Mm00516323_m1	*Gla*	Hydrolysis of galactose units
Mm00515342_m1	*Glb1*	Hydrolysis of galactose units
Mm00464245_m1	*Glb1l2*	Hydrolysis of galactose units
Mm01254913_g1	*Gnpda1;Gm8615*	Conversion of GlcNAc-6-P into fructose-6-P
Mm00503573_m1	*Gnpda2*	Conversion of GlcNAc-6-P into fructose-6-P
Mm01324427_m1	*Hprt1 (new)*	Reference gene
Mm00502002_m1	*Kl*	Putative glucuronidase
Mm00473122_m1	*Klb*	Putative glucuronidase
Mm00555344_m1	*Slc17a5*	Transport of GlcA from lysosoom to cytoplasm
Mm00442341_m1	*Slc35a1*	Putative CMP-Sialic Acid Transporter
Mm01277042_m1	*Tbp*	Reference gene
Mm00495930_m1	*Ugt8a*	Transfere galactose to ceramide

Gene symbols marked with new are added in the second version of the TLDA cart.

* Glc, glucosamine; GlcNAc, N-acetylated glucosamine; Gal, galactosamine; GalNAc, N-acetylated galactosamine; GlcA, glucuronic acid; IdoA, iduronic acid.

**Uijtdewilligen et al, 2016.

Supplementary data table 3: Differentially expressed genes as % per category

Catagory	Total genes	Not measured* / Not detected **	E14vsP90		E16vsP90		P1vsP90	
			Differentially expressed	*Percentage*	*Differentially expressed*	*Percentage*	*Differentially expressed*	*Percentage*
Preparation of percursors	43	8	15	35%	6	14%	8	19%
Core proteins	14	1	11	79%	4	29%	5	36%
Preparation of linkage region	8	2	3	38%	2	25%	3	38%
Sugar chain polymerisation	13	3	7	54%	5	38%	5	38%
Sugar chain modification	32	9	14	44%	10	31%	13	41%
Sugar chain degradation	19	1	10	53%	9	47%	5	26%
Growth factors	37	6	18	49%	16	43%	16	43%
Putativley involved	23	5	11	48%	10	43%	3	13%

* Some genes were not available on the used exon array version.

** Genes, for which a signal was not or only partly detected at a given timepoint or multiple timepoints and therefore a fold change and/or p-value could not be calculated based on the available data, are given as ‘not detected’. Gene symbol for which all timepoints were classified as ‘not detected’ do not show a reference gene due to lack of data for a calculation.

Supplementary data table 4: Differientally expressed genes during skin development in mice in comparison to mature mouse skin using TLDA cards

Production of precursors

Gene symbol	Taqman assay ID	Reference gene	E14vsP90 P-value	E14vsP90 Relative fold expression	E16vsP90 P-value	E16vsP90 Relative fold expression	P1vsP90 P-value	P1vsP90 Relative fold expression	Function
Adpgk	Mm00511302_m1	*Tbp*	0.915	1.033	0.316	1.410	0.144	1.620	Phosphorylation of glucose into glucose-6-P
Gale	Mm00617772_g1	*Tbp*	0.921	1.004	0.822	0.906	0.002	0.628	Conversion of UDP-GlcNAc to UDP-GalNAc
Galk1	Mm00444182_m1	*Tbp*	0.029	5.897	0.057	3.867	0.074	3.043	Phosphorylation of Galactose into Galactose-1-P
Galk2	Mm00613268_m1	*Tbp*	0.710	1.123	0.682	1.109	0.364	1.237	Phosphorylation of N-Acetylgalactosamine (GalNAc) into GalNAc-1P)
Galt	Mm00489459_g1	*Tbp*	0.042	0.573	0.027	0.497	0.832	0.964	UDP-activation of Glucose-1-P and Galactose-1-P
Gck	Mm00439129_m1		Not detected		Not detected		Not detected		Phosphorylation of glucose into glucose-6-P
Gfpt1	Mm00600127_m1	*Tbp*	0.002	2.378	0.160	1.273	0.282	1.481	Conversion of fructose-6-P into glucosamine-6-P (Glc-6-P)
Gfpt2	Mm00496565_m1	*Tbp*	0.269	0.772	0.079	0.494	0.356	0.780	Conversion of fructose-6-P into Glc-6-P
Gnpnat1	Mm00834602_mH	*Tbp*	0.306	0.890	0.221	0.652	0.369	0.900	Conversion of Glc-6-P into N-Acetylglucosamine-6-P (GlcNAc-6-P)
Gpi1	Mm02026122_g1	*Gapdh*	0.035	1.336	0.742	0.906	0.195	1.193	Isomerisation of glucose-6-P into fructose-6-P
Hk1	Mm00439344_m1	*Tbp*	0.196	1.163	0.598	0.837	0.447	0.741	Phosphorylation of glucose into glucose-6-P
Hk2	Mm00443385_m1	*Tbp*	0.027	0.652	0.253	1.390	0.586	0.904	Phosphorylation of glucose into glucose-6-P
Miox	Mm00480214_m1		Not detected		Not detected		Not detected		Conversion of Myo-inositol into UDP-GlcA
Nagk	Mm00479878_m1	*Tbp*	0.139	1.277	0.620	1.112	0.200	1.157	Phosphorylation of GlcNAc to GlcNAC-6-P
Papss1	Mm00442283_m1	*Tbp*	0.037	1.472	0.907	1.045	0.400	1.304	Synthesis of PAPS complex
Papss2	Mm00442295_m1	*Tbp*	0.015	1.993	0.383	1.380	0.283	1.453	Synthesis of PAPS complex
Pgm1	Mm00804141_m1	*Tbp*	0.100	0.582	0.194	0.702	0.185	0.717	Conversion of glucose-6-P into glucose-1-P
Pgm2	Mm00728285_s1	*Tbp*	0.238	0.721	0.940	1.022	0.475	1.161	Conversion of glucose-6-P into glucose-1-P
Pgm3	Mm00459270_m1	*Tbp*	0.058	1.876	0.296	1.249	0.098	1.470	Conversion of GlcNAc-6-P into GlcNAc-1-P
Pgm5	Mm00723432_m1	*Tbp*	0.002	4.053	0.033	2.792	0.019	2.515	Conversion of glucose-6-P into glucose-1-P
Slc13a1	Mm00490339_m1		Not detected		Not detected		Not detected		Transport of sulphate from the extracellular space to cytoplasm
Slc13a4	Mm00556495_m1	*Tbp*	0.007	11.752	Not detected		Not detected		Transport of sulphate from the extracellular space to cytoplasm
Slc13a5	Mm01334459_m1		Not detected		Not detected		Not detected		Transport of sulphate from the extracellular space to cytoplasm
Slc26a11	Mm00626632_m1	*Tbp*	0.190	0.860	0.513	1.160	0.028	1.905	Transport of sulphate from the extracellular space to cytoplasm
Slc26a2	Mm00432896_m1		Not detected		Not detected		Not detected		Transport of sulphate from the extracellular space to cytoplasm
Slc26a6	Mm00506742_m1	*Tbp*	0.412	1.221	0.291	0.656	0.733	1.082	Transport of sulphate from the extracellular space to cytoplasm
Slc26a7	Mm00524162_m1	*Tbp*	0.546	0.824	0.838	0.969	0.184	1.655	Transport of sulphate from the extracellular space to cytoplasm
Slc26a8	Mm00524836_m1		Not detected		Not detected		Not detected		Transport of sulphate from the extracellular space to cytoplasm
Slc26a9	Mm00628490_m1	*Tbp*	Not detected		0.036	0.264	0.023	0.318	Transport of sulphate from the extracellular space to cytoplasm
Slc35a2	Mm00446504_m1	*Tbp*	0.016	1.577	0.311	1.287	0.678	1.142	Transport from cytoplasm to Golgi system of UDP-GalNAc and UDP-Galactose
Slc35a3	Mm00523288_m1	*Tbp*	0.008	0.444	0.060	0.567	0.455	0.811	Transport from cytoplasm to Golgi system of UDP-GlcNAc
Slc35a4	Mm00782997_s1	*Tbp*	0.222	1.374	0.244	1.347	0.147	1.604	Transport from cytoplasm to Golgi system of UDP-GalNAc and UDP-Galactose
Slc35b1	Mm00450258_m1	*Tbp*	0.882	0.965	0.606	0.875	0.748	1.080	Transport from cytoplasm to Golgi system of UDP-GalNAc and UDP-Galactose
Slc35b2	Mm01269910_m1	*Tbp*	0.000	1.889	0.196	1.436	0.075	1.663	Transport of PAPS comples from cytoplasm to Golgi system
Slc35b3	Mm00506275_m1	*Tbp*	0.832	0.947	0.251	1.097	0.293	1.258	Transport of PAPS comples from cytoplasm to Golgi system
Slc35b4	Mm00480588_m1	*Tbp*	0.019	1.443	0.467	0.824	0.574	1.233	Transport from cytoplasm to Golgi system of UDP-GlcNAc and UDP-Xylose
Slc35d1	Mm00624959_m1	*Tbp*	0.186	1.260	0.674	0.924	0.348	1.176	Transport from cytoplasm to Golgi system of UDP-GalNAc and UDP-GlcA
Slc35d2	Mm01304830_m1		Not detected		Not detected		Not detected		Transport from cytoplasm to Golgi system of UDP-GlcNAc
Slc35d3	Mm01717003_m1		Not detected		Not detected		Not detected		Paralog of Slc35d1
Uap1	Mm00505476_m1	*Tbp*	0.185	1.324	0.557	1.149	0.081	1.635	UDP-activation of GlcNAc-1-P and GalNAc-1-P
Ugdh	Mm00447643_m1	*Tbp*	0.170	1.316	0.778	0.957	0.401	1.295	Conversion of UDP-glucose into UDP-glucuronic acid (UDP-GlcA)
Ugp2	Mm00454826_m1	*Tbp*	0.163	0.659	0.780	1.059	0.233	1.350	UDP-activation of Glucose-1-P
Uxs1	Mm00550735_m1	*Tbp*	0.771	1.052	0.843	1.036	0.462	1.226	Conversion of UDP-GlcA into UDP-xylose

Core proteins

Gene symbol	Taqman assay ID	Reference gene	E14vsP90 P-value	E14vsP90 Relative fold expression	E16vsP90 P-value	E16vsP90 Relative fold expression	P1vsP90 P-value	P1vsP90 Relative fold expression	Function
Agrn	Mm01545840_m1	*Tbp*	0.042	1.517	0.710	1.100	0.208	1.295	Proteoglycan carring HS present in ECM
Cd44	Mm01277164_m1	*Gapdh*	0.018	0.536	0.171	0.637	0.223	1.385	Proteoglycan carring HS and hyaluronic acid on cell surface
Col18a1	Mm00487131_m1	*Tbp*	0.498	1.152	0.958	1.011	0.532	1.199	Proteoglycan carring HS present in basement membrane
Gpc1	Mm00497305_m1	*Tbp*	0.027	1.500	0.172	1.164	0.013	1.721	Proteoglycan carring HS present on cell surface via GPI-anchor
Gpc2	Mm00549650_m1	*Tbp*	0.005	11.686	0.638	1.294	0.369	1.249	Proteoglycan carring HS present on cell surface via GPI-anchor
Gpc3	Mm00516722_m1	*Gapdh*	0.000	6.765	0.019	3.734	0.013	8.044	Proteoglycan carring HS present on cell surface via GPI-anchor
Gpc4	Mm00515035_m1	*Tbp*	0.033	1.339	0.799	1.041	0.185	1.707	Proteoglycan carring HS present on cell surface via GPI-anchor
Gpc5	Mm00615599_m1		Not detected		Not detected		Not detected		Proteoglycan carring HS present on cell surface via GPI-anchor
Gpc6	Mm00516235_m1	*Tbp*	0.027	2.880	0.275	1.376	0.130	1.926	Proteoglycan carring HS present on cell surface via GPI-anchor
Hspg2	Mm01181179_g1	*Tbp*	0.030	1.504	0.064	1.959	0.007	3.325	Proteoglycan carring HS present in ECM
Sdc1	Mm00448918_m1	*Tbp*	0.012	0.293	0.107	0.313	0.069	0.403	Proteoglycan carring HS present on cell surface
Sdc2	Mm00484718_m1	*Tbp*	0.078	1.281	0.159	0.739	0.554	1.204	Proteoglycan carring HS present on cell surface
Sdc3	Mm01179831_m1	*Tbp*	0.210	1.333	0.051	0.523	0.590	0.863	Proteoglycan carring HS present on cell surface
Sdc4	Mm00488527_m1	*Gapdh*	0.002	0.201	0.039	0.240	0.065	0.471	Proteoglycan carring HS present on cell surface

Preparation of linkage region

Gene symbol	Taqman assay ID	Reference gene	E14vsP90 P-value	E14vsP90 Relative fold expression	E16vsP90 P-value	E16vsP90 Relative fold expression	P1vsP90 P-value	P1vsP90 Relative fold expression	Function
B3galt6	Mm00504458_s1	*Tbp*	0.917	1.018	0.822	1.054	0.171	1.360	Transferring UDP-galactose to galactose-xylose on proteoglycan
B3gat1	Mm00661499_m1		Not detected		Not detected		Not detected		Transferring UDP-GlcA to galactose-galactose-xylose on proteoglycan
B3gat2	Mm00549042_m1		Not detected		Not detected		Not detected		Transferring UDP-GlcA to galactose-galactose-xylose on proteoglycan
B3gat3	Mm00470389_m1	*Tbp*	0.140	1.254	0.438	1.099	0.199	1.223	Transferring UDP-GlcA to galactose-galactose-xylose on proteoglycan
B4galt2	Mm00479556_m1	*Tbp*	0.023	4.562	0.059	2.679	0.038	3.384	Transfering UDP-galactose to GlcNAc, Glc, and Xyl.
B4galt7	Mm00461357_m1	*Tbp*	0.013	1.824	0.394	1.144	0.078	1.469	Transferring UDP-galactose to xylose on proteoglycan
Xylt1	Mm00558690_m1	*Tbp*	0.113	1.258	0.020	0.614	0.114	1.391	Transferring UDP-xylose to proteoglycan
Xylt2	Mm00461181_m1	*Tbp*	0.017	1.209	0.882	0.983	0.054	1.587	Transferring UDP-xylose to proteoglycan

GAG chain polymerisation

Gene symbol	Taqman assay ID	Reference gene	E14vsP90		E16vsP90		P1vsP90		Function
			P-value	Relative fold expression	P-value	Relative fold expression	P-value	Relative fold expression	
Chpf	Mm01262239_g1	*Tbp*	0.000	2.991	0.059	1.865	0.050	2.401	Transferring UDP-GlcA to GalNAc at end of sugar chain (CS)
Chsy1	Mm01319178_m1	*Tbp*	0.013	3.900	0.024	2.282	0.072	2.229	Transferring UDP-GlcA to GalNAc at end of sugar chain (CS)
Chsy3	Mm01545329_m1	*Tbp*	0.026	4.006	0.075	2.184	0.101	2.013	Transferring UDP-GlcA to GalNAc at end of sugar chain (CS)
Csgalnact1	Mm00555164_m1	*Tbp*	0.099	0.496	0.098	0.475	0.634	1.128	Transferring UDP-GalNAc to GlcA at end of sugar chain (CS)
Csgalnact2	Mm00513340_m1	*Tbp*	0.063	0.599	0.490	0.886	0.033	1.382	Transferring UDP-GalNAc to GlcA at end of sugar chain (CS)
Ext1	Mm00468769_m1	*Tbp*	0.491	1.076	0.897	1.013	0.236	1.180	Transferring UDP-GlcNAc to GlcA at end of sugar chain (HS)
Ext2	Mm00468775_m1		Not detected		Not detected		Not detected		Transferring UDP-GlcNAc to GlcA at end of sugar chain (HS)
Extl1	Mm00621977_s1	*Tbp*	Not detected		Not detected		Not detected		Transferring UDP-GlcA to GlcNAc at end of sugar chain (HS)
Extl2	Mm00469621_m1	*Tbp*	0.007	2.043	0.660	1.220	0.106	1.765	Transferring UDP-GlcA to GlcNAc at end of sugar chain (HS)
Extl3	Mm00516994_m1	*Tbp*	0.042	1.154	0.746	0.982	0.047	1.488	Transferring UDP-GlcA to GlcNAc at end of sugar chain (HS)
Has1	Mm00468496_m1	*Tbp*	Not detected		0.071	1.920	0.076	1.892	Polymerisation of hyaluronic acid
Has2	Mm00515089_m1	*Tbp*	0.188	1.933	0.235	1.705	0.410	1.577	Polymerisation of hyaluronic acid
Has3	Mm00515091_m1		Not detected		Not detected		Not detected		Polymerisation of hyaluronic acid

GAG chain modification

Gene symbol	Taqman assay ID	Reference gene	E14vsP90 P-value	E14vsP90 Relative fold expression	E16vsP90 P-value	E16vsP90 Relative fold expression	P1vsP90 P-value	P1vsP90 Relative fold expression	Function
Chst11	Mm00517563_m1	*Tbp*	0.002	3.000	0.087	1.195	0.087	1.957	Sulfation at the 4-O postion of GalNAc untis (CS)
Chst12	Mm00546416_s1	*Tbp*	0.168	0.806	0.698	0.936	0.270	1.433	Sulfation at the 4-O postion of GalNAc untis (CS)
Chst13	Mm01186255_s1	*Tbp*	0.371	0.490	Not detected		Not detected		Sulfation at the 4-O postion of GalNAc untis (CS)
Chst14	Mm00511291_s1	*Tbp*	0.026	2.459	0.156	1.513	0.203	1.707	Sulfation at the 4-O postion of GalNAc untis (CS)
Chst15	Mm00513227_m1	*Tbp*	0.007	1.850	0.016	1.724	0.005	1.512	Sulfation at the 6-O postion of GalNAc untis (CS)
Chst2	Mm00490018_g1	*Tbp*	0.014	3.664	0.010	3.145	0.006	2.773	Sulfation at the 6-O postion of GalNAc untis (CS)
Chst3	Mm00489736_m1	*Tbp*	0.041	3.241	0.152	1.941	0.028	3.550	Sulfation at the 6-O postion of GalNAc untis (CS)
Chst4	Mm00488783_s1		Not detected		Not detected		Not detected		Sulfation at the 6-O postion of GalNAc untis (CS)
Chst5	Mm00517342_m1		Not detected		Not detected		Not detected		Sulfation at the 6-O postion of GalNAc untis (CS)
Chst7	Mm00491466_m1	*Tbp*	0.777	1.107	0.381	1.420	Not detected		Sulfation at the 6-O postion of GalNAc untis (CS)
Chst8	Mm00558321_m1	*Tbp*	0.089	2.587	0.221	0.591	0.139	2.280	Sulfation at the 4-O postion of GalNAc untis (CS)
Chst9	Mm01722279_m1		Not detected		Not detected		Not detected		Sulfation at the 4-O postion of GalNAc untis (CS)
Dse	Mm00552923_m1	*Tbp*	0.275	1.092	0.197	0.838	0.723	1.076	Epimerisation of GlcA unit into iduronic acid unit (IdoA) (CS)
Glce	Mm00473667_m1	*Tbp*	0.146	1.381	0.636	0.918	0.094	1.632	Epimerisation of GlcA unit into iduronic acid unit (IdoA) (HS)
Hs2st1	Mm00478684_m1	*Tbp*	0.120	1.335	0.780	1.071	0.179	1.633	Sulfation at the 2-O position of GlcA and IdoA units (HS)
Hs3st1	Mm01964038_s1	*Tbp*	0.051	1.796	0.039	1.937	0.027	2.809	Sulfation at the 3-O position of GlcNAc and GlcNS units sulfated at the 6-O position (HS)
Hs3st2	Mm00616933_m1		Not detected		Not detected		Not detected		Sulfation at the 3-O position of GlcNAc and GlcNS units sulfated at the 6-O position (HS)
Hs3st3a1	Mm00780907_s1		Not detected		Not detected		Not detected		Sulfation at the 3-O position of GlcNAc and GlcNS units sulfated at the 6-O position (HS)
Hs3st3b1	Mm00479621_m1	*Tbp*	0.004	3.204	0.028	2.511	0.002	2.629	Sulfation at the 3-O position of GlcNAc and GlcNS units sulfated at the 6-O position (HS)
Hs3st6	Mm01299930_m1	*Tbp*	0.006	0.208	0.089	0.664	0.041	1.765	Sulfation at the 3-O position of GlcNAc and GlcNS units sulfated at the 6-O position (HS)
Hs6st1	Mm01229698_s1	*Tbp*	0.437	1.180	0.670	1.068	0.110	1.570	Sulfation at the 6-O position of GlcNAc and N-sulfated-glucuronic acid units (GlcNS) (HS)
Hs6st2	Mm00479296_m1		Not detected		Not detected		Not detected		Sulfation at the 6-O position of GlcNAc and GlcNS units (HS)
Hs6st3	Mm00479297_m1		Not detected		Not detected		Not detected		Sulfation at the 6-O position of GlcNAc and GlcNS units (HS)
Ndst1	Mm00447005_m1	*Tbp*	0.118	1.487	0.140	1.449	0.054	2.202	N-de-acetylation and N-sulfation of GlcNAc unit (HS)
Ndst2	Mm00447818_m1	*Tbp*	0.008	1.347	0.001	2.017	0.002	2.021	N-de-acetylation and N-sulfation of GlcNAc unit (HS)
Ndst3	Mm00453178_m1	*Tbp*	0.004	4.708	0.041	7.910	0.004	12.034	N-de-acetylation and N-sulfation of GlcNAc unit (HS)
Ndst4	Mm00480767_m1	*Tbp*	Not detected		Not detected		Not detected		N-de-acetylation and N-sulfation of GlcNAc unit (HS)
Sulf1	Mm00552283_m1	*Tbp*	0.004	4.644	0.079	2.674	0.089	2.077	Desulfation of the 6-O positon of 6-O sulfated GlcNAc and GlcNS units (HS)
Sulf2	Mm00511193_m1		Not detected		Not detected		Not detected		Desulfation of the 6-O positon of 6-O sulfated GlcNAc and GlcNS units (HS)
Sumf1	Mm00461213_m1	*Tbp*	0.021	0.784	0.023	0.573	0.960	0.987	Cofactor for Sulf1 and Sulf 2 (HS)
Sumf2	Mm01197721_m1	*Tbp*	0.008	2.657	0.104	2.023	0.038	1.857	Cofactor for Sulf1 and Sulf 2 (HS)
Ust	Mm00616790_m1	*Tbp*	0.359	0.940	0.864	1.021	0.106	1.109	Sulfation at the 2-O position of GlcA and IdoA units (CS)

GAG chain degradation

Gene symbol	Taqman assay ID	Reference gene	E14vsP90 P-value	E14vsP90 Relative fold expression	E16vsP90 P-value	E16vsP90 Relative fold expression	P1vsP90 P-value	P1vsP90 Relative fold expression	Function
Arsb	Mm00802167_m1	*Tbp*	0.049	1.403	0.745	0.944	0.979	0.987	Desulfation of the 4-O position of 4-O sulfated GalNAc units (CS)
Arsj	Mm00557970_m1	*Tbp*	0.013	7.805	0.010	12.075	0.014	7.146	Desulfation of the 4-O position of 4-O sulfated GalNAc units (CS)
Arsk	Mm00513099_m1	*Tbp*	0.306	0.801	0.059	0.466	0.143	0.678	Desulfation of proteoglycans, paralog of IDS
Galns	Mm00489575_m1	*Tbp*	0.000	2.648	0.066	1.674	0.091	1.584	Desulfation of the 6-O positon of 6-O sulfated GalNAc units (CS)
Gns	Mm00659592_m1	*Tbp*	0.103	0.540	0.153	0.532	0.668	0.842	Desulfation of the 6-O positon of 6-O sulfated GlcNAc and GlcNS units (HS)
Gusb	Mm00446954_g1	*Tbp*	0.057	1.145	0.592	0.909	0.549	1.182	Hydrolysation of GlcA from sugar chain (HS and Hyaluronic acid and CS)
Hexa	Mm00599877_m1	*Tbp*	0.104	0.552	0.140	0.568	0.261	0.735	Hydrolysation of GalNAc units (CS) and GlcNAc untis (hyaluronic acid) from sugar chain
Hexb	Mm00599880_m1	*Tbp*	0.010	0.540	0.039	0.705	0.015	0.616	Hydrolysation of GalNAc units (CS) and GlcNAc untis (hyaluronic acid) from sugar chain
Hgsnat	Mm00519485_m1	*Tbp*	0.135	0.792	0.456	0.880	0.004	1.851	Acetylation of desulfated GlcNS units (HS)
Hpse	Mm00461768_m1	*Tbp*	0.044	0.304	0.342	1.450	0.169	0.578	Degradation of HS chains extracellularly
Hyal1	Mm00476206_m1	*Tbp*	0.001	0.198	0.008	0.288	0.006	0.607	Degradation of hyaluronic acid chain
Hyal2	Mm00477731_m1	*Tbp*	Not detected		0.692	1.129	0.436	1.332	Degradation of hyaluronic acid chain
Hyal3	Mm00662097_m1		Not detected		Not detected		Not detected		Degradation of hyaluronic acid chain
Ids	Mm00494868_m1	*Tbp*	0.035	0.722	0.031	0.612	0.517	0.871	Desulfation of 2-O sulfated IdoA and GlcA units (HS and CS)
Idua	Mm00515143_m1	*Tbp*	0.509	0.887	0.092	0.646	0.773	1.093	Hydrolysation of IdoA unit from sugar chain (HS and CS)
Mgea5	Mm00452409_m1	*Tbp*	0.113	1.074	0.054	0.824	0.122	1.077	Degradation of hyaluronic acid chain
Naga	Mm00476274_m1	*Tbp*	0.069	0.703	0.175	0.744	0.408	1.205	Hydrolysis N-GalNAc moieties from glycoconjugates.
Naglu	Mm00479175_m1	*Tbp*	0.130	0.746	0.105	0.583	0.758	0.898	Hydrolysation of GlcNAc from sugar chain (HS)
Sgsh	Mm00450747_m1	*Tbp*	0.055	0.647	0.002	0.435	0.644	0.897	Desulfation of GlcNS units (HS)

Growth factors

Gene symbol	Taqman assay ID	Reference gene	E14vsP90 P-value	E14vsP90 Relative fold expression	E16vsP90 P-value	E16vsP90 Relative fold expression	P1vsP90 P-value	P1vsP90 Relative fold expression	Function
Areg	Mm00437583_m1		Not detected		Not detected		0.656	0.834	Amphiregulin **
Bmp3	Mm00557790_m1	*Tbp*	0.007	4.270	0.004	7.240	0.002	11.628	Bone morphogenetic growth factor 3 **
Bmp5	Mm00432091_m1	*Tbp*	0.022	12.051	0.303	1.788	0.587	1.300	Bone morphogenetic growth factor 5 **
Ctgf	Mm01192931_g1	*Tbp*	0.668	1.079	0.011	0.232	0.076	0.580	Connective tissue growth factor, embryonic growth factor in skin development **
Fgf10	Mm00433275_m1	*Tbp*	0.063	1.649	0.050	1.801	0.093	2.262	Fibroblast growth factor 10 **
Fgf13	Mm00438910_m1	*Tbp*	0.002	3.205	0.059	1.750	0.181	1.544	Fibroblast growth factor 13 **
Fgf2	Mm01285715_m1	*Tbp*	0.277	0.651	0.166	0.539	0.382	1.446	Fibroblast growth factor 2 **
Fgf20	Mm00748347_m1		Not detected		Not detected		Not detected		Fibroblast growth factor 20 **
Fgf22	Mm00445749_m1	*Tbp*	Not detected		0.386	0.632	0.060	0.614	Fibroblast growth factor 22 **
Fgf7	Mm00433291_m1	*Tbp*	0.045	0.606	0.002	0.394	0.087	0.630	Fibroblast growth factor 7 **
Fgf8	Mm00438921_m1		Not detected		Not detected		Not detected		Fibroblast growth factor 8 **
Figf	Mm01131929_m1	*Tbp*	0.081	1.397	0.030	1.787	0.545	0.834	C-fos induced growth factor **
Gdf10	Mm03024279_s1	*Tbp*	0.015	3.181	0.454	1.143	0.166	1.519	Growth differentiation factor 10 **
Hbegf	Mm00439305_g1	*Tbp*	Not detected		0.015	0.347	0.016	0.423	Heparin-binding epidermal growth factor **
Hdgf	Mm00725733_s1	*Gapdh*	0.257	1.221	0.737	0.911	0.975	1.008	Hepatoma-derived growth factor **
Igf1	Mm00439560_m1	*Tbp*	0.320	1.207	0.217	0.705	0.364	0.790	Insulin-like growth factor 1 **
Igf2	Mm00439565_g1	*Gapdh*	0.000	592.335	0.002	338.094	0.001	416.096	Insulin-like growth factor 2 **
Nog	Mm01297833_s1	*Tbp*	0.054	2.945	0.019	2.935	0.021	3.067	Noggin **
Pdgfa	Mm01205760_m1	*Tbp*	0.021	3.005	Not detected		0.016	3.669	Platelet-derived growth factor a **
Pdgfb	Mm01298578_m1	*Tbp*	0.321	1.098	0.033	1.468	0.010	2.096	Platelet-derived growth factor b **
Pdgfc	Mm00480205_m1	*Tbp*	Not detected		0.016	2.362	Not detected		Platelet-derived growth factor c **
Pdgfd	Mm00546829_m1	*Tbp*	Not detected		0.288	0.709	0.139	1.644	Platelet-derived growth factor d **
Shh	Mm00436527_m1		Not detected		Not detected		Not detected		Sonic hedgehog **
Tgfb1	Mm01178820_m1	*Tbp*	0.027	0.540	0.488	0.817	0.157	1.372	Transforming growth factor beta 1 **
Tgfb2	Mm01321739_m1	*Tbp*	0.039	2.697	0.757	1.081	0.127	1.809	Transforming growth factor beta 2 **
Tgfb3	Mm01307950_m1	*Tbp*	0.033	2.420	0.094	1.854	0.034	2.517	Transforming growth factor beta 3 **
Vegfa	Mm01281447_m1	*Tbp*	0.394	1.108	0.112	1.613	0.461	1.358	Vascular endothelial growth factor a **
Vegfb	Mm00442102_m1		Not detected		Not detected		Not detected		Vascular endothelial growth factor b **
Vegfc	Mm00437313_m1	*Tbp*	0.024	1.839	0.303	1.250	0.015	1.996	Vascular endothelial growth factor c **
Wnt10b	Mm00442104_m1	*Tbp*	0.180	5.829	0.122	9.688	0.105	11.748	Wingless-related integration site 10b **
Wnt16	Mm00446420_m1	*Tbp*	0.066	2.094	0.016	4.809	0.014	4.362	Wingless-related integration site 16 **
Wnt2	Mm00470018_m1	*Tbp*	0.054	3.144	0.090	3.555	0.074	3.760	Wingless-related integration site 2 **
Wnt2b	Mm00437330_m1		Not detected		Not detected		Not detected		Wingless-related integration site 2b **
Wnt3a	Mm00437337_m1	*Tbp*	0.394	1.610	0.441	1.520	0.840	1.106	Wingless-related integration site 3a **
Wnt6	Mm00437353_m1	*Tbp*	0.015	11.709	0.018	10.400	0.016	10.758	Wingless-related integration site 6 **
Wnt7a	Mm00437355_m1		Not detected		Not detected		Not detected		Wingless-related integration site 7a **
Wnt7b	Mm00437357_m1	*Tbp*	0.003	4.180	0.056	6.076	0.003	4.181	Wingless-related integration site 7b **

Putatively involved

Gene symbol	Taqman assay ID	Reference gene	E14vsP90 P-value	E14vsP90 Relative fold expression	E16vsP90 P-value	E16vsP90 Relative fold expression	P1vsP90 P-value	P1vsP90 Relative fold expression	Function
18S	Hs99999901_s1		Reference gene		Reference gene		Reference gene		Reference gene
Arsa	Mm00802173_g1	*Tbp*	0.253	0.820	0.840	0.934	0.730	1.083	Desulfation of cerebroside
Arsg	Mm00546931_m1	*Tbp*	0.004	0.705	0.003	0.408	0.275	1.329	Paralog of Arsa
B3galnt1	Mm00480272_s1	*Tbp*	0.003	2.976	0.020	1.713	0.059	1.852	Transferring UDP-GalNAc or UDP-GlcNAc to N-GlcNAc, Galactose or N-GalNAc
B4galnt3	Mm01189804_m1	*Tbp*	0.020	0.319	0.030	0.417	0.403	0.874	Transferring UDP-GalNAc or UDP-GlcNAc to N-GlcNAc, Galactose or N-GalNAc
B4galnt4	Mm00625968_g1	*Tbp*	0.055	8.732	0.532	1.484	0.216	2.604	Transferring UDP-GalNAc or UDP-GlcNAc to N-GlcNAc, Galactose or N-GalNAc
C1galt1	Mm00473986_m1	*Tbp*	0.017	0.503	0.016	0.458	0.149	0.675	Transferring UDP-Gal to N-GalNAc.
C1galt1c1	Mm00480676_m1	*Tbp*	0.321	0.862	0.712	1.042	0.878	0.972	C1GALT1-specific chaperone 1
Chst1	Mm00517855_m1	*Tbp*	0.006	1.723	0.019	2.637	0.012	2.241	Involved in sulfation of keratan sulfate
Gapdh	Mm03302249_g1		Reference gene		Reference gene		Reference gene		Reference gene
Gla	Mm00516323_m1	*Tbp*	0.422	0.913	0.018	0.494	0.900	0.946	Hydrolysis of galactose units
Glb1	Mm00515342_m1	*Tbp*	0.030	1.606	0.152	1.578	0.338	1.403	Hydrolysis of galactose units
Glb1l2	Mm00464245_m1	*Tbp*	0.011	0.039	0.056	0.319	0.164	0.539	Hydrolysis of galactose units
Gnpda1	Mm01254913_g1	*Tbp*	0.046	0.702	0.045	0.548	0.374	0.821	Conversion of GlcNAc-6-P into fructose-6-P
Gnpda2	Mm00503573_m1	*Tbp*	0.112	0.772	0.453	0.857	0.135	1.355	Conversion of GlcNAc-6-P into fructose-6-P
Khk	Mm00434647_m1		Not detected		Not detected		Not detected		Phosphorylating fructose to fructose-1-P
Kl	Mm00502002_m1		Not detected		Not detected		Not detected		Putative glucuronidase
Klb	Mm00473122_m1	*Tbp*	Not detected		0.311	1.268	0.141	2.593	Putative glucuronidase
Renbp	Mm00491720_m1	*Tbp*	0.175	0.690	0.847	0.963	0.575	1.095	Concersion of N-GlcNAc to N-acetylmannosamine
Slc17a5	Mm00555344_m1	*Tbp*	0.073	0.758	0.050	0.767	0.078	1.350	Transport of GlcA from lysosome to cytoplasm
Slc26a3	Mm00445313_m1		Not detected		Not detected		Not detected		Transferring chloride ions across the cell membrane in exchange for bicarbonate ions
Slc26a4	Mm00442308_m1		Not detected		Not detected		Not detected		Transferring chloride ions across the cell membrane
Slc26a5	Mm00446145_m1		Not detected		Not detected		Not detected		Protein functions as a molecular motor in motile outer hair cells of the cochlea
Slc35a1	Mm00442341_m1	*Tbp*	0.019	1.514	0.425	1.088	0.536	0.920	Putative CMP-Sialic Acid Transporter
Tbp	Mm01277045_m1	*Tbp*	0.293	1.000	0.423	1.000	0.423	1.000	Reference gene
Ugt8a	Mm00495930_m1	*Tbp*	0.996	1.001	0.049	0.293	0.227	0.670	Transfere galactose to ceramide

* Glc, glucosamine; GlcNAc, N-acetylated glucosamine; Gal, galactosamine; GalNAc, N-acetylated galactosamine; GlcA, glucuronic acid; IdoA, iduronic acid. **Uijtdewilligen *et al.* , 2016.

Genes, for which a signal was not or only partly detected at a given timepoint or multiple timepoints and therefore a fold change and/or p-value could not be calculated based on the available data, are given as 'not detected'. Gene symbol for which all timepoints were classified as 'not detected' do not show a reference gene due to lack of data for a calculation.

Supplementary data table 5: Differentially expressed gened during skin development in mice in comparison to mature mouse skin using EXON arrays

Production of precursors

			Present on		E14vsP90		E16vsP90		P1vsP90		
Gene symbol	Taqman assay ID	Affymetrix ID	Cart V1	Cart V2	Stepup P-value	Fold change	Stepup P-value	Fold change	Stepup P-value	Fold change	Function
Adpgk	Mm00511302_m1	6989534	Yes	Yes	0.047	1.641	0.083	1.614	0.233	1.384	Phosphorylation of glucose into glucose-6-P
Gale	Mm00617772_g1	6917794	Yes	Yes	0.674	0.858	0.612	0.834	0.244	0.555	Conversion of UDP-GlcNAc to UDP-GalNAc
Galk1	Mm00444182_m1	6792485	Yes	Yes	0.030	4.242	0.126	2.483	0.231	2.194	Phosphorylation of Galactose into Galactose-1-P
Galk2	Mm00613268_m1	6880899	Yes	Yes	0.447	1.188	0.434	0.832	0.709	0.881	Phosphorylation of N-Acetylgalactosamine (GalNAc) into GalNAc-1P)
Galt	Mm00489459_g1	6912944	Yes	Yes	0.664	0.814	0.231	1.864	0.665	1.340	UDP-activation of Glucose-1-P and Galactose-1-P
Gck	Mm00439129_m1	6785753	Yes	Yes	0.045	0.640	0.489	1.125	0.305	0.787	Phosphorylation of glucose into glucose-6-P
Gfpt1	Mm00600127_m1	6947679	Yes	Yes	0.020	2.110	0.077	1.682	0.801	1.081	Conversion of fructose-6-P into glucosamine-6-P (Glc-6-P)
Gfpt2	Mm00496565_m1	6780767	Yes	Yes	0.176	0.610	0.087	0.426	0.229	0.547	Conversion of fructose-6-P into Glc-6-P
Gnpnat1	Mm00834602_mH	*Not available**	Yes	Yes	*Not available**		*Not available**		*Not available**		Conversion of Glc-6-P into N-Acetylglucosamine-6-P (GlcNAc-6-P)
Gpi1	Mm02026122_g1	6966425	Yes	Yes	0.277	1.220	0.429	1.160	0.485	1.194	Isomerisation of glucose-6-P into fructose-6-P
Hk1	Mm00439344_m1	6774391	Yes	Yes	0.469	0.824	0.549	0.849	0.185	0.588	Phosphorylation of glucose into glucose-6-P
Hk2	Mm00443385_m1	6954982	Yes	Yes	0.003	0.451	0.040	1.375	0.039	0.655	Phosphorylation of glucose into glucose-6-P
Miox	Mm00480214_m1	6832572	Yes	Yes	0.795	1.042	0.234	1.212	0.825	0.950	Conversion of Myo-inositol into UDP-GlcA
Nagk	Mm00479878_m1	6947460	Yes	Yes	0.900	1.055	0.468	0.763	0.652	0.787	Phosphorylation of GlcNAc to GlcNAC-6-P
Papss1	Mm00442283_m1	6901413	Yes	Yes	0.696	1.238	0.786	0.861	0.997	1.004	Synthesis of PAPS complex
Papss2	Mm00442295_m1	6869216	Yes	Yes	0.040	2.474	0.203	1.605	0.643	1.241	Synthesis of PAPS complex
Pgm1	Mm00804141_m1	6931181	Yes	Yes	0.088	0.689	0.050	0.535	0.109	0.605	Conversion of glucose-6-P into glucose-1-P
Pgm2	Mm00728285_s1	6915791	Yes	Yes	0.401	0.816	0.538	1.165	0.762	1.120	Conversion of glucose-6-P into glucose-1-P
Pgm3	Mm00459270_m1	6997513	Yes	Yes	0.083	2.193	0.676	1.178	0.654	1.286	Conversion of GlcNAc-6-P into GlcNAc-1-P
Pgm5	Mm00723432_m1	6872290	Yes	Yes	0.058	2.338	0.104	2.209	0.291	1.696	Conversion of glucose-6-P into glucose-1-P
Slc13a1	Mm00490339_m1	6952151	Yes	Yes	0.474	0.897	0.363	1.156	0.939	1.021	Transport of sulphate from the extracellular space to cytoplasm
Slc13a4	Mm00556495_m1	6952679	Yes	Yes	0.482	1.144	0.581	1.113	0.652	1.134	Transport of sulphate from the extracellular space to cytoplasm
Slc13a5	Mm01334459_m1	6789531	Yes	Yes	0.003	2.588	0.059	1.322	0.576	1.075	Transport of sulphate from the extracellular space to cytoplasm
Slc26a11	Mm00626632_m1	6785369	Yes	Yes	0.068	0.755	0.184	0.820	0.970	1.009	Transport of sulphate from the extracellular space to cytoplasm
Slc26a2	Mm00432896_m1	6865971	Yes	Yes	0.164	1.260	0.077	1.509	0.242	0.770	Transport of sulphate from the extracellular space to cytoplasm
Slc26a6	Mm00506742_m1	6992374	Yes	Yes	0.475	0.921	0.158	0.818	0.608	0.918	Transport of sulphate from the extracellular space to cytoplasm
Slc26a7	Mm00524162_m1	6919879	Yes	Yes	0.843	1.082	0.444	1.319	0.220	1.872	Transport of sulphate from the extracellular space to cytoplasm
Slc26a8	Mm00524836_m1	6854619	Yes	Yes	0.884	0.977	0.133	1.309	0.818	1.052	Transport of sulphate from the extracellular space to cytoplasm
Slc26a9	Mm00628490_m1	6753079	Yes	Yes	0.012	0.227	0.090	0.471	0.091	0.392	Transport of sulphate from the extracellular space to cytoplasm
Slc2a1	Mm01192270_m1	6916748	No	Yes	0.277	1.613	0.311	1.605	0.254	0.491	Sugar transport across plasma membrane
Slc2a10	Mm00453716_m1	6883184	No	Yes	0.059	1.836	0.173	1.523	0.083	2.266	Sugar transport across plasma membrane
Slc2a12	Mm00619244_m1	6766491	No	Yes	0.007	0.263	0.016	0.258	0.032	0.315	Sugar transport across plasma membrane
Slc2a2	Mm00446230_g1	6896428	No	Yes	0.461	1.176	0.257	1.318	0.811	1.087	Sugar transport across plasma membrane
Slc2a3	Mm00441483_m1	6957051	No	Yes	0.063	15.969	0.334	3.430	0.947	0.868	Sugar transport across plasma membrane
Slc2a4	Mm01245507_g1	6789367	No	Yes	0.006	0.298	0.044	0.525	0.857	1.054	Sugar transport across plasma membrane
Slc2a5	Mm00600311_m1	6918858	No	Yes	0.077	0.691	0.365	0.847	0.344	0.794	Sugar transport across plasma membrane
Slc2a6	Mm00554217_m1	6885514	No	Yes	0.021	0.667	0.740	0.964	0.168	0.799	Sugar transport across plasma membrane
Slc2a8	Mm00444634_m1	6885923	No	Yes	0.087	0.702	0.323	0.830	0.878	0.955	Sugar transport across plasma membrane
Slc2a9	Mm01211147_m1	*Not available**	No	Yes	*Not available**		*Not available**		*Not available**		Sugar transport across plasma membrane
Slc35a2	Mm00446504_m1	7009796	Yes	Yes	0.266	1.396	0.747	1.104	0.960	0.973	Transport from cytoplasm to Golgi system of UDP-GalNAc and UDP-Galactose
Slc35a3	Mm00523288_m1	6908510	Yes	Yes	0.105	0.767	0.596	0.925	0.572	0.890	Transport from cytoplasm to Golgi system of UDP-GlcNAc
Slc35a4	Mm00782997_s1	6860121	Yes	Yes	0.270	1.425	0.847	1.068	0.744	1.171	Transport from cytoplasm to Golgi system of UDP-GalNAc and UDP-Galactose
Slc35b1	Mm00450258_m1	6783740	Yes	Yes	0.723	0.903	0.184	0.651	0.716	0.863	Transport from cytoplasm to Golgi system of UDP-GalNAc and UDP-Galactose
Slc35b2	Mm01269910_m1	6850646	Yes	Yes	0.336	1.341	0.478	1.247	0.868	1.089	Transport of PAPS comples from cytoplasm to Golgi system
Slc35b3	Mm00506275_m1	6812534	Yes	Yes	0.021	1.270	0.877	0.990	0.227	1.117	Transport of PAPS comples from cytoplasm to Golgi system
Slc35b4	Mm00480588_m1	6952601	Yes	Yes	0.482	1.129	0.687	1.074	0.907	1.035	Transport from cytoplasm to Golgi system of UDP-GlcNAc and UDP-Xylose
Slc35d1	Mm00624959_m1	6924027	Yes	Yes	0.229	1.372	0.440	1.226	0.709	1.153	Transport from cytoplasm to Golgi system of UDP-GalNAc and UDP-GlcA
Slc35d2	Mm01304830_m1	6813876	Yes	Yes	0.067	0.867	0.101	0.866	0.051	0.763	Transport from cytoplasm to Golgi system of UDP-GlcNAc
Slc35d3	Mm01717003_m1	6772476	Yes	Yes	0.133	1.509	0.078	1.932	0.344	1.391	Paralog of Slc35d1
Uap1	Mm00505476_m1	6764011	Yes	Yes	0.168	1.335	0.758	1.065	0.785	1.088	UDP-activation of GlcNAc-1-P and GalNAc-1-P

Ugdh	Mm00447643_m1	6938704	Yes	Yes	0.100	1.282	0.451	0.903	0.558	1.117	Conversion of UDP-glucose into UDP-glucuronic acid (UDP-GlcA)
Ugp2	Mm00454826_m1	6786561	Yes	Yes	0.203	1.203	0.956	0.991	0.215	1.287	UDP-activation of Glucose-1-P
Uxs1	Mm00550735_m1	6758325	Yes	Yes	0.595	1.160	0.692	0.894	0.865	0.925	Conversion of UDP-GlcA into UDP-xylose

Core proteins

			Present on		E14vsP90		E16vsP90		P1vsP90		
**Gene symbol**	**Taqman assay ID**	**Affymetrix ID**	**Cart V1**	**Cart V2**	**Stepup P-value**	**Fold change**	**Stepup P-value**	**Fold change**	**Stepup P-value**	**Fold change**	**Function**
Acan	Mm00545807_m1	6961912	No	Yes	0.007	17.401	0.744	0.857	0.890	1.114	Proteoglycan carring CS or DS
Agrn	Mm01545840_m1	6927362	Yes	Yes	0.682	1.082	0.272	1.252	0.892	1.044	Proteoglycan carring HS present in ECM
Aspn	Mm00445945_m1	6806958	No	Yes	0.074	2.199	0.406	1.373	0.088	3.129	Proteoglycan carring CS or DS
Bcan	Mm00476090_m1	6906694	No	Yes	0.663	1.097	0.255	1.297	0.919	0.964	Proteoglycan carring CS or DS
Bgn	Mm00455918_m1	7011936	No	Yes	0.017	0.721	0.054	0.776	0.061	1.352	Proteoglycan carring CS or DS
Cd44	Mm01277164_m1	6889258	Yes	Yes	0.009	0.370	0.144	0.728	0.873	0.955	Proteoglycan carring HS and hyaluronic acid on cell surface
Col18a1	Mm00487131_m1	6775201	Yes	Yes	0.440	0.885	0.324	0.847	0.359	0.818	Proteoglycan carring HS present in basement membrane
Col9a2	Mm00483872_m1	6916849	No	Yes	0.012	5.104	0.292	1.462	0.997	1.003	Proteoglycan carring CS or DS
Cspg4	Mm00507256_m1	6989349	No	Yes	0.693	0.918	0.636	0.905	0.693	1.130	Proteoglycan carring CS or DS
Cspg5	Mm00516549_m1	6992428	No	Yes	0.015	2.593	0.099	1.639	0.506	1.225	Proteoglycan carring CS or DS
Dcn	Mm03003496_s1	6770064	No	Yes	0.221	0.705	0.252	0.703	0.399	0.723	Proteoglycan carring CS or DS
Epyc	Mm00514611_m1	6770076	No	Yes	0.001	21.280	0.365	1.162	0.597	0.886	Proteoglycan carring CS or DS
Gpc1	Mm00497305_m1	6751634	Yes	Yes	0.404	1.111	0.305	1.150	0.312	1.197	Proteoglycan carring HS present on cell surface via GPI-anchor
Gpc2	Mm00549650_m1	*Not available**	Yes	Yes	*Not available**		*Not available**		*Not available**		Proteoglycan carring HS present on cell surface via GPI-anchor
Gpc3	Mm00516722_m1	7016826	Yes	Yes	0.003	4.339	0.013	3.305	0.015	4.721	Proteoglycan carring HS present on cell surface via GPI-anchor
Gpc4	Mm00515035_m1	7016823	Yes	Yes	0.667	1.117	0.723	0.913	0.694	1.157	Proteoglycan carring HS present on cell surface via GPI-anchor
Gpc5	Mm00615599_m1	6821935	Yes	Yes	0.342	1.092	0.461	1.073	0.411	0.899	Proteoglycan carring HS present on cell surface via GPI-anchor
Gpc6	Mm00516235_m1	6821985	Yes	Yes	0.019	2.767	0.109	1.747	0.193	1.640	Proteoglycan carring HS present on cell surface via GPI-anchor
Hmmr	Mm00469183_m1	6787469	No	Yes	0.059	3.525	0.191	2.263	0.987	0.984	Hyaluronan-Mediated Motility Receptor
Hspg2	Mm01181179_g1	6917933	Yes	Yes	0.309	1.183	0.075	1.554	0.042	2.230	Proteoglycan carring HS present in ECM
Ncan	Mm00484007_m1	6983162	No	Yes	0.001	8.732	0.101	1.213	0.908	1.017	Proteoglycan carring CS or DS
Sdc1	Mm00448918_m1	6793226	Yes	Yes	0.031	0.380	0.067	0.413	0.103	0.424	Proteoglycan carring HS present on cell surface
Sdc2	Mm00484718_m1	6829598	Yes	Yes	0.479	1.406	0.545	0.744	0.669	1.356	Proteoglycan carring HS present on cell surface
Sdc3	Mm01179831_m1	6917389	Yes	Yes	0.987	1.006	0.107	0.539	0.240	0.622	Proteoglycan carring HS present on cell surface
Sdc4	Mm00488527_m1	6892905	Yes	Yes	0.017	0.341	0.094	0.536	0.215	0.621	Proteoglycan carring HS present on cell surface
Smc3	Mm00484015_m1	6870470	No	Yes	0.007	1.969	0.049	1.438	0.112	1.329	Proteoglycan carring CS or DS
Srgn	Mm01169070_m1	6774400	No	Yes	0.224	0.671	0.856	1.064	0.631	0.799	Proteoglycan carring CS or DS
Vcan	Mm01283063_m1	6814956	No	Yes	0.006	3.673	0.023	2.792	0.029	3.226	Proteoglycan carring CS or DS

Preparation of linkage region

			Present on		E14vsP90		E16vsP90		P1vsP90		
**Gene symbol**	**Taqman assay ID**	**Affymetrix ID**	**Cart V1**	**Cart V2**	**Stepup P-value**	**Fold change**	**Stepup P-value**	**Fold change**	**Stepup P-value**	**Fold change**	**Function**
B3galt6	Mm00504458_s1	6927341	Yes	Yes	0.764	1.202	0.764	0.837	0.708	1.373	Transferring UDP-galactose to galactose-xylose on proteoglycan
B3gat1	Mm00661499_m1	6987632	Yes	Yes	0.008	3.467	0.256	1.302	0.828	0.929	Transferring UDP-GlcA to galactose-galactose-xylose on proteoglycan
B3gat2	Mm00549042_m1	6748174	Yes	Yes	0.009	3.241	0.286	1.269	0.690	1.129	Transferring UDP-GlcA to galactose-galactose-xylose on proteoglycan
B3gat3	Mm00470389_m1	6867978	Yes	Yes	0.672	0.954	0.295	0.886	0.349	0.870	Transferring UDP-GlcA to galactose-galactose-xylose on proteoglycan
B4galt2	Mm00479556_m1	6924869	Yes	Yes	0.039	1.726	0.053	1.865	0.176	1.477	Transfering UDP-galactose to GlcNAc, Glc, and Xyl.
B4galt7	Mm00461357_m1	6807252	Yes	Yes	0.135	1.221	0.701	1.049	0.710	0.935	Transferring UDP-galactose to xylose on proteoglycan
Xylt1	Mm00558690_m1	6963793	Yes	Yes	0.957	1.005	0.390	0.935	0.108	1.224	Transferring UDP-xylose to proteoglycan
Xylt2	Mm00461181_m1	6790972	Yes	Yes	0.801	0.950	0.957	0.988	0.820	1.070	Transferring UDP-xylose to proteoglycan

GAG chain polymerisation

Gene symbol	Taqman assay ID	Affymetrix ID	Present on Cart V1	Present on Cart V2	E14vsP90 Stepup P-value	E14vsP90 Fold change	E16vsP90 Stepup P-value	E16vsP90 Fold change	P1vsP90 Stepup P-value	P1vsP90 Fold change	Function
Chpf	Mm01262239_g1	6759816	Yes	Yes	0.071	1.679	0.182	1.459	0.309	1.403	Transferring UDP-GlcA to GalNAc at end of sugar chain (CS)
Chsy1	Mm01319178_m1	*Not available**	Yes	Yes	*Not available**		*Not available**		*Not available**		Transferring UDP-GlcA to GalNAc at end of sugar chain (CS)
Chsy3	Mm01545329_m1	6861281	Yes	Yes	0.429	1.153	0.056	1.769	0.849	1.058	Transferring UDP-GlcA to GalNAc at end of sugar chain (CS)
Csgalnact1	Mm00555164_m1	6983073	Yes	Yes	0.137	0.512	0.202	0.541	0.974	0.975	Transferring UDP-GalNAc to GlcA at end of sugar chain (CS)
Csgalnact2	Mm00513340_m1	6956864	Yes	Yes	0.475	0.819	0.734	1.103	0.407	1.382	Transferring UDP-GalNAc to GlcA at end of sugar chain (CS)
Ext1	Mm00468769_m1	6835692	Yes	Yes	0.881	1.024	0.351	0.873	0.936	0.980	Transferring UDP-GlcNAc to GlcA at end of sugar chain (HS)
Ext2	Mm00468775_m1	6888925	Yes	Yes	0.086	1.336	0.319	1.166	0.358	1.202	Transferring UDP-GlcNAc to GlcA at end of sugar chain (HS)
Extl1	Mm00621977_s1	6926017	Yes	Yes	0.025	0.407	0.149	0.625	0.114	0.505	Transferring UDP-GlcA to GlcNAc at end of sugar chain (HS)
Extl2	Mm00469621_m1	6900659	Yes	Yes	0.085	1.702	0.819	1.066	0.518	1.272	Transferring UDP-GlcA to GlcNAc at end of sugar chain (HS)
Extl3	Mm00516994_m1	6825402	Yes	Yes	0.895	1.024	0.723	0.944	0.312	1.250	Transferring UDP-GlcA to GlcNAc at end of sugar chain (HS)
Has1	Mm00468496_m1	6854042	Yes	Yes	0.465	0.793	0.226	1.548	0.278	1.628	Polymerisation of hyaluronic acid
Has2	Mm00515089_m1	6835856	Yes	Yes	0.095	2.107	0.157	1.969	0.537	1.403	Polymerisation of hyaluronic acid
Has3	Mm00515091_m1	6978932	Yes	Yes	0.861	1.149	0.128	3.994	0.202	3.726	Polymerisation of hyaluronic acid

GAG chain modification

Gene symbol	Taqman assay ID	Affymetrix ID	Present on Cart V1	Present on Cart V2	E14vsP90 Stepup P-value	E14vsP90 Fold change	E16vsP90 Stepup P-value	E16vsP90 Fold change	P1vsP90 Stepup P-value	P1vsP90 Fold change	Function
Chst11	Mm00517563_m1	6769366	Yes	Yes	0.049	1.922	0.465	1.208	0.239	1.531	Sulfation at the 4-O postion of GalNAc untis (CS)
Chst12	Mm00546416_s1	6935275	Yes	Yes	0.448	0.646	0.632	0.756	0.995	0.993	Sulfation at the 4-O postion of GalNAc untis (CS)
Chst13	Mm01186255_s1	*Not available**	Yes	Yes	*Not available**		*Not available**		*Not available**		Sulfation at the 4-O postion of GalNAc untis (CS)
Chst14	Mm00511291_s1	6880476	Yes	Yes	0.151	1.491	0.506	1.191	0.773	1.122	Sulfation at the 4-O postion of GalNAc untis (CS)
Chst15	Mm00513227_m1	6971656	Yes	Yes	0.027	1.831	0.039	1.991	0.207	1.396	Sulfation at the 6-O postion of GalNAc untis (CS)
Chst2	Mm00490018_g1	6997990	Yes	Yes	0.035	2.302	0.072	2.160	0.207	1.682	Sulfation at the 6-O postion of GalNAc untis (CS)
Chst3	Mm00489736_m1	6774295	Yes	Yes	0.885	1.034	0.108	1.545	0.162	1.530	Sulfation at the 6-O postion of GalNAc untis (CS)
Chst4	Mm00488783_s1	6985292	Yes	No	0.197	0.696	0.254	1.407	0.912	0.951	Sulfation at the 6-O postion of GalNAc untis (CS)
Chst5	Mm00517342_m1	6985414	Yes	Yes	0.069	1.794	0.130	1.677	0.642	1.196	Sulfation at the 6-O postion of GalNAc untis (CS)
Chst7	Mm00491466_m1	7010308	Yes	Yes	0.928	1.056	0.971	1.023	0.998	0.997	Sulfation at the 6-O postion of GalNAc untis (CS)
Chst8	Mm00558321_m1	6966453	Yes	Yes	0.945	1.007	0.569	1.054	0.420	1.108	Sulfation at the 4-O postion of GalNAc untis (CS)
Chst9	Mm01722279_m1	6863607	Yes	Yes	0.122	1.172	0.122	1.209	0.562	1.080	Sulfation at the 4-O postion of GalNAc untis (CS)
Dse	Mm00552923_m1	6773174	Yes	Yes	0.916	1.028	0.407	0.826	0.837	0.928	Epimerisation of GlcA unit into iduronic acid unit (IdoA) (CS)
Glce	Mm00473667_m1	6996191	Yes	Yes	0.059	1.280	0.189	1.175	0.086	1.385	Epimerisation of GlcA unit into iduronic acid unit (IdoA) (HS)
Hs2st1	Mm00478684_m1	6910088	Yes	Yes	0.080	1.553	0.453	1.178	0.195	1.504	Sulfation at the 2-O position of GlcA and IdoA units (HS)
Hs3st1	Mm01964038_s1	6937654	Yes	Yes	0.074	1.658	0.105	1.688	0.119	1.824	Sulfation at the 3-O position of GlcNAc and GlcNS units sulfated at the 6-O position (HS)
Hs3st2	Mm00616933_m1	6963989	Yes	Yes	0.951	1.017	0.215	1.380	0.934	0.967	Sulfation at the 3-O position of GlcNAc and GlcNS units sulfated at the 6-O position (HS)
Hs3st3a1	Mm00780907_s1	6781689	Yes	Yes	0.878	1.068	0.910	1.047	0.945	0.955	Sulfation at the 3-O position of GlcNAc and GlcNS units sulfated at the 6-O position (HS)
Hs3st3b1	Mm00479621_m1	6788991	Yes	Yes	0.683	1.178	0.253	1.634	0.511	1.432	Sulfation at the 3-O position of GlcNAc and GlcNS units sulfated at the 6-O position (HS)
Hs3st5	Mm01192940_m1	*Not available**	No	Yes	*Not available**		*Not available**		*Not available**		Sulfation at the 3-O position of GlcNAc and GlcNS units sulfated at the 6-O position (HS)
Hs3st6	Mm01299930_m1	6849317	Yes	Yes	0.019	0.406	0.291	0.771	0.721	1.130	Sulfation at the 3-O position of GlcNAc and GlcNS units sulfated at the 6-O position (HS)
Hs6st1	Mm01229698_s1	6748605	Yes	Yes	0.610	1.095	0.808	1.046	0.982	1.007	Sulfation at the 6-O position of GlcNAc and N-sulfated-glucuronic acid units (GlcNS) (HS)
Hs6st2	Mm00479296_m1	7016808	Yes	Yes	0.004	6.417	0.012	5.609	0.041	3.080	Sulfation at the 6-O position of GlcNAc and GlcNS units (HS)
Hs6st3	Mm00479297_m1	6822138	Yes	Yes	0.170	1.305	0.197	1.319	0.897	1.041	Sulfation at the 6-O position of GlcNAc and GlcNS units (HS)
Ndst1	Mm00447005_m1	6865926	Yes	Yes	0.090	1.363	0.056	1.649	0.067	1.763	N-de-acetylation and N-sulfation of GlcNAc unit (HS)
Ndst2	Mm00447818_m1	6823122	Yes	Yes	0.822	1.044	0.059	1.697	0.083	1.733	N-de-acetylation and N-sulfation of GlcNAc unit (HS)
Ndst3	Mm00453178_m1	6908958	Yes	Yes	0.173	2.472	0.111	3.856	0.122	4.860	N-de-acetylation and N-sulfation of GlcNAc unit (HS)
Ndst4	Mm00480767_m1	6901119	Yes	Yes	0.005	1.667	0.438	1.059	0.913	0.987	N-de-acetylation and N-sulfation of GlcNAc unit (HS)
Sulf1	Mm00552283_m1	6747641	Yes	Yes	0.010	4.522	0.051	2.546	0.232	1.627	Desulfation of the 6-O positon of 6-O sulfated GlcNAc and GlcNS units (HS)
Sulf2	Mm00511193_m1	6893057	Yes	Yes	0.229	1.232	0.580	0.909	0.101	1.609	Desulfation of the 6-O positon of 6-O sulfated GlcNAc and GlcNS units (HS)
Sumf1	Mm00461213_m1	6956297	Yes	Yes	0.487	0.744	0.281	0.607	0.785	0.834	Cofactor for Sulf1 and Sulf 2 (HS)
Sumf2	Mm01197721_m1	*Not available**	Yes	Yes	*Not available**		*Not available**		*Not available**		Cofactor for Sulf1 and Sulf 2 (HS)
Ust	Mm00616790_m1	6771995	Yes	Yes	0.875	0.926	0.927	1.044	0.884	1.110	Sulfation at the 2-O position of GlcA and IdoA units (CS)

GAG chain degradation

Gene symbol	Taqman assay ID	Affymetrix ID	Present on Cart V1	Present on Cart V2	E14vsP90 Stepup P-value	E14vsP90 Fold change	E16vsP90 Stepup P-value	E16vsP90 Fold change	P1vsP90 Stepup P-value	P1vsP90 Fold change	Function
Arsb	Mm00802167_m1	6809030	Yes	Yes	0.519	1.139	0.360	1.214	0.808	0.925	Desulfation of the 4-O position of 4-O sulfated GalNAc units (CS)
Arsj	Mm00557970_m1	6901136	Yes	Yes	0.003	2.498	0.006	3.164	0.031	1.702	Desulfation of the 4-O position of 4-O sulfated GalNAc units (CS)
Arsk	Mm00513099_m1	6814451	Yes	Yes	0.368	0.784	0.102	0.543	0.514	0.779	Desulfation of proteoglycans, paralog of IDS
Galns	Mm00489575_m1	6985943	Yes	Yes	0.087	1.671	0.322	1.311	0.786	1.115	Desulfation of the 6-O positon of 6-O sulfated GalNAc units (CS)
Gns	Mm00659592_m1	6771224	Yes	Yes	0.057	0.586	0.111	0.622	0.429	0.784	Desulfation of the 6-O positon of 6-O sulfated GlcNAc and GlcNS units (HS)
Gusb	Mm00446954_g1	6942192	Yes	Yes	0.923	0.971	0.499	0.832	0.843	0.918	Hydrolysation of GlcA from sugar chain (HS and Hyaluronic acid and CS)
Hexa	Mm00599877_m1	6989553	Yes	Yes	0.262	0.619	0.204	0.535	0.358	0.583	Hydrolysation of GalNAc units (CS) and GlcNAc untis (hyaluronic acid) from sugar chain
Hexb	Mm00599880_m1	6815345	Yes	Yes	0.204	0.611	0.130	0.480	0.287	0.570	Hydrolysation of GalNAc units (CS) and GlcNAc untis (hyaluronic acid) from sugar chain
Hgsnat	Mm00519485_m1	6981182	Yes	Yes	0.425	0.826	0.355	0.791	0.290	1.432	Acetylation of desulfated GlcNS units (HS)
Hpse	Mm00461768_m1	6940363	Yes	Yes	0.021	0.267	0.540	1.244	0.095	0.360	Degradation of HS chains extracellularly
Hyal1	Mm00476206_m1	6992224	Yes	Yes	0.003	0.154	0.011	0.222	0.041	0.402	Degradation of hyaluronic acid chain
Hyal2	Mm00477731_m1	6992221	Yes	Yes	0.410	1.167	0.653	0.918	0.610	1.149	Degradation of hyaluronic acid chain
Hyal3	Mm00662097_m1	6992228	Yes	Yes	0.499	0.892	0.166	1.329	0.599	1.137	Degradation of hyaluronic acid chain
Ids	Mm00494868_m1	7017419	Yes	Yes	0.292	0.752	0.161	0.630	0.629	0.826	Desulfation of 2-O sulfated IdoA and GlcA units (HS and CS)
Idua	Mm00515143_m1	6933337	Yes	Yes	0.089	0.689	0.259	0.790	0.721	0.904	Hydrolysation of IdoA unit from sugar chain (HS and CS)
Mgea5	Mm00452409_m1	6873365	Yes	Yes	0.893	0.935	0.973	1.017	0.968	0.969	Degradation of hyaluronic acid chain
Naga	Mm00476274_m1	6837368	Yes	Yes	0.071	0.544	0.110	0.550	0.531	0.782	Hydrolysis N-GalNAc moieties from glycoconjugates.
Naglu	Mm00479175_m1	6784237	Yes	Yes	0.182	0.569	0.202	0.551	0.537	0.704	Hydrolysation of GlcNAc from sugar chain (HS)
Sgsh	Mm00450747_m1	6792702	Yes	Yes	0.017	0.458	0.052	0.537	0.201	0.699	Desulfation of GlcNS units (HS)

Growth factors

Gene symbol	Taqman assay ID	Affymetrix ID	Present on Cart V1	Present on Cart V2	E14vsP90 Stepup P-value	E14vsP90 Fold change	E16vsP90 Stepup P-value	E16vsP90 Fold change	P1vsP90 Stepup P-value	P1vsP90 Fold change	Function
Areg	Mm00437583_m1	6932394	Yes	No	0.016	0.157	0.043	0.200	0.333	0.550	Amphiregulin **
Bmp3	Mm00557790_m1	6932718	Yes	No	0.280	1.392	0.067	2.381	0.100	2.365	Bone morphogenetic growth factor 3 **
Bmp5	Mm00432091_m1	6990569	Yes	No	0.001	7.247	0.141	1.191	0.688	1.057	Bone morphogenetic growth factor 5 **
Ctgf	Mm01192931_g1	6766623	Yes	Yes	0.148	0.729	0.025	0.366	0.068	0.485	Connective tissue growth factor, embryonic growth factor in skin development **
Fgf10	Mm00433275_m1	6810592	Yes	Yes	0.138	1.521	0.057	2.250	0.094	2.142	Fibroblast growth factor 10 **
Fgf13	Mm00438910_m1	7017134	Yes	Yes	0.012	2.998	0.075	1.824	0.296	1.391	Fibroblast growth factor 13 **
Fgf2	Mm01285715_m1	6896850	Yes	Yes	0.201	0.664	0.279	0.696	0.952	0.967	Fibroblast growth factor 2 **
Fgf20	Mm00748347_m1	6981854	Yes	No	0.659	1.228	0.125	2.483	0.777	1.220	Fibroblast growth factor 20 **
Fgf22	Mm00445749_m1	6769141	Yes	Yes	0.005	0.329	0.972	0.994	0.097	0.646	Fibroblast growth factor 22 **
Fgf4	Mm00438917_m1	*Not available**	No	Yes	*Not available**		*Not available**		*Not available**		Growth factor (Uijtdewilligen et al, 2013)**
Fgf7	Mm00433291_m1	6880900	Yes	Yes	0.531	0.740	0.078	0.286	0.212	0.418	Fibroblast growth factor 7 **
Fgf8	Mm00438921_m1	6873363	Yes	Yes	0.492	0.883	0.655	1.085	0.682	0.895	Fibroblast growth factor 8 **
Figf	Mm01131929_m1	7015007	Yes	Yes	0.051	1.734	0.062	1.909	0.548	0.832	C-fos induced growth factor **
Gdf10	Mm03024279_s1	6818153	Yes	No	0.028	2.334	0.757	1.086	0.894	1.060	Growth differentiation factor 10 **
Hbegf	Mm00439305_g1	6864680	Yes	Yes	0.037	0.551	0.063	0.547	0.087	0.530	Heparin-binding epidermal growth factor **
Hdgf	Mm00725733_s1	6899028	Yes	No	0.104	1.246	0.575	1.070	0.409	1.147	Hepatoma-derived growth factor **
Igf1	Mm00439560_m1	6769597	Yes	No	0.235	0.631	0.173	0.537	0.398	0.641	Insulin-like growth factor 1 **
Igf2	Mm00439565_g1	6972317	Yes	Yes	0.001	59.615	0.002	55.864	0.002	52.364	Insulin-like growth factor 2 **
Nog	Mm01297833_s1	6790670	Yes	No	0.275	2.605	0.517	1.773	0.677	1.712	Noggin **
Pdgfa	Mm01205760_m1	6942654	Yes	Yes	0.028	2.013	0.035	2.347	0.057	2.338	Platelet-derived growth factor a **
Pdgfb	Mm01298578_m1	6837144	Yes	Yes	0.037	0.704	0.199	1.197	0.157	1.298	Platelet-derived growth factor b **
Pdgfc	Mm00480205_m1	6898686	Yes	Yes	0.019	2.152	0.035	2.207	0.902	1.042	Platelet-derived growth factor c **
Pdgfd	Mm00546829_m1	6986677	Yes	Yes	0.925	0.952	0.191	0.504	0.790	1.205	Platelet-derived growth factor d **
Shh	Mm00436527_m1	6936889	Yes	Yes	0.448	0.595	0.308	2.117	0.152	4.571	Sonic hedgehog **
Tgfb1	Mm01178820_m1	6959236	Yes	Yes	0.106	0.552	0.241	0.653	0.975	0.981	Transforming growth factor beta 1 **
Tgfb2	Mm01321739_m1	6764953	Yes	Yes	0.057	2.441	0.448	1.335	0.264	1.802	Transforming growth factor beta 2 **
Tgfb3	Mm01307950_m1	6802449	Yes	Yes	0.026	2.386	0.238	1.408	0.100	2.077	Transforming growth factor beta 3 **
Vegfa	Mm01281447_m1	6855659	Yes	Yes	0.779	0.875	0.721	1.174	0.940	1.061	Vascular endothelial growth factor a **
Vegfb	Mm00442102_m1	6871273	Yes	Yes	0.008	1.461	0.031	1.318	0.060	1.277	Vascular endothelial growth factor b **
Vegfc	Mm00437313_m1	6976200	Yes	Yes	0.088	2.120	0.477	1.316	0.316	1.700	Vascular endothelial growth factor c **
Wnt10b	Mm00442104_m1	6838399	Yes	No	0.629	1.280	0.120	2.812	0.240	2.325	Wingless-related integration site 10b **
Wnt16	Mm00446420_m1	6944581	Yes	No	0.358	1.180	0.026	2.402	0.093	1.710	Wingless-related integration site 16 **
Wnt2	Mm00470018_m1	6951974	Yes	No	0.057	3.047	0.082	3.260	0.122	3.219	Wingless-related integration site 2 **
Wnt2b	Mm00437330_m1	6907887	Yes	No	0.009	2.189	0.024	2.030	0.077	1.590	Wingless-related integration site 2b **
Wnt3a	Mm00437337_m1	6788662	Yes	No	0.457	1.199	0.103	1.728	0.684	1.159	Wingless-related integration site 3a **
Wnt6	Mm00437353_m1	6750567	Yes	No	0.011	3.166	0.032	2.668	0.064	2.352	Wingless-related integration site 6 **
Wnt7a	Mm00437355_m1	6955539	Yes	No	0.060	2.117	0.131	1.865	0.395	1.456	Wingless-related integration site 7a **
Wnt7b	Mm00437357_m1	6837582	Yes	No	0.072	2.297	0.050	3.623	0.237	1.912	Wingless-related integration site 7b **

Putatively involved

Gene symbol	Taqman assay ID	Affymetrix ID	Present on Cart V1	Present on Cart V2	E14vsP90 Stepup P-value	E14vsP90 Fold change	E16vsP90 Stepup P-value	E16vsP90 Fold change	P1vsP90 Stepup P-value	P1vsP90 Fold change	Function
Arsa	Mm00802173_g1	6837827	Yes	Yes	0.445	0.741	0.876	0.936	0.710	0.805	Desulfation of cerebroside
Arsg	Mm00546931_m1	6784787	Yes	Yes	0.041	0.616	0.094	0.665	0.885	1.043	Paralog of Arsa
B3galnt1	Mm00480272_s1	6905897	Yes	No	0.069	2.109	0.411	1.331	0.629	1.269	Transferring UDP-GalNAc or UDP-GlcNAc to N-GlcNAc, Galactose or N-GalNAc
B4galnt3	Mm01189804_m1	6956937	Yes	No	0.012	0.411	0.064	0.591	0.111	0.619	Transferring UDP-GalNAc or UDP-GlcNAc to N-GlcNAc, Galactose or N-GalNAc
B4galnt4	Mm00625968_g1	6965153	Yes	No	0.018	3.370	0.519	1.221	0.739	1.166	Transferring UDP-GalNAc or UDP-GlcNAc to N-GlcNAc, Galactose or N-GalNAc
C1galt1	Mm00473986_m1	6943991	Yes	Yes	0.296	0.634	0.176	0.494	0.519	0.674	Transferring UDP-Gal to N-GalNAc.
C1galt1c1	Mm00480676_m1	7016427	Yes	Yes	0.845	1.117	0.533	0.727	0.706	0.753	C1GALT1-specific chaperone 1
Chst1	Mm00517855_m1	6879140	Yes	Yes	0.207	1.301	0.034	2.281	0.143	1.591	Involved in sulfation of keratan sulfate
Gla	Mm00516323_m1	7019519	Yes	Yes	0.290	0.548	0.149	0.361	0.520	0.598	Hydrolysis of galactose units
Glb1	Mm00515342_m1	6992632	Yes	Yes	0.411	1.327	0.393	1.359	0.973	0.979	Hydrolysis of galactose units
Glb1l2	Mm00464245_m1	6994133	Yes	Yes	0.011	0.128	0.069	0.319	0.100	0.319	Hydrolysis of galactose units
Gnpda1	Mm01254913_g1	6864789	Yes	Yes	0.545	0.817	0.545	0.815	0.633	0.793	Conversion of GlcNAc-6-P into fructose-6-P
Gnpda2	Mm00503573_m1	6938963	Yes	Yes	0.991	1.006	0.237	0.609	0.939	1.053	Conversion of GlcNAc-6-P into fructose-6-P
Khk	Mm00434647_m1	6929655	Yes	No	0.424	0.814	0.427	0.809	0.739	1.140	Phosphorylating fructose to fructose-1-P
Kl	Mm00502002_m1	6935777	Yes	Yes	0.489	1.055	0.031	1.411	0.977	1.004	Putative glucuronidase
Klb	Mm00473122_m1	6931257	Yes	Yes	0.013	0.341	0.988	0.996	0.113	1.827	Putative glucuronidase
Renbp	Mm00491720_m1	7017604	Yes	No	0.717	0.778	0.394	0.557	0.853	0.818	Concersion of N-GlcNAc to N-acetylmannosamine
Slc17a5	Mm00555344_m1	6997114	Yes	Yes	0.013	0.738	0.028	0.733	0.165	0.870	Transport of GlcA from lysosome to cytoplasm
Slc26a3	Mm00445313_m1	6794219	Yes	No	0.036	0.818	0.411	0.944	0.166	0.867	Transferring chloride ions across the cell membrane in exchange for bicarbonate ions
Slc26a4	Mm00442308_m1	6799842	Yes	No	0.042	0.861	0.153	1.101	0.368	0.935	Transferring chloride ions across the cell membrane
Slc26a5	Mm00446145_m1	6936587	Yes	Yes	0.929	0.984	0.194	1.258	0.703	1.089	Protein functions as a molecular motor in motile outer hair cells of the cochlea
Slc35a1	Mm00442341_m1	6920732	Yes	Yes	0.034	1.984	0.960	0.987	0.602	0.843	Putative CMP-Sialic Acid Transporter
Ugt8a	Mm00495930_m1	6909083	Yes	Yes	0.961	1.005	0.107	0.840	0.298	0.890	Transferring galactose to ceramide

* Some genes were *not available* on the used exon array version.

Supplementary data table 6: Differentially expressed genes found using Taqman low density arrays for the *Glce* knockout and HSPEtg mouse compared to wildtype.

Production of precursors

Gene symbol	Taqman Assay ID	GLCE knockout mouse			HPSEtg mouse			Function
		Reference gene	P-value	Relative fold expression	Reference gene	P-value	Relative fold expression	
Adpgk	Mm00511302_m1	*18SRNA*	0.629	0.718	*Tbp*	0.409	0.318	Phosphorylation of glucose into glucose-6-P
Gale	Mm00617772_g1	*18SRNA*	0.742	0.887	*Tbp*	0.503	1.628	Conversion of UDP-GlcNAc to UDP-GalNAc
Galk1	Mm00444182_m1	*18SRNA*	0.700	0.838	*Tbp*	0.314	1.495	Phosphorylation of Galactose into Galactose-1-P
Galk2	Mm00613268_m1	*18SRNA*	0.552	0.772	*Tbp*	0.964	1.023	Phosphorylation of N-Acetylgalactosamine (GalNAc) into GalNAc-1P)
Galt	Mm00489459_g1	*18SRNA*	0.299	1.485	*Tbp*	0.616	0.744	UDP-activation of Glucose-1-P and Galactose-1-P
Gck	Mm00439129_m1		*Not detected*			*Not detected*		Phosphorylation of glucose into glucose-6-P
Gfpt1	Mm00600127_m1	*18SRNA*	0.130	0.611	*Tbp*	0.768	0.800	Conversion of fructose-6-P into glucosamine-6-P (Glc-6-P)
Gfpt2	Mm00496565_m1	*18SRNA*	*0.080*	*1.629*	*Tbp*	0.433	0.701	Conversion of fructose-6-P into Glc-6-P
Gnpnat1	Mm00834602_mH	*18SRNA*	***0.033***	***0.468***	*Tbp*	0.295	0.713	Conversion of Glc-6-P into N-Acetylglucosamine-6-P (GlcNAc-6-P)
Gpi1	Mm02026122_g1	*18SRNA*	0.438	1.286	*ActB*	0.530	1.406	Isomerisation of glucose-6-P into fructose-6-P
Hk1	Mm00439344_m1	*18SRNA*	0.563	0.857	*Tbp*	0.475	0.625	Phosphorylation of glucose into glucose-6-P
Hk2	Mm00443385_m1	*18SRNA*	0.369	1.420	*Tbp*	0.345	0.342	Phosphorylation of glucose into glucose-6-P
Miox	Mm00480214_m1		*Not detected*			*Not detected*		Conversion of Myo-inositol into UDP-GlcA
Nagk	Mm00479878_m1	*18SRNA*	0.554	0.818	*Tbp*	0.683	1.221	Phosphorylation of GlcNAc to GlcNAC-6-P
Papss1	Mm00442283_m1	*18SRNA*	0.319	0.646	*Tbp*	0.873	1.068	Synthesis of PAPS complex
Papss2	Mm00442295_m1	*18SRNA*	0.100	0.566	*Tbp*	0.492	0.641	Synthesis of PAPS complex
Pgm1	Mm00804141_m1	*18SRNA*	*0.011*	*0.681*	*Tbp*	0.145	0.292	Conversion of glucose-6-P into glucose-1-P
Pgm2	Mm00728285_s1	*18SRNA*	0.114	2.758	*ActB*	0.977	1.034	Conversion of glucose-6-P into glucose-1-P
Pgm3	Mm00459270_m1	*18SRNA*	0.210	0.631	*Tbp*	0.763	0.840	Conversion of GlcNAc-6-P into GlcNAc-1-P
Pgm5	Mm00723432_m1	*18SRNA*	0.703	0.867	*Tbp*	0.617	0.596	Conversion of glucose-6-P into glucose-1-P
Slc13a1	Mm00490339_m1		*Not detected*			*Not detected*		Transport of sulphate from the extracellular space to cytoplasm
Slc13a4	Mm00556495_m1		*Not detected*			*Not detected*		Transport of sulphate from the extracellular space to cytoplasm
Slc13a5	Mm01334459_m1		*Not detected*			*Not detected*		Transport of sulphate from the extracellular space to cytoplasm
Slc26a11	Mm00626632_m1	*18SRNA*	0.762	1.069	*Tbp*	0.679	1.511	Transport of sulphate from the extracellular space to cytoplasm
Slc26a2	Mm00432896_m1		*Not detected*			*Not detected*		Transport of sulphate from the extracellular space to cytoplasm
Slc26a6	Mm00506742_m1	*18SRNA*	0.803	0.953	*Tbp*	0.236	2.217	Transport of sulphate from the extracellular space to cytoplasm
Slc26a7	Mm00524162_m1	*18SRNA*	0.269	0.465	*Tbp*	0.751	1.509	Transport of sulphate from the extracellular space to cytoplasm
Slc26a8	Mm00524836_m1		*Not detected*			*Not detected*		Transport of sulphate from the extracellular space to cytoplasm
Slc26a9	Mm00628490_m1	*18SRNA*	0.726	0.898		*Not detected*		Transport of sulphate from the extracellular space to cytoplasm
Slc2a1	Mm01192270_m1	*18SRNA*	0.413	1.423	*Tbp*	0.439	0.476	Sugar transport across plasma membrane
Slc2a10	Mm00453716_m1	*18SRNA*	0.684	0.619	*Tbp*	0.927	0.912	Sugar transport across plasma membrane
Slc2a12	Mm00619244_m1	*18SRNA*	0.888	1.072	*Tbp*	0.872	0.820	Sugar transport across plasma membrane
Slc2a2	Mm00446230_g1		*Not detected*			*Not detected*		Sugar transport across plasma membrane
Slc2a3	Mm00441483_m1	*18SRNA*	0.581	1.493		*Not detected*		Sugar transport across plasma membrane
Slc2a4	Mm01245507_g1	*18SRNA*	0.086	2.526	*Tbp*	0.656	0.540	Sugar transport across plasma membrane
Slc2a5	Mm00600311_m1		*Not detected*		*Tbp*	0.961	1.065	Sugar transport across plasma membrane
Slc2a6	Mm00554217_m1	*18SRNA*	0.553	0.596	*Tbp*	0.892	0.875	Sugar transport across plasma membrane
Slc2a8	Mm00444634_m1	*18SRNA*	*0.078*	*1.981*	*Tbp*	0.411	0.435	Sugar transport across plasma membrane
Slc2a9	Mm01211147_m1	*18SRNA*	0.185	0.499	*Tbp*	0.775	1.122	Sugar transport across plasma membrane

Slc35a2	Mm00446504_m1	*18SRNA*	0.600	0.867	*Tbp*	0.992	1.008	Transport from cytoplasm to Golgi system of UDP-GalNAc and UDP-Galactose
Slc35a3	Mm00523288_m1	*18SRNA*	0.240	0.584	*Tbp*	0.408	0.502	Transport from cytoplasm to Golgi system of UDP-GlcNAc
Slc35a4	Mm00782997_s1	*18SRNA*	0.600	1.277	*Tbp*	0.527	1.312	Transport from cytoplasm to Golgi system of UDP-GalNAc and UDP-Galactose
Slc35b1	Mm00450258_m1	*18SRNA*	0.615	0.879	*Tbp*	0.883	1.067	Transport from cytoplasm to Golgi system of UDP-GalNAc and UDP-Galactose
Slc35b2	Mm01269910_m1	*18SRNA*	0.912	0.970	*Tbp*	0.388	0.661	Transport of PAPS comples from cytoplasm to Golgi system
Slc35b3	Mm00506268_m1	*18SRNA*	0.299	0.337	*Tbp*	0.256	2.218	Transport of PAPS comples from cytoplasm to Golgi system
Slc35b4	Mm00480588_m1	*18SRNA*	*0.074*	*0.507*	*Tbp*	0.437	0.391	Transport from cytoplasm to Golgi system of UDP-GlcNAc and UDP-Xylose
Slc35d1	Mm00624959_m1	*18SRNA*	0.153	0.738	*Tbp*	0.868	1.097	Transport from cytoplasm to Golgi system of UDP-GalNAc and UDP-GlcA
Slc35d2	Mm01304830_m1	*18SRNA*	0.971	0.975	*Tbp*	0.406	1.235	Transport from cytoplasm to Golgi system of UDP-GlcNAc
Slc35d3	Mm01717003_m1		*Not detected*			*Not detected*		Paralog of Slc35d1
Uap1	Mm00769477_m1	*18SRNA*	0.777	1.107	*Tbp*	0.952	1.043	UDP-activation of GlcNAc-1-P and GalNAc-1-P
Ugdh	Mm00447643_m1	*18SRNA*	0.499	0.682	*Tbp*	0.578	1.451	Conversion of UDP-glucose into UDP-glucuronic acid (UDP-GlcA)
Ugp2	Mm00454826_m1	*18SRNA*	0.650	0.848	*Tbp*	0.979	1.021	UDP-activation of Glucose-1-P
Uxs1	Mm00550735_m1	*18SRNA*	0.163	0.584	*Tbp*	0.577	0.825	Conversion of UDP-GlcA into UDP-xylose

Core proteins

**Gene symbol**	**Taqman Assay ID**	**GLCE knockout mouse**			**HPSEtg mouse**			**Function**
		**Reference gene**	**P-value**	**Relative fold expression**	**Reference gene**	**P-value**	**Relative fold expression**	
Acan	Mm00545807_m1	*18SRNA*	***0.005***	***0.242***	*Tbp*	***0.055***	***2.478***	Proteoglycan carring CS or DS
Agrn	Mm01264855_m1	*18SRNA*	0.639	0.885	*Tbp*	0.834	1.145	Proteoglycan carring HS present in ECM
Aspn	Mm00445945_m1	*18SRNA*	***0.010***	***0.382***	*Tbp*	0.704	0.709	Proteoglycan carring CS or DS
Bcan	Mm00476090_m1		*Not detected*			*Not detected*		Proteoglycan carring CS or DS
Bgn	Mm00455918_m1	*18SRNA*	0.721	0.802	*Tbp*	0.564	0.640	Proteoglycan carring CS or DS
Cd44	Mm01277164_m1	*18SRNA*	0.337	0.567	*Tbp*	0.609	0.818	Proteoglycan carring HS and hyaluronic acid on cell surface
Col18a1	Mm00487131_m1	*18SRNA*	0.893	1.029	*Tbp*	0.344	0.563	Proteoglycan carring HS present in basement membrane
Col9a2	Mm00483872_m1	*18SRNA*	0.444	2.241		*Not detected*		Proteoglycan carring CS or DS
Cspg4	Mm00507256_m1	*18SRNA*	0.707	0.870	*Tbp*	0.718	0.740	Proteoglycan carring CS or DS
Cspg5	Mm00516549_m1	*18SRNA*	0.469	0.670	*Tbp*	0.369	0.001	Proteoglycan carring CS or DS
Dcn	Mm03003496_s1	*18SRNA*	0.561	0.680	*ActB*	0.517	1.547	Proteoglycan carring CS or DS
Epyc	Mm00514611_m1		*Not detected*			*Not detected*		Proteoglycan carring CS or DS
Gpc1	Mm00497305_m1	*18SRNA*	0.662	0.855	*Tbp*	0.326	0.373	Proteoglycan carring HS present on cell surface via GPI-anchor
Gpc2	Mm00549650_m1	*18SRNA*	0.959	0.960		*Not detected*		Proteoglycan carring HS present on cell surface via GPI-anchor
Gpc3	Mm00516722_m1	*18SRNA*	0.740	0.883	*Tbp*	0.876	1.086	Proteoglycan carring HS present on cell surface via GPI-anchor
Gpc4	Mm00515035_m1	*18SRNA*	0.517	0.777	*Tbp*	0.516	0.708	Proteoglycan carring HS present on cell surface via GPI-anchor
Gpc5	Mm00615599_m1		*Not detected*					Proteoglycan carring HS present on cell surface via GPI-anchor
Gpc6	Mm00516235_m1	*18SRNA*	0.140	0.628	*Tbp*	0.345	0.605	Proteoglycan carring HS present on cell surface via GPI-anchor
Hmmr	Mm00469183_m1	*18SRNA*	0.271	0.507	*Tbp*	0.230	0.739	Hyaluronan-Mediated Motility Receptor
Hspg2	Mm01181165_m1	*18SRNA*	0.934	1.029	*Tbp*	0.629	0.679	Proteoglycan carring HS present in ECM
Ncan	Mm00484007_m1		*Not detected*			*Not detected*		Proteoglycan carring CS or DS
Sdc1	Mm00448918_m1	*18SRNA*	0.435	0.691	*Tbp*	0.448	0.452	Proteoglycan carring HS present on cell surface
Sdc2	Mm00484718_m1	*18SRNA*	0.145	0.624	*Tbp*	0.909	0.929	Proteoglycan carring HS present on cell surface
Sdc3	Mm01179831_m1	*18SRNA*	0.851	1.065	*Tbp*	0.534	0.682	Proteoglycan carring HS present on cell surface
Sdc4	Mm00488527_m1	*18SRNA*	0.513	0.779	*ActB*	*0.032*	*1.291*	Proteoglycan carring HS present on cell surface
Smc3	Mm00484015_m1	*18SRNA*	0.681	0.692	*Tbp*	0.788	1.244	Proteoglycan carring CS or DS
Srgn	Mm01169070_m1	*18SRNA*	0.417	0.680	*Tbp*	0.809	0.846	Proteoglycan carring CS or DS
Vcan	Mm01283063_m1	*18SRNA*	0.928	1.023	*Tbp*	0.641	0.875	Proteoglycan carring CS or DS

Preparation of linkage region

Gene symbol	Taqman Assay ID	GLCE knockout mouse			HPSEtg mouse			Function
		Reference gene	P-value	Relative fold expression	Reference gene	P-value	Relative fold expression	
B3galt6	Mm00504458_s1	*18SRNA*	0.472	0.688	*Tbp*	0.291	0.630	Transferring UDP-galactose to galactose-xylose on proteoglycan
B3gat1	Mm00661499_m1		*Not detected*			*Not detected*		Transferring UDP-GlcA to galactose-galactose-xylose on proteoglycan
B3gat2	Mm00549042_m1		*Not detected*			*Not detected*		Transferring UDP-GlcA to galactose-galactose-xylose on proteoglycan
B3gat3	Mm00470389_m1	*18SRNA*	0.287	1.317	*Tbp*	0.755	0.850	Transferring UDP-GlcA to galactose-galactose-xylose on proteoglycan
B4galt2	Mm00479556_m1	*18SRNA*	0.865	0.948	*Tbp*	0.858	0.913	Transfering UDP-galactose to GlcNAc, Glc, and Xyl.
B4galt7	Mm00461357_m1	*18SRNA*	0.989	1.004	*Tbp*	0.834	0.816	Transferring UDP-galactose to xylose on proteoglycan
Xylt1	Mm00558690_m1	*18SRNA*	0.976	0.982	*Tbp*	0.990	1.007	Transferring UDP-xylose to proteoglycan
Xylt2	Mm00461181_m1	*18SRNA*	0.814	0.957	*Tbp*	0.879	0.912	Transferring UDP-xylose to proteoglycan

Glycosaminoglycan chain polymerisation

Gene symbol	Taqman Assay ID	GLCE knockout mouse			HPSEtg mouse			Function
		Reference gene	P-value	Relative fold expression	Reference gene	P-value	Relative fold expression	
Chpf	Mm01262239_g1	*18SRNA*	0.711	1.153	*Tbp*	0.941	0.942	Transferring UDP-GlcA to GalNAc at end of sugar chain (CS)
Chsy1	Mm01319178_m1	*18SRNA*	0.983	1.005	*Tbp*	0.944	1.022	Transferring UDP-GlcA to GalNAc at end of sugar chain (CS)
Chsy3	Mm01545329_m1	*18SRNA*	0.995	0.997	*Tbp*	0.869	1.086	Transferring UDP-GlcA to GalNAc at end of sugar chain (CS)
Csgalnact1	Mm00555164_m1	*18SRNA*	0.661	0.783	*Tbp*	0.203	0.506	Transferring UDP-GalNAc to GlcA at end of sugar chain (CS)
Csgalnact2	Mm00513340_m1	*18SRNA*	***0.049***	***0.431***	*Tbp*	0.845	0.858	Transferring UDP-GalNAc to GlcA at end of sugar chain (CS)
Ext1	Mm00468769_m1	*18SRNA*	0.590	0.826	*Tbp*	0.943	1.024	Transferring UDP-GlcNAc to GlcA at end of sugar chain (HS)
Ext2	Mm00468775_m1	*18SRNA*	0.634	0.852	*Tbp*	0.579	0.626	Transferring UDP-GlcNAc to GlcA at end of sugar chain (HS)
Extl1	Mm00621977_s1	*18SRNA*	0.519	1.269	*Tbp*	0.347	1.426	Transferring UDP-GlcA to GlcNAc at end of sugar chain (HS)
Extl2	Mm00469621_m1	*18SRNA*	0.377	0.703	*Tbp*	0.819	0.773	Transferring UDP-GlcA to GlcNAc at end of sugar chain (HS)
Extl3	Mm00516994_m1	*18SRNA*	0.467	0.825	*Tbp*	0.318	0.482	Transferring UDP-GlcA to GlcNAc at end of sugar chain (HS)
Has1	Mm00468496_m1	*18SRNA*	0.996	0.999	*Tbp*	0.395	0.000	Polymerisation of hyaluronic acid
Has2	Mm00515089_m1	*18SRNA*	0.158	0.538	*Tbp*	0.900	0.871	Polymerisation of hyaluronic acid
Has3	Mm00515091_m1	*18SRNA*	0.800	0.745		*Not detected*		Polymerisation of hyaluronic acid

Glycosaminoglycan chain modification

**Gene symbol**	**Taqman Assay ID**	**GLCE knockout mouse**			**HPSEtg mouse**			**Function**
		**Reference gene**	**P-value**	**Relative fold expression**	**Reference gene**	**P-value**	**Relative fold expression**	
Chst11	Mm00517563_m1	*18SRNA*	0.273	0.815	*Tbp*	0.789	0.794	Sulfation at the 4-O postion of GalNAc untis (CS)
Chst12	Mm00546416_s1	*18SRNA*	0.593	1.273	*Tbp*	0.938	0.948	Sulfation at the 4-O postion of GalNAc untis (CS)
Chst13	Mm01186255_s1		*Not detected*			*Not detected*		Sulfation at the 4-O postion of GalNAc untis (CS)
Chst14	Mm00511291_s1	*18SRNA*	0.633	0.812	*Tbp*	0.784	1.146	Sulfation at the 4-O postion of GalNAc untis (CS)
Chst15	Mm00513227_m1	*18SRNA*	0.946	0.982	*Tbp*	0.560	0.555	Sulfation at the 6-O postion of GalNAc untis (CS)
Chst2	Mm00490018_g1	*18SRNA*	0.774	1.228	*Tbp*	0.692	1.809	Sulfation at the 6-O postion of GalNAc untis (CS)
Chst3	Mm00489736_m1	*18SRNA*	0.674	1.266	*Tbp*	0.864	0.784	Sulfation at the 6-O postion of GalNAc untis (CS)
Chst5	Mm00517342_m1		*Not detected*			*Not detected*		Sulfation at the 6-O postion of GalNAc untis (CS)
Chst7	Mm00491466_m1	*18SRNA*	0.952	1.030	*Tbp*	0.991	1.011	Sulfation at the 6-O postion of GalNAc untis (CS)
Chst8	Mm00558321_m1	*18SRNA*	0.737	0.805		*Not detected*		Sulfation at the 4-O postion of GalNAc untis (CS)
Chst9	Mm01722279_m1		*Not detected*			*Not detected*		Sulfation at the 4-O postion of GalNAc untis (CS)
Dse	Mm00552923_m1	*18SRNA*	0.249	0.566	*Tbp*	0.454	0.559	Epimerisation of GlcA unit into iduronic acid unit (IdoA) (CS)
Glce	Mm00473667_m1	*18SRNA*	***0.013***	***0.079***	*Tbp*	0.550	0.700	Epimerisation of GlcA unit into iduronic acid unit (IdoA) (HS)
Hs2st1	Mm00478684_m1	*18SRNA*	*0.072*	*0.530*	*Tbp*	0.605	0.803	Sulfation at the 2-O position of GlcA and IdoA units (HS)
Hs3st1	Mm01964038_s1	*18SRNA*	*0.019*	*0.571*	*Tbp*	0.335	0.679	Sulfation at the 3-O position of GlcNAc and GlcNS units sulfated at the 6-O position (HS)
Hs3st2	Mm00616933_m1		*Not detected*			*Not detected*		Sulfation at the 3-O position of GlcNAc and GlcNS units sulfated at the 6-O position (HS)
Hs3st3a1	Mm00780907_s1	*18SRNA*	*0.032*	*1.742*	*Tbp*	0.868	1.108	Sulfation at the 3-O position of GlcNAc and GlcNS units sulfated at the 6-O position (HS)
Hs3st3b1	Mm00479621_m1	*18SRNA*	0.673	0.883	*Tbp*	0.672	0.675	Sulfation at the 3-O position of GlcNAc and GlcNS units sulfated at the 6-O position (HS)
Hs3st5	Mm01192940_m1		*Not detected*			*Not detected*		Sulfation at the 3-O position of GlcNAc and GlcNS units sulfated at the 6-O position (HS)
Hs3st6	Mm01299930_m1	*18SRNA*	0.409	0.655	*Tbp*	0.174	1.727	Sulfation at the 3-O position of GlcNAc and GlcNS units sulfated at the 6-O position (HS)
Hs6st1	Mm01229698_s1	*18SRNA*	0.981	0.983	*Tbp*	0.791	1.076	Sulfation at the 6-O position of GlcNAc and N-sulfated-glucuronic acid units (GlcNS) (HS)
Hs6st2	Mm00479296_m1	*18SRNA*	0.492	1.316	*Tbp*	0.309	0.606	Sulfation at the 6-O position of GlcNAc and GlcNS units (HS)
Hs6st3	Mm00479297_m1		*Not detected*			*Not detected*		Sulfation at the 6-O position of GlcNAc and GlcNS units (HS)
Ndst1	Mm00447005_m1	*18SRNA*	0.445	0.831	*Tbp*	0.420	0.507	N-de-acetylation and N-sulfation of GlcNAc unit (HS)
Ndst2	Mm00447818_m1	*18SRNA*	0.916	1.052	*Tbp*	0.643	0.689	N-de-acetylation and N-sulfation of GlcNAc unit (HS)
Ndst3	Mm00453178_m1	*18SRNA*	0.773	0.877	*Tbp*	0.503	2.028	N-de-acetylation and N-sulfation of GlcNAc unit (HS)
Ndst4	Mm00480767_m1		*Not detected*			*Not detected*		N-de-acetylation and N-sulfation of GlcNAc unit (HS)
Sulf1	Mm00552283_m1	*18SRNA*	0.324	1.358	*Tbp*	0.883	1.140	Desulfation of the 6-O positon of 6-O sulfated GlcNAc and GlcNS units (HS)
Sulf2	Mm01248026_m1	*18SRNA*	0.380	0.642	*Tbp*	0.770	1.107	Desulfation of the 6-O positon of 6-O sulfated GlcNAc and GlcNS units (HS)
Sumf1	Mm00461213_m1	*18SRNA*	0.311	0.638	*Tbp*	0.758	0.859	Cofactor for Sulf1 and Sulf 2 (HS)
Sumf2	Mm01197721_m1	*18SRNA*	0.993	1.004	*Tbp*	0.763	0.850	Cofactor for Sulf1 and Sulf 2 (HS)
Ust	Mm00616790_m1	*18SRNA*	0.447	0.684	*Tbp*	0.754	1.382	Sulfation at the 2-O position of GlcA and IdoA units (CS)

Glycosaminoglycan chain degradation

Gene symbol	Taqman Assay ID	GLCE knockout mouse			HPSEtg mouse			Function
		Reference gene	P-value	Relative fold expression	Reference gene	P-value	Relative fold expression	
Arsb	Mm00503655_s1	*18SRNA*	0.840	0.904	*Tbp*	0.703	1.340	Desulfation of the 4-O position of 4-O sulfated GalNAc units (CS)
Arsj	Mm00557970_m1	*18SRNA*	0.788	0.916		*Not detected*		Desulfation of the 4-O position of 4-O sulfated GalNAc units (CS)
Arsk	Mm00513099_m1	*18SRNA*	0.417	0.556	*Tbp*	0.776	1.330	Desulfation of proteoglycans, paralog of IDS
Galns	Mm00489576_m1	*18SRNA*	0.795	0.920	*Tbp*	0.589	0.566	Desulfation of the 6-O positon of 6-O sulfated GalNAc units (CS)
Gns	Mm00659592_m1	*18SRNA*	0.889	0.935	*Tbp*	0.572	0.657	Desulfation of the 6-O positon of 6-O sulfated GlcNAc and GlcNS units (HS)
Gusb	Mm01197698_m1	*18SRNA*	0.404	0.747	*Tbp*	0.876	0.886	Hydrolysation of GlcA from sugar chain (HS and Hyaluronic acid and CS)
Hexa	Mm00599877_m1	*18SRNA*	0.850	0.894	*Tbp*	0.667	1.542	Hydrolysation of GalNAc units (CS) and GlcNAc untis (hyaluronic acid) from sugar chain
Hexb	Mm00599880_m1	*18SRNA*	0.536	0.831	*Tbp*	0.251	0.591	Hydrolysation of GalNAc units (CS) and GlcNAc untis (hyaluronic acid) from sugar chain
Hgsnat	Mm00519485_m1	*18SRNA*	0.850	1.077	*Tbp*	0.717	0.869	Acetylation of desulfated GlcNS units (HS)
Hpse	Mm00461768_m1	*18SRNA*	0.622	0.850	*Tbp*	0.310	0.479	Degradation of HS chains extracellularly
Hyal2	Mm00477731_m1	*18SRNA*	0.936	1.040	*Tbp*	0.411	0.429	Degradation of hyaluronic acid chain
Hyal3	Mm00662097_m1		*Not detected*			*Not detected*		Degradation of hyaluronic acid chain
Ids	Mm00494868_m1	*18SRNA*	0.880	0.949	*Tbp*	0.433	0.497	Desulfation of 2-O sulfated IdoA and GlcA units (HS and CS)
Idua	Mm00515143_m1	*18SRNA*	0.662	1.309	*Tbp*	0.512	0.535	Hydrolysation of IdoA unit from sugar chain (HS and CS)
Mgea5	Mm00452409_m1	*18SRNA*	0.287	0.687	*Tbp*	0.525	0.633	Degradation of hyaluronic acid chain
Naga	Mm00476274_m1	*18SRNA*	0.562	1.215	*Tbp*	0.802	0.877	Hydrolysis N-GalNAc moieties from glycoconjugates.
Naglu	Mm00479175_m1	*18SRNA*	0.820	0.880	*Tbp*	0.462	0.393	Hydrolysation of GlcNAc from sugar chain (HS)
Nat6;Hyal1	Mm00480053_m1	*18SRNA*	0.743	0.871	*Tbp*	0.444	0.440	Degradation of hyaluronic acid chain
Sgsh	Mm00450747_m1	*18SRNA*	0.400	0.715	*Tbp*	0.297	0.524	Desulfation of GlcNS units (HS)

Growth factors

Gene symbol	Taqman Assay ID	GLCE knockout mouse			HPSEtg mouse			
		Reference gene	P-value	Relative fold expression	Reference gene	P-value	Relative fold expression	Function
Ctgf	Mm01192933_g1	*18SRNA*	0.172	0.508	*Tbp*	0.693	0.810	Connective tissue growth factor, embryonic growth factor in skin development **
Fgf10	Mm00433275_m1	*18SRNA*	0.121	1.291	*Tbp*	0.606	0.797	Fibroblast growth factor 10 **
Fgf2	Mm01285715_m1	*18SRNA*	0.139	0.908	*Tbp*	0.983	0.980	Fibroblast growth factor 2 **
Fgf4	Mm00438917_m1		*Not detected*			*Not detected*		Growth factor (Uijtdewilligen et al, 2013)**
Fgf7	Mm00433291_m1	*18SRNA*	0.151	0.725	*Tbp*	0.539	0.681	Fibroblast growth factor 7 **
Fgf8	Mm00438921_m1		*Not detected*			*Not detected*		Fibroblast growth factor 8 **
Figf	Mm01131929_m1	*18SRNA*	0.117	1.412	*Tbp*	0.777	0.796	C-fos induced growth factor **
Hbegf	Mm00439305_g1	*18SRNA*	0.150	0.719	*Tbp*	0.652	0.633	Heparin-binding epidermal growth factor **
Pdgfa	Mm01205760_m1	*18SRNA*	0.129	1.081	*Tbp*	0.202	1.459	Platelet-derived growth factor a **
Pdgfb	Mm01298578_m1	*18SRNA*	0.122	1.267	*Tbp*	0.986	0.987	Platelet-derived growth factor b **
Pdgfc	Mm03053266_s1	*18SRNA*	0.139	0.919	*Tbp*	0.831	0.811	Platelet-derived growth factor c **
Pdgfd	Mm00546829_m1	*18SRNA*	0.114	1.579	*Tbp*	0.344	0.592	Platelet-derived growth factor d **
Shh	Mm00436527_m1	*18SRNA*	0.139	0.918	*Tbp*	0.506	2.393	Sonic hedgehog **
Tgfb1	Mm01178819_m1	*18SRNA*	0.157	0.704	*Tbp*	0.924	0.956	Transforming growth factor beta 1 **
Tgfb2	Mm01321739_m1	*18SRNA*	0.161	0.627	*Tbp*	0.808	0.832	Transforming growth factor beta 2 **
Tgfb3	Mm01307950_m1	*18SRNA*	0.133	1.019	*Tbp*	0.588	0.706	Transforming growth factor beta 3 **
Vegfa	Mm01281449_m1	*18SRNA*	0.108	1.683	*Tbp*	0.810	1.233	Vascular endothelial growth factor a **
Vegfb	Mm00442102_m1	*18SRNA*	0.105	1.693	*Tbp*	0.746	1.376	Vascular endothelial growth factor b **
Vegfc	Mm01202432_m1	*18SRNA*	0.135	0.981	*Tbp*	0.758	0.771	Vascular endothelial growth factor c **

Putatively involved

Gene symbol	Taqman Assay ID	GLCE knockout mouse			HPSEtg mouse			
		Reference gene	P-value	Relative fold expression	Reference gene	P-value	Relative fold expression	Function
18S	Hs99999901_s1		*Reference gene*			*Reference gene*		Reference gene
Actb	Mm00607939_s1		*Reference gene*			*Reference gene*		Reference gene
Arsa	Mm00802173_g1	*18SRNA*	0.336	1.195	*Tbp*	0.775	0.738	Desulfation of cerebroside
Arsg	Mm00546931_m1	*18SRNA*	0.945	0.982	*Tbp*	0.895	1.079	Paralog of Arsa
C1galt1	Mm00473986_m1	*18SRNA*	*0.046*	*0.521*	*Tbp*	0.549	0.736	Transferring UDP-Gal to N-GalNAc.
C1galt1c1	Mm00480676_m1	*18SRNA*	0.176	0.510	*Tbp*	0.841	0.899	C1GALT1-specific chaperone 1
Chst1	Mm00517855_m1	*18SRNA*	0.817	1.043	*Tbp*	0.512	0.496	Involved in sulfation of keratan sulfate
Gapdh	Mm03302249_g1		*Reference gene*			*Reference gene*		Reference gene
Gla	Mm00516323_m1	*18SRNA*	0.156	0.753	*Tbp*	0.127	0.536	Hydrolysis of galactose units
Glb1	Mm00515342_m1	*18SRNA*	0.518	1.169	*Tbp*	0.447	0.578	Hydrolysis of galactose units
Glb1l2	Mm00464245_m1	*18SRNA*	0.548	1.180	*Tbp*	0.435	0.357	Hydrolysis of galactose units
Gnpda1;Gm8615	Mm01254913_g1	*18SRNA*	0.402	1.218	*Tbp*	0.324	0.455	Conversion of GlcNAc-6-P into fructose-6-P
Gnpda2	Mm00503573_m1	*18SRNA*	*0.001*	*0.641*	*Tbp*	0.174	0.485	Conversion of GlcNAc-6-P into fructose-6-P
Hprt1	Mm01324427_m1		*Reference gene*			*Reference gene*		Reference gene
Kl	Mm00502002_m1	*18SRNA*	0.488	1.579		*Not detected*		Putative glucuronidase
Klb	Mm00473122_m1	*18SRNA*	0.557	4.164	*Tbp*	0.455	1.714	Putative glucuronidase
Slc17a5	Mm00555344_m1	*18SRNA*	0.827	0.939	*Tbp*	0.662	0.758	Transport of GlcA from lysosome to cytoplasm
Slc35a1	Mm00442341_m1	*18SRNA*	*0.008*	*0.536*	*Tbp*	0.840	0.901	Putative CMP-Sialic Acid Transporter
Tbp	Mm01277042_m1		*Reference gene*			*Reference gene*		Reference gene
Ugt8a	Mm00495930_m1	*18SRNA*	0.411	0.628	*Tbp*	0.312	2.966	Transferring galactose to ceramide

* Glc, glucosamine; GlcNAc, N-acetylated glucosamine; Gal, galactosamine; GalNAc, N-acetylated galactosamine; GlcA, glucuronic acid; IdoA, iduronic acid. **Uijtdewilligen *et al* ., 2016.

Genes, for which a signal was not or only partly detected at a given timepoint or multiple timepoints and therefore a fold change and/or p-value could not be calculated based on the available data, are given as 'not detected'. Gene symbol for which all timepoints were classified as 'not detected' do not show a reference gene due to lack of data for a calculation.
